# Genomic comparison of *Staphylococcus aureus* isolates from patients with bacteraemia and infective endocarditis at public hospitals in Gauteng, South Africa

**DOI:** 10.1007/s11274-026-05145-z

**Published:** 2026-07-27

**Authors:** Lz D. Jansen van Vuuren, Thabo Hamiwe, Veronica Ueckermann, Marleen M. Kock, Anel Bosch, Marthie M. Ehlers

**Affiliations:** 1 Department of Medical Microbiology, University of Pretoria, Pretoria, South Africa; 2 Division for Infectious Diseases, Department of Internal Medicine, Steve Biko Academic Hospital and University of Pretoria, Pretoria, South Africa; 3 Department of Medical Microbiology, Tshwane Academic Division, National Health Laboratory Service, Pretoria, South Africa

**Keywords:** Bacteraemia, Genetic relatedness, Infective endocarditis, *Staphylococcus aureus*, Virulence

## Abstract

*Staphylococcus aureus* (*S. aureus*) is a leading cause of bacteraemia and infective endocarditis worldwide, posing significant public health challenges in resource-limited low- and middle-income countries (LMICs). However, whether specific genomic factors influence the development of *S. aureus* infective endocarditis (SAIE) during *S. aureus* bacteraemia (SAB) remains unclear. Thus, the study aimed to determine whether clinical SAIE isolates were genomically distinguishable from SAB isolates originating from public hospitals in Gauteng, South Africa. Seventy-seven (54 SAB, 23 SAIE) bloodstream isolates were characterised to assess antimicrobial susceptibility (VITEK®2 system), biofilm formation (crystal violet assay), virulence genes (multiplex-polymerase chain reactions) and genetic relatedness (pulsed-field gel electrophoresis), while 12 representative isolates underwent whole-genome sequencing. A higher SAIE (29.9%) prevalence than previously reported in South Africa was observed, with cases linked to young male persons who inject drugs (P < 0.01). While the SAB and SAIE groups did not differ in virulence profiles, SAB isolates demonstrated higher resistance rates to gentamicin and clindamycin than SAIE isolates (P < 0.05). The endemic Panton-Valentine leukocidin-positive sequence type (ST) 152, clonal complex (CC) 152 lineage predominated in SAIE isolates, while the pandemic CC8, encompassing ST239 and ST612, was exclusive to SAB isolates. Overall, SAB and SAIE isolates were not clearly distinguished by the markers assessed, suggesting that SAIE development may reflect the combined effects of bacterial traits, host susceptibility and exposure-related risk factors, rather than a distinct SAIE-specific genomic profile. These findings highlight the need for continued genomic surveillance of circulating *S. aureus* lineages to inform targeted infection-control strategies.

**Supplementary Information:**

The online version contains supplementary material available at 10.1007/s11274-026-05145-z.

## Introduction

*Staphylococcus aureus* (*S. aureus*) remains a formidable threat to human health and well-being as a leading cause of bacteraemia and infective endocarditis (IE) worldwide (Agnello et al. 2021). While often transient, unresolved *S. aureus* bacteraemia (SAB) can progress to severe complications, including *S. aureus* IE (SAIE), a deadly localised endocardial infection with high rates of recurrence (Kinney et al. 2022a). The emergence of SAIE has become particularly pronounced in South Africa and is the leading cause of IE among persons who inject drugs (PWIDs) (Pecoraro et al. 2021). With a high proportion of the South African population at risk due to immunocompromising conditions and limited access to quality healthcare, *S. aureus* poses a significant public health concern (Achoki et al. 2022). Despite its clinical significance, the characteristics, outcome-related factors and local epidemiology of SAIE in South African healthcare-associated (HA) settings remain underexplored.

The pathogenic success of *S. aureus* arises from extensive antimicrobial resistance (AMR) mechanisms and virulence factors that facilitate host colonisation, immune evasion and persistence (Kinney et al. 2022a). Cardiac vegetation formation, pathognomonic of SAIE, is promoted by adhesins and coagulases that trigger platelet activation and aggregation (Liesenborghs et al. 2020). These factors include clumping factor A (ClfA), fibronectin-binding protein A (FnBPA), iron-regulated surface determinant protein B (IsdB), serine-aspartate repeat-containing protein E (SdrE), extracellular adherence protein (Eap), coagulase (Coa) and von Willebrand factor-binding protein (vWbp) (Kinney et al. 2022b). In parallel, exoenzymes, haemolysins and superantigens (SAgs) drive the aggressive nature of SAIE (Kinney et al. 2022a). Specific toxins, including beta (β)-haemolysin (Hlb), staphylococcal enterotoxin (SE) C (SEC), the enterotoxin gene cluster [*egc*; SEI, SEM, SEO, SE-like toxin U (SEl-U)] and toxic shock syndrome toxin-1 (TSST-1), are thought to induce disease by altering endothelial cell activity and promoting vegetation (Kinney et al. 2022b). Additionally, SAgs affect SAIE progression by dysregulating immune responses, causing persistent inflammation and cytotoxicity (Salgado-Pabón et al. 2013).

The present study was prompted by conflicting evidence on the ability to distinguish between SAB and SAIE strains. Earlier studies (Salgado-Pabón et al. 2013; Bouchiat et al. 2015) suggested that specific bacterial traits might be linked to SAIE development, whereas a more recent multi-high-income country (HIC) cohort (Bastien et al. 2023) found similar pathogenic potential among SAB and SAIE strains. However, the absence of African isolates from these comparative cohorts, limited local genomic surveillance and contextual differences between South Africa and the HICs assessed made it unclear whether local strain characteristics follow these trends. Therefore, this study aimed to characterise *S. aureus* isolates associated with SAB and SAIE cases at Gauteng public hospitals to determine whether clinical SAIE isolates are genomically distinguishable from non-IE SAB isolates and whether specific factors influence progression to SAIE.

## Materials and methods

### Study setting and collection of *S. aureus* isolates

Ethical approval for the study was obtained from the University of Pretoria (UP), Faculty of Health Sciences Research Ethics Committee (Reference number: 183/2023). The study was conducted at the Department of Medical Microbiology, UP, using *S. aureus* isolates collected between February and November 2023 from a public diagnostic laboratory in Tshwane, South Africa. This diagnostic laboratory provides services to public clinics and hospitals at both primary and tertiary levels within the city of Tshwane and surrounding areas. The *S. aureus* isolates were collected from bacteraemia cases after routine diagnostic specimen processing, which included species identification and antimicrobial susceptibility testing (AST) using the VITEK®2 system (bioMérieux, France). The AST panel comprised susceptibility profiles for ampicillin, benzylpenicillin, cefoxitin (including cefoxitin screen), ciprofloxacin, clindamycin [including inducible clindamycin resistance (ICR)], erythromycin, fusidic acid, gentamicin, linezolid, moxifloxacin, mupirocin, oxacillin, rifampicin, streptomycin, teicoplanin, tetracycline, tigecycline, trimethoprim/sulfamethoxazole (TMP/SMX) and vancomycin. Patient demographic, epidemiological, clinical and microbiological data were retrospectively collected from diagnostic laboratory databases and patient records. Isolate infection type (SAB or SAIE) was assigned based on the patient’s final clinical diagnosis, with the assistance of a clinician. Specifically, SAIE was assigned to cases with a documented diagnosis of IE, whereas SAB was assigned to cases lacking endocardial involvement, with bacteraemia secondary to other foci or general sepsis.

### Molecular species confirmation and virulence characterisation

Seventy-seven non-repeat *S. aureus* bloodstream isolates were streaked onto 5% sheep blood agar (MediaMage, South Africa) and incubated for 24 h at 37 °C (Vacutec, UK). Thereafter, a single pure colony was inoculated into 5 mL sterile brain heart infusion broth (LabM, UK) and incubated with shaking (Stuart Orbital Incubator, UK) for 24 h at 37 °C. Total genomic DNA was extracted using the boiling method with modifications (Barbosa et al. 2016). Briefly, phosphate-buffered saline [PBS, pH 7.2 (Thermo Scientific, USA)] was used instead of a detergent-based lysis buffer and cultures were centrifuged three times (MC-24® Touch, Benchmark, USA): initially at 5,500 × g for 5 s, followed by two additional centrifugation and PBS (Thermo Scientific, USA) wash steps at 5,500 × g for 5 min. Lastly, a sonication step (Transsonic 460, USA) of 15 min at 35 kHz was incorporated to improve cell lysis.

A species identification multiplex polymerase chain reaction (M-PCR) assay was performed to confirm isolates as *S. aureus* and to identify methicillin-resistant *S. aureus* (MRSA) based on the presence of the methicillin-resistance gene (*mecA*). The *S. aureus* isolates were screened for multiple virulence genes previously associated with SAIE, including cytolytic toxins, exoenzymes, regulatory elements, SAgs, secretable expanded-repertoire adhesive molecules (SERAMs) and microbial surface components recognising adhesive matrix molecules (MSCRAMMs). Primer sequences and amplification conditions used in the conventional M-PCR assays are indicated in Table S1 provided in Online Resource 1. A CelTaq Hotstart DNA polymerase and buffer master mix (Celtic Molecular Diagnostics, South Africa) was prepared according to the manufacturer’s instructions. The *S. aureus* ATCC 33591, 29213, 700699, 25923, 24213 and in-house controls were used in the identification and virulence gene detection M-PCR assays, with results interpreted according to expected amplicon band sizes for each target gene, as detailed in Table S1 in Online Resource 1.

### Evaluation of *S. aureus* phenotypic biofilm formation

Biofilm formation was assessed using the crystal violet assay as previously described (Merritt et al. 2005), with modifications that included culturing in 1% glucose-supplemented tryptic soy broth (Oxoid, UK), heat fixation (ProBlot 12S, Labnet, USA) at 65 °C for 1 h prior to staining and the use of PBS (Thermo Scientific, USA) for washing. The *S. aureus* ATCC 12600 was included as a reference strain. Biofilm biomass was quantified by measuring the optical density (OD) at 550 nm (OD₅₅₀) using a PR 4100 Absorbance Microtiter Plate Reader (BioRad, USA). The OD was measured using the following formula: OD cut-off (ODc) = Average OD of negative control + [3 × standard deviation (SD) of the negative control]. The final OD used to characterise biofilm formation was calculated as the average isolate OD – ODc. Isolates were categorised as non (OD ≤ ODc), weak (ODc < OD ≤ 2 × ODc), moderate (2 × ODc < OD ≤ 4 × ODc) or strong (4 × ODc < OD) biofilm formers.

### Pulsed-field gel electrophoresis genotyping

The genetic relatedness of the *S. aureus* isolates was assessed using pulsed-field gel electrophoresis (PFGE) with the *S. aureus* ATCC 12600 strain as a size standard. Plug preparation was done according to McDougal et al. (2003) and the CHEF-DR® III protocol (BioRad, USA). Genomic DNA was digested with *Sma*I (Thermo Scientific, USA) and resolved on a 1.2% (m/v) Seakem agarose gel (Lonza, USA) stained with ethidium bromide [250 μL of 10 mg/mL (Sigma-Aldrich, USA)]. The run conditions were as follows: 21 h at 14 °C with an interval of 5–40 s linear at a constant angle of 120° and a voltage of 200–220 V. Gel images were analysed using GelCompar II (Applied Maths, Belgium), with cluster analysis performed using the Dice coefficient and the dendrogram constructed using the unweighted pair group method with arithmetic mean. An ≥ 80% similarity threshold was used to define and categorise pulsotypes as major (≥ 5 isolates), minor (< 5 isolates) or singletons.

### Whole-genome sequencing of selected *S. aureus* isolates

Due to the high cost of whole-genome sequencing (WGS) in South Africa, only 12 representative *S. aureus* isolates (6 SAB, 6 SAIE) were selected for this analysis. The selection aimed to capture phenotypic and genotypic diversity. Thus, isolates were considered based on AST profiles [ranging from full susceptibility to multidrug resistance (MDR)], number of virulence genes detected (low to high), biofilm-forming capacity (none to strong), patient outcome (a fatal case) and PFGE clustering to represent major clusters, minor clusters, singletons and one un-typeable isolate. The Quick-DNA™ Fungal/Bacterial Miniprep Kit (Zymo Research, USA) was used for DNA extraction according to the manufacturer’s instructions. The DNA quality (A260/A280 ≥ 1.8) was verified using a nano spectrophotometer (Labtron, UK) and submitted to the National Institute for Communicable Diseases (NICD), South Africa, for WGS. Multiplexed, paired-end libraries were prepared using the Nextera DNA Flex Kit (Illumina, USA) and sequenced at 100 × coverage and 2 × 150 bp read length using the NextSeq 2000 (Illumina, USA) platform. Raw reads were examined for quality [FastQC 0.12.0 software (Babraham Bioinformatics, UK)], trimmed and assembled with CLC Genomics Workbench 24.0.1 (Bio-Qiagen, USA) and SPAdes 3.15.4 (Prjibelski et al. 2020). Annotation was performed using the Jekesa pipeline (Kwenda et al. 2020). Species confirmation and characterisation of AMR, virulence, plasmids, mobile genetic elements (MGEs) and genotyping were conducted using the following Centre for Genomic Epidemiology tools: SpeciesFinder (Larsen et al. 2014), ResFinder 4.6.0 (Bortolaia et al. 2020), VirulenceFinder 2.0 (Joensen et al. 2014), MGEFinder (Johansson et al. 2020), PlasmidFinder 2.1 (Carattoli et al. 2014), MLST 2.0 (Larsen et al. 2012), spaTyper (Bartels et al. 2014) and SCC*mec*Finder 1.2 (Kaya et al. 2018). Supplementary databases used included the Comprehensive Antibiotic Resistance Database (Alcock et al. 2023) and the Virulence Factor Database (Liu et al. 2022). Pairwise nucleotide polymorphism (SNP) differences were determined using the Split Kmer Analysis toolkit (Liu et al. 2022).

### Statistical analysis

Continuous variables were summarised as means (range) and medians [interquartile range (IQR)], while categorical data were reported as frequencies, proportions and 95% confidence intervals (CIs). Group comparisons were performed using Fisher’s exact test for categorical data and the Mann–Whitney U test for continuous variables, where data did not meet the assumptions of normality. Correlations were assessed using point-biserial (rpb) and Kendall tau-b (τb) non-parametric tests to explore relationships between infection type (SAB and SAIE) and continuous (biofilm OD) or categorical variables (sex, ward group and risk factors). A binary logistic regression model was used to identify independent predictors of SAIE, evaluating associations with clinical characteristics including intravenous drug use (IVDU), human immunodeficiency virus (HIV) status and hepatitis C co-infection. Statistical analyses were performed using STATA 16.0 (StataCorp, USA), with two-tailed P < 0.05 considered statistically significant and P < 0.01 or P < 0.001 interpreted as indicating stronger evidence against the null hypothesis.

## Results

### Demographic and clinical characteristics

The majority of the *S. aureus* isolates were SAB [70.1% (54/77)], while SAIE accounted for 29.9% (23/77) of the study isolates. The patient demographic and clinical characteristics are presented in Table 1. Isolates with incomplete data for specific variables were excluded from relevant analyses, resulting in data point variability. A significant association was found between infection type and sex (P < 0.001), with SAIE occurring predominantly among male patients [95.7% (22/23)]. The age of SAIE patients ranged from 22 to 45 years (median = 33, IQR = 30–36), while SAB patients ranged from 4 days to 76 years (median = 32, IQR = 23.5–41). Table 2 summarises the logistic regression results for predictors of SAIE. The model was statistically significant (χ^2^ = 14.54, P < 0.01) and explained 26.2% of the variance in infection type. Recreational IVDU emerged as a strong predictor of SAIE (odds ratio = 7.46, 95% CI: 1.24–45.05, P < 0.05), which aligned with univariate findings of PWID-SAIE association (τb = 0.55, P < 0.001). No significant associations were observed for HIV or hepatitis C after adjusting for IVDU.

**Table 1:** Demographical characteristics of *Staphylococcus aureus* bacteraemia and infective endocarditis isolate groups

Characteristic	No. of Isolates with the Characteristic [n = 77 (%)]	No. of Isolates with the Characteristic by Infection Type		Overall P Value	
		Bacteraemia [n = 54 (%)]	Infective Endocarditis [n = 23 (%)]	Subgroup P Value	Overall Group P Value
Sex ^a^					
Male	52 (69.33)	30 (57.69)	22 (95.65)	0.001*	
Female	23 (30.67)	22 (42.31)	1 (4.35)		
Unknown	2	2	0	N/A	
Age Groups ^a^					
< 5 years	7 (10.14)	7 (14.58)	0	0.092	0.049*
5 years–24 years	7 (10.14)	6 (12.50)	1 (4.76)	0.427	
25 years–44 years	44 (63.77)	25 (52.08)	19 (90.48)	0.002*	
45 years–64 years	8 (11.59)	7 (14.58)	1 (4.76)	0.419	
> 65 years	3 (4.35)	3 (6.25)	0	0.548	
Unknown	8	6	2	N/A	
Hospital ^b^					
Hospital 1	25 (32.47)	20 (37.04)	5 (21.74)	0.288	0.001*
Hospital 2	13 (16.88)	13 (24.07)	0	0.008*	
Hospital 3	1 (1.30)	1 (1.85)	0	1.000	
Hospital 4	20 (25.97)	7 (12.96)	13 (56.52)	0.000*	
Hospital 5	8 (10.39)	6 (11.11)	2 (8.70)	1.000	
Hospital 6	5 (6.49)	3 (5.56)	2 (8.70)	0.632	
Hospital 7	5 (6.49)	4 (7.41)	1 (4.35)	1.000	
Wards ^a^					
Emergency	38 (52.78)	19 (38.78)	19 (82.61)	0.000*	0.004*
High dependency	15 (20.83)	14 (28.57)	1 (4.35)	0.028*	
General	17 (23.61)	14 (28.57)	3 (13.04)	0.234	
Outpatient	2 (2.78)	2 (4.08)	0	1.000	
Unknown	5	5	0	N/A	
Risk Factors and Clinical Conditions ^ac^					
Immunocompromised	24 (46.15)	10 (55.56)	14 (41.18)	0.388	
Sepsis ^d^	19 (34.55)	4 (22.22)	15 (40.54)	0.234	
Living with HIV	16 (30.77)	9 (50.0)	7 (20.59)	0.056	
Persons Who Inject Drugs ^e^	13 (29.55)	10 (62.50)	3 (10.71)	0.001*	
Hepatitis C	9 (17.31)	7 (38.89)	2 (5.88)	0.005*	
Pneumonia	8 (15.38)	2 (11.11)	6 (17.65)	0.698	
Kidney Injury or Disease	8 (15.38)	2 (11.11)	6 (17.65)	0.698	
Renal Failure	5 (9.62)	0	5 (14.71)	0.150	
Hypertension	5 (9.62)	1 (5.56)	4 (11.76)	0.648	
Nosocomial Infection ^f^	4 (7.55)	0	4 (11.43)	0.287	

*NA* – Not applicable; *HIV* – Human immunodeficiency virus

P < 0.05 was considered statistically significant

^*^ Statistically significant observation

^a^ Section had a diverging number of data points due to missing patient information

^b^ Hospital bed capacity: Hospital 1–845 beds; Hospital 2–840 beds; Hospital 3–44 beds; Hospital 4–857 beds; Hospital 5–202 beds; Hospital 6–153 beds; Hospital 7–325 beds

^c^ Data in this sub-section was available for 52 patients (34 SAB, 18 SAIE) unless stated otherwise; deviations from this baseline are indicated per characteristic

^d-f^ Data was available for ^d^ 55 patients (37 SAB, 18 SAIE),^e^ 44 patients (28 SAB, 16 SAIE) and^f^ 53 patients (35 SAB, 18 SAIE)

**Table 2:** Logistic regression analysis of potential predictor variables in *Staphylococcus aureus* infective endocarditis development

Predictor Variable	Odds Ratio	95% Confidence Interval	P Value
Intravenous drug use	7.46	1.24–45.05	0.028*
HIV status	2.15	0.42–10.96	0.357
Hepatitis C	3.60	0.45–28.96	0.229
Intercept (Baseline Odds)	0.17	0.05–0.50	0.002*

*HIV* – Human immunodeficiency virus

P < 0.05 was considered statistically significant

^*^ Statistically significant observation

### Antimicrobial resistance profiles

Phenotypic AST data were available for all study isolates, excluding one SAB isolate, as detailed in Table 3. Benzylpenicillin [79.0% (60/76)] and TMP/SMX [51.3% (39/76)] resistance were the most common, while gentamicin and clindamycin resistance were significantly more frequent among SAB isolates [18.9% (10/53) and 20.8% (11/53), respectively] compared to SAIE isolates (0%; P < 0.05). Multidrug resistance, defined as resistance to ≥ 3 antimicrobial classes, was observed in 19.7% (15/76) of the isolates, with a higher rate in SAB [24.5% (13/53)] than in SAIE [8.7% (2/23)], though not statistically significant (Garrine et al. 2023). Molecular screening identified *mecA* in 13.0% [10/77 (9 SAB, 1 SAIE)] of isolates, largely corresponding with the AST data, except for two phenotypic MRSA isolates lacking *mecA* and one *mecA*-positive isolate that was phenotypically susceptible.

**Table 3:** Prevalence of phenotypic antimicrobial resistance among *Staphylococcus aureus* bacteraemia and infective endocarditis isolates

Antimicrobial	No. of Resistant Isolates [n = 76 (%)]	No. of Resistant Isolates by Infection Type		P Value
		Bacteraemia [n = 53 (%)] ^a^	Infective Endocarditis [n = 23 (%)]	
Benzylpenicillin	60 (78.95)	41 (77.36)	19 (82.61)	0.763
Oxacillin	8 (10.53)	6 (11.32)	2 (8.70)	1.000
Gentamicin	10 (13.16)	10 (18.87)	0	0.027*
Ciprofloxacin	11 (14.47)	8 (15.09)	3 (13.04)	1.000
Moxifloxacin	4 (5.26)	2 (3.77)	2 (8.70)	0.494
Moxifloxacin (I)	7 (9.21)	6 (11.32)	1 (4.35)	
Erythromycin	13 (17.11)	12 (22.64)	1 (4.35)	0.094
Clindamycin	11 (14.47)	11 (20.75)	0	0.028*
Tetracycline	5 (6.58)	5 (9.43)	0	0.315
Rifampicin	1 (1.32)	1 (1.89)	0	1.000
TMP/SMX	39 (51.32)	30 (56.60)	9 (39.13)	0.213
Cefoxitin screen positive	8 (10.53)	6 (11.32)	2 (8.70)	1.000
ICR positive	6 (7.89)	6 (11.32)	0	0.169

*(I)* – Intermediate resistance; *ICR* – Inducible clindamycin resistance; *TMP/SMX* – Trimethoprim/sulfamethoxazole

P < 0.05 was considered statistically significant

^*^ Statistically significant observation

^a^ One SAB isolate had no AST information available and was excluded from the analyses

*Note:* No phenotypic resistance was detected for ampicillin, streptomycin, linezolid, teicoplanin, vancomycin, tigecycline, fusidic acid or mupirocin

### Virulence gene distribution and biofilm-forming capacity

All isolates (54 SAB, 23 SAIE) were assessed for key virulence-associated genes and biofilm-forming capacity. Virulence genes were widely distributed across both infection groups, as summarised in Table 4, with most isolates harbouring 10 or more virulence genes [53.7% (29/54) SAB; 60.9% (14/23) SAIE]. However, no significant differences were observed in individual virulence gene presence, virulence gene combinations or the mean number of virulence genes per isolate (mean = 10, SD = 3.6–3.9, P > 0.05). Collectively, 89.6% (69/77) of isolates demonstrated biofilm-forming capacity (Table 5). Although strong biofilm formation was more frequent in SAIE [52.2% (12/23)] than SAB [33.3% (18/54)], the overall association between infection type and biofilm formation category was not significant (P > 0.05).

**Table 4:** Prevalence of virulence genes among *Staphylococcus aureus* bacteraemia and infective endocarditis isolates

Virulence Factor	Target Gene	No. of Isolates Positive for the Gene [n = 77 (%)]	No. of Isolates Positive for the Gene by Infection Type		P Value
			Bacteraemia [n = 54 (%)]	Infective Endocarditis [n = 23 (%)]	
MSCRAMM	*cna*	42 (54.55)	30 (55.56)	12 (52.17)	0.807
	*isdB*	77 (100)	54 (100)	23 (100)	NA
	*clfA*	56 (72.73)	41 (75.93)	15 (65.22)	0.405
	*fnbpA*	61 (79.22)	43 (79.63)	18 (78.26)	1.000
	*sdrE*	18 (23.38)	13 (24.07)	5 (21.74)	1.000
SERAM	*eap*	53 (68.83)	39 (72.22)	14 (60.87)	0.421
Regulatory factors	*icaA*	77 (100)	54 (100)	23 (100)	NA
SAgs	*tst*	3 (3.90)	2 (3.70)	1 (4.35)	1.000
	*sel-u*	19 (24.68)	13 (24.07)	6 (26.09)	1.000
	*sec*	7 (9.09)	6 (11.11)	1 (4.35)	0.667
	*seg*	19 (24.68)	13 (24.07)	6 (26.09)	1.000
	*sei*	19 (24.68)	13 (24.07)	6 (26.09)	1.000
	*SEM*	18 (23.38)	12 (22.22)	6 (26.09)	0.772
	*sen*	18 (23.38)	12 (22.22)	6 (26.09)	0.772
	*seo*	20 (25.97)	13 (24.07)	7 (30.43)	0.580
Cytolytic toxins	*pvl*	27 (35.06)	18 (33.33)	9 (39.13)	0.795
	*hlb*	40 (51.95)	27 (50.00)	13 (56.52)	0.628
Exoenzymes	*scpA*	67 (87.01)	48 (88.89)	19 (82.61)	0.474
	*sspB*	69 (89.61)	48 (88.89)	21 (91.30)	1.000
	*vWbp*	63 (81.82)	45 (83.33)	18 (78.26)	0.748

*NA* – Not applicable; *MSCRAMM* – Microbial surface components recognising adhesive matrix molecules; *SERAM* – Secretable expanded repertoire adhesive molecules; *SAgs* – Superantigens

P < 0.05 was considered statistically significant

**Table 5:** Biofilm formation capabilities of the *Staphylococcus aureus* bacteraemia and infective endocarditis isolates

Biofilm Formation Capability	No. of Isolates with Biofilm Formation Capability [n = 77 (%)]	No. of Isolates with Biofilm Formation Capability by Infection Type [n (%)]				P Value
		Infective Endocarditis (n = 23)		Bacteraemia (n = 54)		
		Mean OD (Range)	n (%)	Mean OD (Range)	n (%)	
Non	8 (10.39)	0.12 (0.04–0.19)	2 (8.70)	0.19 (0.17–0.20)	6 (11.11)	1.000
Weak	19 (24.68)	0.26 (0.22–0.31)	5 (21.74)	0.26 (0.21–0.31)	14 (25.93)	0.780
Moderate	20 (25.97)	0.36 (0.34–0.41)	4 (17.39)	0.41 (0.34–0.49)	16 (29.63)	0.395
Strong	30 (38.96)	1.03 (0.52–2.40)	12 (52.17)	0.85 (0.53–1.36)	18 (33.33)	0.134

*OD* – Optical density

P < 0.05 was considered statistically significant

### Phylogenetic clustering

**Fig. 1:**
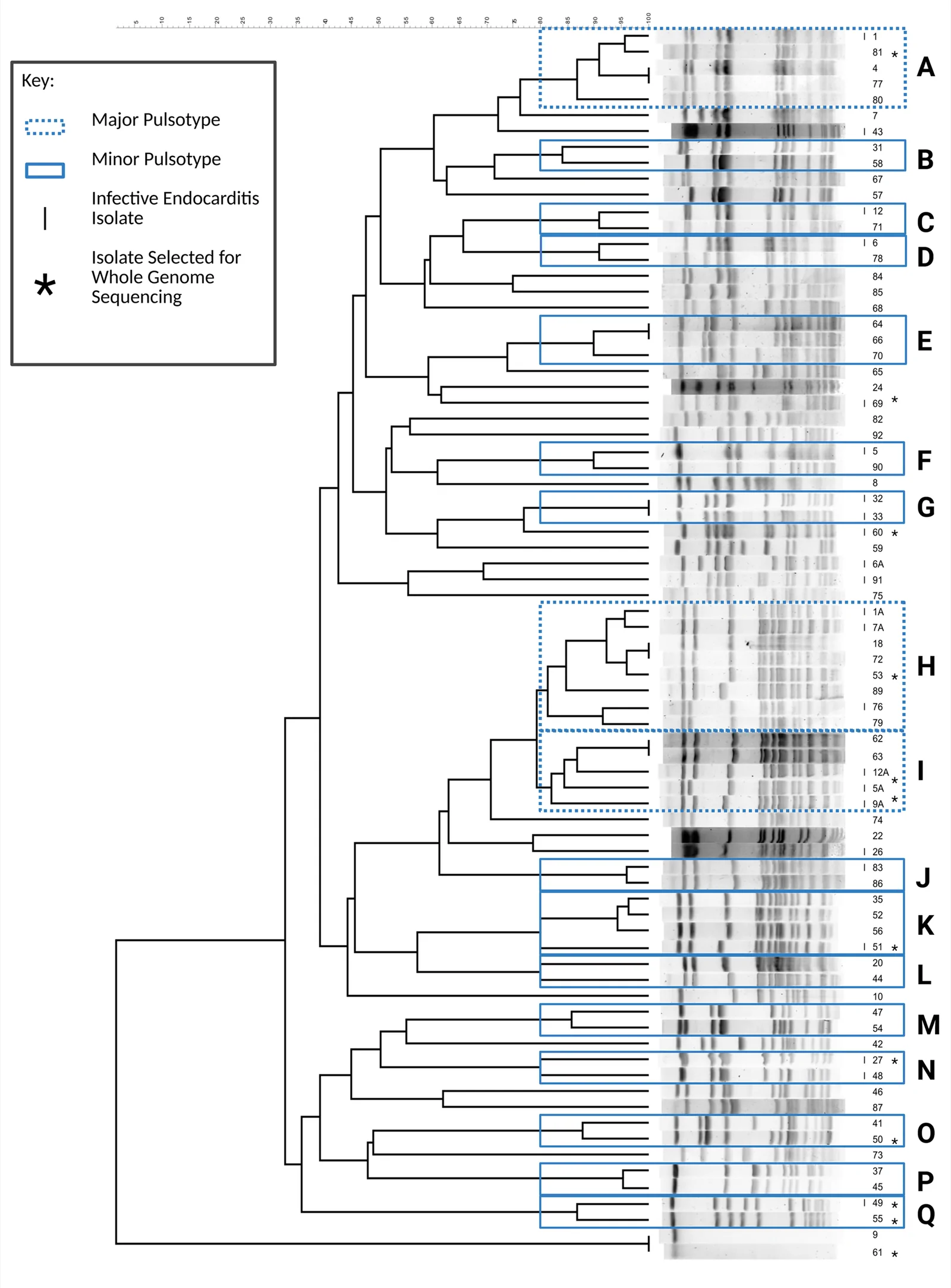
Dendrogram illustrating the genetic relatedness of the *Staphylococcus aureus* bacteraemia and infective endocarditis isolates at a similarity cut-off value of ≥ 80

Three major pulsotypes, 15 minor pulsotypes, 26 singletons and two untypable SAB isolates were identified (Fig. 1). Significant overall associations were found between specific pulsotypes and several genes [*mecA*, *cna*, *eap*, *egc*, *hlb*, *pvl*, *sdre* (P < 0.05 to P < 0.001)], as well as between pulsotypes and AMR [ciprofloxacin, clindamycin, erythromycin, gentamicin, oxacillin, tetracycline, moxifloxacin (P < 0.05)]. Notably, the *mecA*-positive pulsotype K isolates demonstrated extensive MDR to the above-mentioned antimicrobials, while pulsotypes E, H and I were exclusively *pvl*-positive. A comprehensive mapping of phenotypic and genotypic characteristics by pulsotype cluster is provided in Table S2 (Online Resource 1).

### Genomic characteristics of representative isolates

**Fig. 2:**
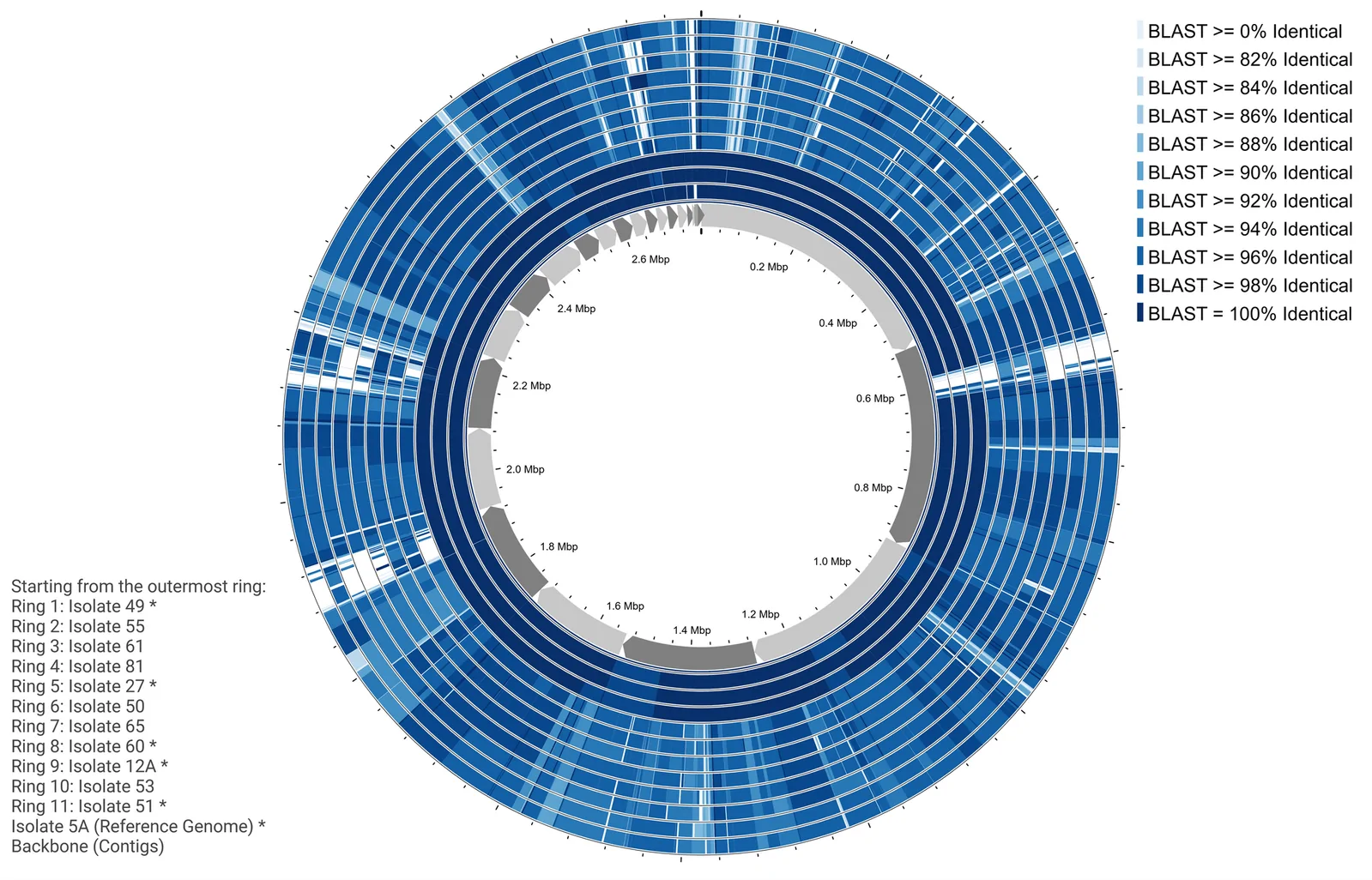
Illustration of genomic similarity between the representative *Staphylococcus aureus* bacteraemia and infective endocarditis isolates. Asterisks (*) denote *Staphylococcus aureus* infective endocarditis isolates. Figure created with Proksee.ca (Grant et al. 2023)

The 12 representative *S. aureus* isolates revealed diverse genomic characteristics. Four isolates [SA51, SA53, SA5A and SA12A (1 SAB, 3 SAIE)] demonstrated high genomic similarity, with a mean SNP difference of 151. A genomic comparison of the 12 representative isolates, generated using Proksee (Grant et al. 2023), is shown in Fig. 2. Strain typing revealed 11 distinct staphylococcal protein A (*spa*) types and eight different sequence types (STs) across seven clonal complexes (CCs). The most common *spa* type was t355 [16.7% (2/12); both SAIE], while MLST ST152 was predominant [33.3% (4/12); 1 SAB, 3 SAIE]. Among all representative isolates, 66.7% (8/12) belonged to just three CCs (CC152, CC45 and CC8). Two MRSA SAB isolates carried staphylococcal cassette chromosome *mec* (SCC*mec*) types III (3A) and IVd (2B), with variable carriage of other MGEs observed. The overall WGS findings, including mobilome, resistome and virulome profiles, are summarised in Table S3 (Online Resource 1), while the strain-typing results for each representative isolate are presented in Table S4 (Online Resource 1).

**Fig. 3:**
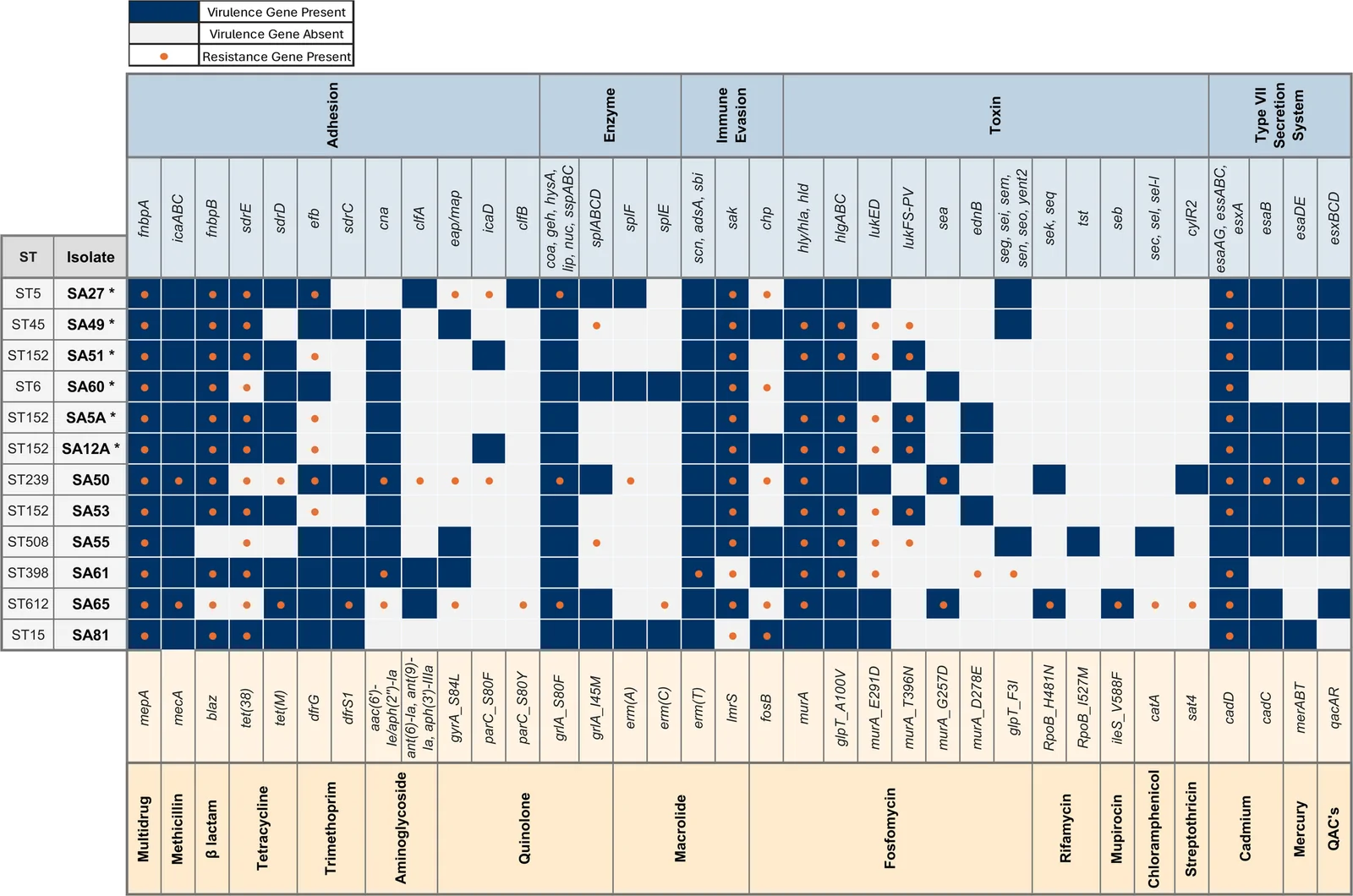
Genomic characterisation of resistance and virulence determinants in the representative *Staphylococcus aureus* isolates, including six bacteraemia isolates and six infective endocarditis isolates

The WGS AMR profiles broadly aligned with the phenotypic AST results. The multidrug efflux transporter (*mepA*) and tetracycline efflux major facilitator superfamily (MFS) transporter [*tet*(38)] were detected in 100% of the isolates, while penicillin-hydrolysing class A β-lactamase (*blaZ*) and penicillinase repressor (*blaI*) were present in 91.7% [11/12 (5 SAB, 6 SAIE)]. The gentamicin resistance *aac*(6’)-*Ie/aph*(2″)-*Ia* gene, as well as the macrolide, lincosamide and streptogramin B (MLSB) cross-resistance genes [*erm*(ACT)], were observed exclusively in SAB isolates [50% (3/6)]. Adhesion genes crucial for colonisation and biofilm formation were widely detected, with the autolysin (*atl*), elastin-binding protein (*ebp*), *fnbpA*, *icaABCR* and *spa* genes present in 100% of isolates. Enzymatic virulence genes were extensively represented with 100% of isolates harbouring glycerol ester hydrolase (*geh*), hyaluronate lyase (*hysA*), lipase (*lip*), *nuc*, serine V8 protease (*sspABC*), Coa (*coa*) gene and zinc metalloproteinase aureolysin (*aur*). The toxin virulence gene profiles revealed diverse representation across isolates. Notably, the alpha-haemolysin (*hly*/*hla*), delta haemolysin (*hld*) and bi-component gamma-haemolysin (Hlg) subunits A, B and C (*hlgABC*) genes were present in 100% of the isolates, while the PVL bi-component subunit F and S [*lukFS*-*PV* (1 SAB, 3 SAIE)] were present in 33.3% (4/12) of the isolates. All isolates harboured the type VII secretion system (T7SS) accessory factor A gene (*esaAG*), *essABC* and *esxA*. The WGS AMR and virulence profiles of each representative SAB and SAIE isolate are illustrated in Fig. 3.

## Discussion

Infective endocarditis is considered a rare manifestation of SAB; however, the high SAIE prevalence in this study (29.9%) exceeded global estimates ranging from 6% in Asia to 27% in Switzerland (John et al. 2024; Ngiam et al. 2024; Papadimitriou-Olivgeris et al. 2024). Public hospitals in Gauteng predominantly care for socioeconomically disadvantaged patients with higher rates of immunodeficiency, comorbidities and IVDU, factors that may have contributed to the overrepresentation of SAIE (Stats South Africa 2023). The marked male predominance (95.7%) and younger age distribution (25–44 years) observed in this study likely reflect the broader South African risk-factor profile, where poverty, limited healthcare access, HIV, tuberculosis, prior rheumatic heart disease and IVDU often disproportionately affect males in LMICs and drive earlier disease onset (de Villiers 2021; Pecoraro et al. 2021). In contrast, SAIE trends in HICs are more strongly shaped by ageing populations with higher burdens of degenerative valvular disease and HA risk factors (Cahill et al. 2017). The high proportion of PWID-associated cases in this cohort (29.5%), relative to the 9.5% reported in a recent Gauteng study (John et al. 2024), may partly reflect the strong PWID-SAIE link (P < 0.001) driven by the introduction of pathogens and endocardial-damaging particulate matter through non-sterile injection practices (Masters et al. 2025).

The distribution of *S. aureus* isolates across hospitals and wards further highlighted potential epidemiological patterns. Nearly half (49.4%) of the *S. aureus* isolates were classified as community-associated (CA) cases, inferred from both their clinical presentations in emergency wards and corroborating genotypic data (Table S4 in Online Resource 1). Specifically, within the WGS representative subset, all seven emergency ward isolates belonged to classic CA lineages (ST152, ST5, ST6 and ST45), whereas characteristic HA lineages (ST239, ST612, ST15 and ST508) were recovered exclusively from non-emergency wards. The concentration of SAIE and PWID cases in emergency wards (P < 0.001) may reflect delayed care-seeking among PWIDs due to stigma, limited healthcare access or concerns regarding legal and social repercussions (Masters et al. 2025). Conversely, SAB occurred significantly more often in high-dependency wards (P < 0.05), consistent with the elevated infection risk associated with critical illness, invasive procedures and extended hospitalisation (Samuel et al. 2023).

Overall, no distinct SAIE-associated resistance profile was evident from the AST patterns, with significant differences limited to gentamicin and clindamycin resistance, both of which were confined to SAB isolates (P < 0.05). The absence of gentamicin and clindamycin resistance among SAIE isolates may further reflect their predominantly CA origin and limited prior antimicrobial exposure, particularly given the relatively restricted clinical use of both agents. Gentamicin is largely avoided outside of selected prosthetic-valve IE cases, whereas clindamycin is generally reserved for severe staphylococcal infections in penicillin-allergic patients or IE prophylaxis before dental procedures (Habib et al. 2015; National Department of Health 2019). Few studies have examined the AMR profiles of SAIE isolates; however, resistance among SAIE isolates in the present study was generally lower than previously reported in India, another LMIC (Gupta et al. 2021). Speculatively, the lower overall resistance observed in SAIE isolates might reflect the fitness costs associated with maintaining broad-spectrum AMR mechanisms, although further research is needed to clarify this interpretation (Beceiro et al. 2013).

Both SAB and SAIE isolates displayed similarly robust virulence potential. The high prevalence of adhesion- and exoenzyme-associated genes was expected, given their importance in promoting the establishment and progression of invasive *S. aureus* infections (Kinney et al. 2022a, 2022b). Whole-genome sequencing provided additional virulence resolution, with exotoxin, haemolysin and *ica*ABC gene profiles supporting shared virulence potential across both infection groups. Compared with the more variable carriage observed among representative SAB isolates, nearly all representative SAIE isolates harboured the complete 12-gene T7SS repertoire, suggesting a potential role for T7SS-mediated colonisation, immune evasion and interbacterial competition in SAIE (Boardman et al. 2023). Although SAIE isolates generally produced more robust biofilms, the lack of correlation between biofilm strength and infection type supports evidence that biofilm formation alone cannot predict IE development (Bouchiat et al. 2015). Thus, the comparable virulence and biofilm findings across both infection types align with reports from France and other HICs, supporting the view that severe invasive potential may be broadly shared among *S. aureus* strains rather than restricted to a distinct SAIE-associated profile (Tristan et al. 2012; Bastien et al. 2023). *Staphylococcus aureu*s IE risk is therefore unlikely to be driven by a single virulence marker but may instead reflect multiple bacterial and host-related factors converging on an increased propensity to cause IE.

Genotyping revealed considerable genetic diversity across both SAB and SAIE isolates, indicating that infections arose from diverse genetic backgrounds rather than a single clonal lineage, a pattern supported by WGS and consistent with previous studies (Bouchiat et al. 2015; Lilje et al. 2017). Concerningly, closely related isolates identified among PWIDs were linked to the endemic PVL-positive ST152/CC152 lineage, which was previously implicated in outbreaks among South African gold miners in 2017 (Ismail et al. 2020). This major African CA-MSSA lineage was highly prevalent among SAIE isolates in the present study, likely reflecting the underlying regional CA population structure rather than an exclusive SAIE-specific advantage (Lawal et al. 2022). However, the consistent PVL-positive nature of ST152/CC152 may still contribute to the capacity of this lineage to establish severe invasive infections in high-risk patient groups (Lawal et al. 2022).

The lineage distribution observed in the present study differed from patterns typically reported in Western European SAIE populations, with CC8 and CC15 restricted to SAB isolates, whereas CC5 and CC45 showed only limited representation among SAIE isolates (Tristan et al. 2012; Bouchiat et al. 2015). Representative WGS SAB isolates included endemic South African HA-MRSA clones such as t037-ST239-MRSA-III (CC8) and ST612-MRSA-IV (CC8), with the wider variety of MGEs likely due to HA-acquisition (Singh-Moodley et al. 2020; Samuel et al. 2023; Hetsa et al. 2024). Together, these findings suggest that regional epidemiological differences strongly influence circulating CA-SAIE lineages, whereas SAB isolates may more closely reflect the local HA reservoir. The presence of genetically related ST152-t355 isolates from both SAB and SAIE cases (SA5A and SA51) suggests that closely related strains may be associated with either clinical syndrome under favourable host or clinical conditions. However, larger WGS-based studies are needed to confirm this observation in South African healthcare settings.

This study provides a comprehensive comparison of the phenotypic, genotypic and demographic characteristics of SAB and SAIE isolates from public healthcare settings in South Africa. However, the retrospective, observational design of this study posed inherent challenges. The modest sample size and uneven distribution of infection types (SAB to SAIE) likely reduced statistical power to detect smaller effects, while limited or absent clinical information introduced a potential risk of infection type misclassification. Nonetheless, these limitations reflect the real-world data collection constraints typical of LMIC public health sector research. Future studies should explore the relationship between patient characteristics, *S. aureus* infection dynamics and the progression from SAB to SAIE in LMICs.

## Conclusion

The study provided valuable insights into the AMR and pathogenic profiles of invasive *S. aureus* isolates from public healthcare settings in Gauteng, South Africa. No distinct SAIE-specific phenotypic or genotypic markers were identified, although resistance to gentamicin and clindamycin was significantly associated with SAB isolates. The overall similarity between SAB and SAIE isolates suggests that the biological capacity to cause IE may not be restricted to a distinct SAIE-specific lineage or virulence profile but may instead depend on the combined effects of bacterial traits, host susceptibility and exposure-related risk factors, particularly among high-risk groups such as PWIDs. The high prevalence of PWID-associated SAIE observed underscores the need for targeted public health interventions and holistic strategies to address modifiable risk factors in both SAB and SAIE management.

## Supplementary Information

Below is the link to the electronic supplementary material.

Supplementary file1 (ZIP 25298 KB)

## Data Availability

The whole-genome sequencing datasets generated and analysed during this study have been deposited in the DDBJ/ENA/GenBank under BioProject number PRJNA1366126, with accession numbers SAMN53307515-SAMN53307526. All other datasets used and analysed during the current study are available from the corresponding author on reasonable request.
