# Supplementary material for: Genomic comparison of Staphylococcus aureus isolates from patients with bacteraemia and infective endocarditis at public hospitals in Gauteng, South Africa
Source: World J Microbiol Biotechnol. 2026 Jul 27;42(8):430. doi: 10.1007/s11274-026-05145-z (PMC13407745; doi:10.1007/s11274-026-05145-z)
Supplement: Supplementary file 1 — Supplementary file1 (ZIP 25298 KB) [file 11274_2026_5145_MOESM1_ESM.zip › Supplementary Material.docx]

**Supplementary material for: Genomic comparison of S*taphylococcus aureus* isolates from patients with bacteraemia and infective endocarditis at public hospitals in Gauteng, South Africa**

**Lz D Jansen van Vuuren^a*^**, Thabo Hamiwe^a^, Veronica Ueckermann^b^, Marleen M Kock^a c^, Anel Bosch^a c^, Marthie M Ehlers^a c*^

^a^ Department of Medical Microbiology, University of Pretoria, Gauteng, South Africa

^b^ Division for Infectious Diseases, Department of Internal Medicine, Steve Biko Academic Hospital and University of Pretoria, Pretoria, South Africa

^c^ Department of Medical Microbiology, Tshwane Academic Division, National Health Laboratory Service, Pretoria, South Africa

* Corresponding authors: Lz D Jansen van Vuuren (Lz.spaltman@tuks.co.za), Marthie M Ehlers (marthie.ehlers@up.ac.za)

**Table S1: Multiplex polymerase chain reaction assay primer sequences and** **amplification conditions for the species identification, confirmation and detection of virulence genes harboured by *Staphylococcus aureus* isolates**

|  | **Target Gene** | **Primer Sequence (5’ to 3’)** | **Size (bp)** | **Ref** | **Amplification Conditions ^a^** |
| --- | --- | --- | --- | --- | --- |
| **Identification** | 16S rRNA | F-TGAGATGTTGGGTTAAGTCCCGCA  R-CGGTTTCGCTGCCCTTTGTATTGT | 597 | Al-Talib et al., 2009 | 95°C for 15 min (1 cycle); 94°C for 30 sec, 58°C for 3 min, 72°C for 1 min and 30 sec (30 cycles); 72°C for 2 min |
|  | *nuc* | F-TCGCTTGCTATGATTGTGG  R-GCCAATGTTCTACCATAGC | 359 | Hirotaki et al., 2011 |  |
|  | *mecA* | F-AAAATCGATGGTAAAGGTTGGC  R-AGTTCTGCAGTACCGGATTTGC | 310 | Parvin et al., 2021 |  |
|  | *S. epidermidis* fragment | F-ATCAAAAAGTTGGCGAACCTTTTCA  R-CAAAAGAGCGTGGAGAAAAGTATCA | 124 | Martineau et al., 2000 |  |
| **MSCRAMM** | *isdB* | F-GGCGTTGCATCTGTAGCAATT  R-TGGACTTGCAACTGCTTCAGTT | 150 | Palazzolo-Ballance et al., 2008 | 95°C for 15 min (1 cycle); 94°C for 30 sec, 50°C to 58°C for 3 min, 72°C for 1 min and 30 sec (33 cycles); 72°C for 5 min |
|  | *cna* | F-AGTGGTTACTAATACTG  R-CAGGATAGATTGGTTTA | Variable  (560 repeats) | Goudarzi et al., 2017; Peacock et al., 2002 |  |
|  | *clfA* | F-GTAGGTACGTTAATCGGTT  R-CTCATCAGGTTGTTCAGG | 1 584 |  |  |
|  | *fnbpA* | F-CACAACCAGCAAATATAG  R-CTGTGTGGTAATCAATGTC | 1 362 |  |  |
|  | *sdrE* | F-CAGTAAATGTGTCAAAAGA  R-TTGACTACCAGCTATATC | 767 |  |  |
| **SERAM** | *eap* | F-TAACATTTAATAAGAATCAA  R-CCATTTACTGCAATTGT | 940 |  |  |
| **Regulatory Factors** | *icaA* | F-ACACTTGCTGGCGCAGTCAA  R-TCTGGAACCAACATCCAACA | 188 | Parastan et al., 2020 |  |
| **SAgs** | *tst* | F-GCTTGCGACAACTGCTACAG  R-TGGATCCGTCATTCATTGTTAT | 180 | Monday and Bohach, 1999 |  |
|  | *sel-u* | F-GCAGCTTACTATTTATGTTAAATGGC  R-CTATTTGATTTCCATCATGCTCGG | 390 | Vu et al., 2014 |  |
| **SAgs** | *sec* | F-GTAAAGTTACAGGTGGCAAAACTTG  R-CATATCATACCAAAAAGTATTGCCGT | 297 | Jarraud et al., 2002 |  |
|  | *seg* | F-AATTATGTGAATGCTCAACCCGATC  R-AAACTTATATGGAACAAAAGGTACTAGTTC | 642 |  |  |
|  | *sei* | F-CTCAAGGTGATATTGGTGTAGG  R-AAAAAACTTACAGGCAGTCCATCTC | 576 |  |  |
|  | *sem* | F-CTATTAATCTTTGGGTTAATGGAGAAC  R-TTCAGTTTCGACAGTTTTGTTGTCAT | 300 |  |  |
|  | *sen* | F-ATGAGATTGTTCTACATAGCTGCAAT  R-AACTCTGCTCCCACTGAAC | 680 |  |  |
|  | *seo* | F-AGTTTGTGTAAGAAGTCAAGTGTAGA  R-ATCTTTAAATTCAGCAGATATTCCATCTAAC | 180 |  |  |
| **Cytolytic Toxins** | *pvl* | F-ATCATTAGGTAAAATGTCTGGACATGATCCA  R-GCATCAACTGTATTGGATAGCAAAAGC | 433 |  |  |
|  | *hlb* | F-GTGCACTTACTGACAATAGTGC  R-GTTGATGAGTAGCTACCTTCAGT | 309 |  |  |
| **Exoenzyme** | *scpA* | F-AGGAGTTTTTATATGAAAAG  R-TACCTTTCTAAAATACAAAT | 1 235 | Zdzalik et al., 2012 |  |
|  | *sspB* | F-TTTGATTTAAATGGAGGGTA  R-CCTGCCTTTAATCAATATCT | 1 236 |  |  |
|  | *vWbp* | F-GTGGTTTCTGGGGAGAAGAAT  R-TTATTTGCCATTATATACTTTATT | 1 449 | Chmagh and Abd Al-Abbas, 2019 |  |

**Abbreviations:** *bp* – Base pairs; *F* – Forward primer; *R* – Reverse primer; *MSCRAMM* – Microbial surface components recognising adhesive matrix molecules; *SERAM* – Secretable expanded repertoire adhesive molecules;  *SAgs* – Superantigens

**^a^** **Amplification conditions**: Initial Denaturation; Denaturation, Annealing, Initial Extension; Final Extension

**Table S2: Phenotypic and genotypic profiling insights into identified *Staphylococcus aureus* pulsed-field gel electrophoresis pulsotypes**

| **Pulsotype** | **Cluster** | **Dominant Resistance Profile (Phenotypic)** | **Associated Target Genes  (Genotypic)** | **Total No. of Isolates in Cluster  [n = 77 (%)]** | **No. of Isolates in Cluster by Infection Type [n (%)]** | |
| --- | --- | --- | --- | --- | --- | --- |
|  |  |  |  |  | **Bacteraemia [n=54 (%)]** | **Infective Endocarditis  [n= 23 (%)]** |
| **Major** | H | Variable | *cna* ^(+)^, *hlb* ^(+)^, *pvl* ^(+)***^, *eap* ^(-)***^ | 8 (10.4) | 5 (9.3) | 3 (13.0) |
|  | A | Variable | *cna* ^(-)^, *hlb* ^(-)**^ | 5 (6.5) | 4 (7.4) | 1 (4.3) |
|  | I | Variable | *pvl* ^(+)^, *eap* ^(-)^ | 5 (6.5) | 2 (3.7) | 3 (13.0) |
| **Minor** | E | Variable | *pvl* ^(+)^, *eap* ^(-)^ | 4 (5.2) | 3 (5.6) | 1 (4.3) |
|  | K | MDR ^(+)**^, Oxacillin ^(+)**^, Gentamicin ^(+)**^, Ciprofloxacin ^(+)**^, Erythromycin ^(+)**^, Clindamycin ^(+)**^, ICR^(+)**^, Tetracycline ^(+)**^, Moxifloxacin ^(+)**^, Cefoxitin screen ^(+)**^ | *mecA* ^(+)***^ | 3 (3.9) | 3 (5.6) | 0 |
|  | B | Variable | Variable | 2 (2.6) | 2 (3.7) | 0 |
|  | C | Variable | *seg* ^(+)^, *sei* ^(+)^, *sem* ^(+)^, *sen* ^(+)^, *seo* ^(+)^, *selu* ^(+)^ | 2 (2.6) | 1 (1.9) | 1 (4.3) |
|  | D | Variable | Variable | 2 (2.6) | 1 (1.9) | 1 (4.3) |
|  | F | Variable | *seg* ^(+)^, *sei* ^(+)^, *sem* ^(+)^, *sen* ^(+)^, *seo* ^(+)^, *selu* ^(+)^ | 2 (2.6) | 1 (1.9) | 1 (4.3) |
|  | G | Variable | Variable | 2 (2.6) | 0 | 2 (8.7) |
|  | J | Variable | Variable | 2 (2.6) | 1 (1.9) | 1 (4.3) |
|  | L | Variable | Variable | 2 (2.6) | 2 (3.7) | 0 |
|  | M | Variable | Variable | 2 (2.6) | 2 (3.7) | 0 |
|  | N | Moxifloxacin ^(+)^ | *sdre* ^(+)^, *seg* ^(+)^, *sei* ^(+)^, *sem* ^(+)^, *sen* ^(+)^, *seo* ^(+)^, *selu* ^(+)^ | 2 (2.6) | 0 | 2 (8.7) |
|  | O | Variable | *mecA* ^(+)^ | 2 (2.6) | 2 (3.7) | 0 |
|  | P | Variable | *sdre* ^(+)^, *seg* ^(+)^, *sei* ^(+)^, *sem* ^(+)^, *sen* ^(+)^, *seo* ^(+)^, *selu* ^(+)^ | 2 (2.6) | 2 (3.7) | 0 |
|  | Q | Variable | *sdre* ^(+)^, *seg* ^(+)^, *sei* ^(+)^, *sem* ^(+)^, *sen* ^(+)^, *seo* ^(+)^, *selu* ^(+)^ | 2 (2.6) | 1 (1.9) | 1 (4.3) |
| **Unclustered** | Singletons | Variable | Variable | 26 (33.8) | 20 (37.0) | 6 (26.1) |
|  | Untypable | Variable | Variable | 2 (2.6) | 2 (3.7) | 0 |

**Abbreviations:** *MDR* – Multidrug-resistance; *ICR* – Inducible clindamycin resistance

***Note*:** Descriptive percentages for overall cluster prevalence and infection types were calculated from the total study population (N = 77). However, to accurately evaluate specific genotypic
 and phenotypic cluster associations, inferential statistical analyses were strictly limited to clustered isolates (n = 49 across 17 distinct clusters).

P < 0.05 was considered a statistically significant cluster association (default baseline).

****** P < 0.01 was considered a highly statistically significant association.

******* P < 0.001 was considered an extremely statistically significant association.

**^(+)^** Indicates a cluster-specific enrichment (disproportionate presence) of the gene or resistance profile compared to all other clusters combined.

**^(-)^** Indicates a cluster-specific depletion (complete absence or disproportionate lack) of the gene compared to all other clusters combined.

**Table S3: Whole genome sequencing insights into representative *Staphylococcus aureus* bacteraemia and infective endocarditis isolates**

| **Characterisation Category** | **Type** | **Subtype or** **Target Gene  (Each Identified Individually)** | **No. of Isolates Positive for Each Genetic Element [n = 12 (%)]** | **No. of Isolates Positive for the Genetic Element by Infection Type [n (%)]** | |
| --- | --- | --- | --- | --- | --- |
|  |  |  |  | **Infective Endocarditis (n = 6)** | **Bacteraemia (n = 6)** |
| Strain Typing | Staphylococcal Protein A | t355 | 2 (16.67) | 2 (33.33) | 0 |
|  |  | t1299, t9228, t630, t701 | 1 (8.33) | 1 (16.67) | 0 |
|  |  | t37, t1828, t445, t1451, t190, t346 | 1 (8.33) | 0 | 1 (16.67) |
|  | Clonal Complex | CC152 | 4 (33.33) | 3 (50.00) | 1 (16.67) |
|  |  | CC8 | 2 (16.67) | 0 | 2 (33.33) |
|  |  | CC45 | 2 (16.67) | 1 (16.67) | 1 (16.67) |
|  |  | CC5, CC6 | 1 (8.33) | 1 (16.67) | 0 |
|  |  | CC15, CC398 | 1 (8.33) | 0 | 1 (16.67) |
|  | Sequence Type | ST152 | 4 (33.33) | 3 (50.00) | 1 (16.67) |
|  |  | ST5, ST45, ST6 | 1 (8.33) | 1 (16.67) | 0 |
|  |  | ST239, ST508, ST398, ST612, ST15 | 1 (8.33) | 0 | 1 (16.67) |
| Mobile Genetic Elements | SCC*mec* | III (3A), IVd (2B) | 1 (8.33) | 0 | 1 (16.67) |
|  | Plasmid Incompatibility Type and Plasmid Replication Protein Gene | Inc18/Rep3, *rep*5a_1, *rep*16_3 | 7 (58.33) | 5 (83.33) | 2 (33.33) |
|  |  | RepA_N, | 2 (16.67) | 1 (16.67) | 1 (16.67) |
|  |  | Rep_trans, Rep1, *rep*20_3 | 2 (16.67) | 0 | 2 (33.33) |
|  |  | Rep1/RepA_N, Rep3, RepL, *rep*21_20, *rep*7a_22, *rep*5c_1, *rep*13_1, *rep*21_22, *rep*7c_1, *rep*10_4 | 1 (8.33) | 0 | 1 (16.67) |
|  |  | *rep*20_7 | 1 (8.33) | 1 (16.67) | 0 |
|  | Insertion Sequences | IS*Sau3* | 3 (25.00) | 2 (33.33) | 1 (16.67) |
|  |  | IS*Sau6*, IS*Lgar5* | 2 (16.67) | 0 | 2 (33.33) |
|  |  | IS*Sau8*, IS*Sep3* | 1 (8.33) | 0 | 1 (16.67) |
|  | Composite Transposons | CN_2257_IS*Sau6*, CN_12274 IS*Sau6* | 1 (8.33) | 0 | 1 (16.67) |
| Antimicrobial Resistance | Multidrug | *mepA* | 12 (100) | 6 (100) | 6 (100) |
|  | Tetracycline | *tet*(38) | 12 (100) | 6 (100) | 6 (100) |
|  |  | *tet*(M) | 2 (16.67) | 0 | 2 (33.33) |
|  | Beta-lactam | *blaZ*, *blaI* | 11 (91.67) | 6 (100) | 5 (83.33) |
|  |  | *blaR1* | 10 (83.33) | 6 (100) | 4 (66.67) |
|  |  | *blaPC1*, *mecI*, | 1 (8.33) | 0 | 1 (16.67) |
|  | Fosfomycin | *murA* | 9 (75.00) | 4 (66.67) | 5 (83.33) |
|  |  | *glpT*_A100V | 7 (58.33) | 4 (66.67) | 3 (50.00) |
|  |  | *murA*_E291D | 7 (58.33) | 4 (66.67) | 3 (50.00) |
|  |  | *murA*_T396N | 6 (50.00) | 4 (66.67) | 2 (33.33) |

**Table S3: Whole genome sequencing insights into representative *Staphylococcus aureus* bacteraemia and infective endocarditis isolates (*continued*)**

| **Characterisation Category** | **Type** | | **Subtype or** **Target Gene  (Each Identified Individually)** | **No. of Isolates Positive for Each Genetic Element [n = 12 (%)]** | **No. of Isolates Positive for the Genetic Element by Infection Type [n (%)]** | |
| --- | --- | --- | --- | --- | --- | --- |
|  |  |  |  |  | **Infective Endocarditis (n = 6)** | **Bacteraemia (n = 6)** |
| Antimicrobial Resistance | Fosfomycin | *fosB* | | 5 (41.67) | 2 (33.33) | 3 (50.00) |
|  |  | *murA*_G257D | | 2 (16.67) | 0 | 2 (33.33) |
|  |  | *murA*_D278E, *glpT*_F3I | | 1 (8.33) | 0 | 1 (16.67) |
|  | Trimethoprim | *dfrG* | | 6 (50.00) | 4 (66.67) | 2 (33.33) |
|  |  | *dfrS1* | | 1 (8.33) | 0 | 1 (16.67) |
|  | Aminoglycoside | *aac*(6')-*Ie/aph*(2'')-*Ia* | | 3 (25.00) | 0 | 3 (50.00) |
|  |  | *ant*(6)-*Ia*, *ant*(9)-*Ia*, *aph*(3')*-IIIa* | | 1 (8.33) | 0 | 1 (16.67) |
|  | Quinolone | *gyrA*_S84L, *grlA*_S80F | | 3 (25.00) | 1 (16.67) | 2 (33.33) |
|  |  | *parC*_S80F, *grlA*_I45M | | 2 (16.67) | 1 (16.67) | 1 (16.67) |
|  |  | *parC*_S80Y | | 1 (8.33) | 0 | 1 (16.67) |
|  | Methicillin | *mecA*, *mecR1* | | 2 (16.67) | 0 | 2 (33.33) |
|  | Macrolide | *Erm*(ACT*)* | | 1 (8.33) | 0 | 1 (16.67) |
|  | Rifamycin, Chloramphenicol, Mupirocin, Streptothricin | *RpoB*_H481N, *RpoB*_I527M, *catA*, *ileS*_V588F, *sat4* | | 1 (8.33) | 0 | 1 (16.67) |
| Stress Response | Macrolide and Phenicol | *lmrS* | | 12 (100.00) | 6 (100.00) | 6 (100.00) |
|  | Cadmium | *cadD* | | 11 (91.67) | 6 (100.00) | 5 (83.33) |
|  | Cadmium/Lead/Zinc, Mercury, Quaternary Ammonium Compounds | *cadC*, *merA*, *merB*, *merT*, *qacA*, *qacR* | | 1 (8.33) | 0 | 1 (16.67) |
| Virulence | Adhesion | *atl*, *ebp*, *fnbpA*, *icaABCR*, *spa* | | 12 (100.00) | 6 (100.00) | 6 (100.00) |
|  |  | *ebh* | | 11 (91.67) | 6 (100.00) | 5 (83.33) |
|  |  | *fnbpB* | | 10 (83.33) | 6 (100.00) | 4 (66.67) |
|  |  | *sdrE* | | 10 (83.33) | 5 (83.33) | 5 (83.33) |
|  |  | *sdrD* | | 9 (75.00) | 5 (83.33) | 4 (66.67) |
|  |  | *efb* | | 8 (66.67) | 3 (50.00) | 5 (83.33) |
|  |  | *sdrC* | | 6 (50.00) | 1 (16.67) | 5 (83.33) |
|  |  | *cna* | | 4 (33.33) | 3 (50.00) | 1 (16.67) |
|  |  | *clfA*, *eap/map* | | 3 (25.00) | 1 (16.67) | 2 (33.33) |
|  |  | *icaD* | | 2 (16.67) | 2 (33.33) | 0 |
|  |  | *clfB* | | 1 (8.33) | 1 (16.67) | 0 |

**Table S3: Whole genome sequencing insights into representative *Staphylococcus aureus* bacteraemia and infective endocarditis isolates (*continued*)**

| **Characterisation Category** | **Type** | | **Subtype or** **Target Gene  (Each Identified Individually)** | **No. of Isolates Positive for Each Genetic Element [n = 12 (%)]** | **No. of Isolates Positive for the Genetic Element by Infection Type [n (%)]** | |
| --- | --- | --- | --- | --- | --- | --- |
|  |  |  |  |  | **Infective Endocarditis (n = 6)** | **Bacteraemia (n = 6)** |
| Virulence | Enzyme and Immune Evasion | *geh*, *hysA*, *lip*, *nuc*, *sspA*, *sspB*, *sspC*, *coa*, *aur*, *scn*, *adsA*, *sbi* | | 12 (100.00) | 6 (100.00) | 6 (100.00) |
|  |  | *sak* | | 10 (83.33) | 6 (100.00) | 4 (66.67) |
|  |  | *splA*, *splB*, *splC*, *splD*, *chp* | | 5 (41.67) | 2 (33.33) | 3 (50.00) |
|  |  | *splF* | | 3 (25.00) | 2 (33.33) | 1 (16.67) |
|  |  | *splE* | | 2 (16.67) | 1 (16.67) | 1 (16.67) |
|  | Toxin | *hly/hla*, *hld*, *hlgABC* | | 12 (100.00) | 6 (100.00) | 6 (100.00) |
|  |  | *set34* | | 9 (75.00) | 5 (83.33) | 4 (66.67) |
|  |  | *set7*, *set16* | | 8 (66.67) | 5 (83.33) | 3 (50.00) |
|  |  | *set15* | | 8 (66.67) | 6 (100.00) | 2 (33.33) |
|  |  | *set4* | | 7 (58.33) | 4 (66.67) | 3 (50.00) |
|  |  | *set18* | | 6 (50.00) | 4 (66.67) | 2 (33.33) |
|  |  | *set36* | | 6 (50.00) | 3 (50.00) | 3 (50.00) |
|  |  | *sel26* | | 5 (41.67) | 1 (16.67) | 4 (66.67) |
|  |  | *lukE*, *lukD*, *set37*, *sel-k* | | 5 (41.67) | 2 (33.33) | 3 (50.00) |
|  |  | *lukF-PV*, *lukS-PV* | | 4 (33.33) | 3 (50.00) | 1 (16.67) |
|  |  | *set39*, *selX* | | 4 (33.33) | 2 (33.33) | 2 (33.33) |
|  |  | *sea*, *set1*, *set2*, *set5* | | 3 (25.00) | 1 (16.67) | 2 (33.33) |
|  |  | *ednB*, *seg*, *sei*, *sem*, *sen*, *seo*, *yent2* | | 3 (25.00) | 2 (33.33) | 1 (16.67) |
|  |  | *set3*, *set6*, *set8*, *set19*, *set22*, *set25* | | 2 (16.67) | 1 (16.67) | 1 (16.67) |
|  |  | *sek*, *set21*, *set26*, *set30*, *set31*, *set38*, *set40*, *seq* | | 2 (16.67) | 0 | 2 (33.33) |
|  |  | *tst*, *seb*, *sec3*, *sel*, *sel-l*, *set24*, *set32*, *set33*, *cylR2* | | 1 (8.33) | 0 | 1 (16.67) |
|  |  | *sed*, *sej*, *ser*, *set9*, *set11*, *set13*, *yent1* | | 1 (8.33) | 1 (16.67) | 0 |
| Secretion System | Type VII Secretion System | *esaA*, *esaG*, *essA*, *essB*, *essC*, *esxA* | | 12 (100.00) | 6 (100.00) | 6 (100.00) |
|  |  | *esaB* | | 10 (83.33) | 5 (83.33) | 5 (83.33) |
|  |  | *esaD*, *esaE*, *esxB*, *esxC*, *esxD* | | 9 (75.00) | 6 (100.00) | 3 (50.00) |

**Abbreviations:** *SCCmec* – Staphylococcal cassette chromosome *mec*; *CC* – Clonal complex; *ST* – Sequence type; *Inc* – Incompatibility; *rep* – Replication type

**Table S4: Individual genotypic profiles of representative *Staphylococcus aureus* isolates, including six bacteraemia isolates and six infective endocarditis isolates, determined by whole genome sequencing**

| **Isolate** | **Isolate Type** | **Ward** | **SCC*mec* Type** | **Pulsotype** | **Spa Type** | **MLST** | **Mobile Genetic Elements** |
| --- | --- | --- | --- | --- | --- | --- | --- |
| SA12A | SAIE | Emergency | N/A | I | t1299 | 152 | **P:** rep5a_1; rep16_3 |
| SA27 | SAIE | Emergency | N/A | N | t9228 | 5 | **P:** rep20_7 |
| SA49 | SAIE | Emergency | N/A | Q | t630 | 45 | **P:** rep5a_1; rep16_3 **IS:** IS*Sau3* (IS*1182*) |
| SA50 | SAB | Neonatal ICU | III (3A) | O | t37 | 239 | **P:** rep20_3; rep21_20; rep7a_22 **IS:** IS*Lgar5* (IS*256*); IS*Sau6* (IS*6*) |
| SA51 | SAIE | Emergency | N/A | E | t355 | 152 | **P:** rep5a_1; rep16_3 |
| SA53 | SAB | Emergency | N/A | H | t1828 | 152 | **P:** rep5a_1; rep16_3 |
| SA55 | SAB | Surgery ICU | N/A | Q | t445 | 508 | **P:** rep5c_1; rep21_22 **IS:** IS*Sau3* (IS*1182*) |
| SA5A | SAIE | Emergency | N/A | I | t355 | 152 | **P:** rep5a_1; rep16_3 |
| SA60 | SAIE | Emergency | N/A | Singleton | t701 | 6 | **P:** rep5a_1; rep16_3 **IS:** IS*Sau3* (IS*1182*) |
| SA61 | SAB | General Surgery | N/A | Untypable | t1451 | 398 | **P:** rep13_1 **IS:** IS*Sau8* (IS*L3*) |
| SA65 | SAB | Male Medical | IVd (2B) | Singleton | t190 | 612 | **P:** rep7c_1; rep20_3; rep10_4 **IS:** IS*Sau6* (IS*6*); IS*Lgar5* (IS*256*) **CN:** CN_2257_IS*Sau6*; CN_12274_IS*Sau6* |
| SA81 | SAB | Adult ICU | N/A | A | t346 | 15 | **P:** rep5a_1; rep16_3 **IS:** IS*Sep3* (IS*200*/IS*605*) |

**Abbreviations:** *NA* – Not applicable; *SAIE* – *Staphylococcus aureus* infective endocarditis; *SAB* – *Staphylococcus aureus* bacteraemia;
*SCCmec* – Staphylococcal cassette chromosome *mec*; *MLST* – Multilocus sequence typing; *IS* – Insertion sequence; *CN* – Composite transposon; *ICU* – Intensive care unit

**P:** Plasmid; **IS:** Insertion sequence; **CN:** Composite transposon

**References:**

Al-Talib H, Yean C, Al-Khateeb A, Hassan H, Singh K-K, Al-Jashamy K, Ravichandran M (2009) A pentaplex PCR assay for the rapid detection of methicillin-resistant
*Staphylococcus aureus* and Panton-Valentine leucocidin. BMC Microbiol 9:113. https://doi.org/10.1186/1471-2180-9-113.

Chmagh AA, Abd Al-Abbas MJ (2019) Comparison between the coagulase (*coa* and *vwb*) genes in *Staphylococcus aureus* and other staphylococci. Gene Rep 16:100410. https://doi.org/10.1016/j.genrep.2019.100410.

Goudarzi M, Seyedjavadi SS, Nasiri MJ, Goudarzi H, Sajadi Nia R, Dabiri H (2017) Molecular characteristics of methicillin-resistant *Staphylococcus aureus* (MRSA) strains isolated from patients with bacteremia based on MLST, SCC*mec*, *spa*, and *agr* locus types analysis. Microb Pathog 104:328–335. https://doi.org/10.1016/j.micpath.2017.01.055.

Hirotaki S, Sasaki T, Kuwahara-Arai K, Hiramatsu K (2011) Rapid and accurate identification of human-associated staphylococci by use of multiplex PCR. J Clin Microbiol 49:3627–3631. https://doi.org/10.1128/jcm.00488-11.

Jarraud S, Mougel C, Thioulouse J, Lina G, Meugnier H, Forey F, Nesme X, Etienne J, Vandenesch F (2002) Relationships between *Staphylococcus aureus* genetic background, virulence factors, *agr* groups (alleles), and human disease. Infect Immun 70:631–641. https://doi.org/10.1128/IAI.70.2.631-641.2002.

Martineau F, Picard FOJ, Lansac N, MéNard C, Roy PH, Ouellette M, Bergeron MG (2000) Correlation between the resistance genotype determined by multiplex PCR assays and the antibiotic susceptibility patterns of *Staphylococcus aureus* and *Staphylococcus epidermidis*. Antimicrob Agents Chemother 44:231–238. https://doi.org/10.1128/aac.44.2.231-238.2000.

Monday SR, Bohach GA (1999) Use of multiplex PCR to detect classical and newly described pyrogenic toxin genes in staphylococcal isolates. J Clin Microbiol 37:3411–3414. https://doi.org/10.1128/JCM.37.10.3411-3414.1999.

Palazzolo-Ballance AM, Reniere ML, Braughton KR, Sturdevant DE, Otto M, Kreiswirth BN, Skaar EP, DeLeo FR (2008) Neutrophil microbicides induce a pathogen survival response in community-associated methicillin-resistant *Staphylococcus aureus*. J Immunol 180:500–509. https://doi.org/10.4049/jimmunol.180.1.500.

Parastan R, Kargar M, Solhjoo K, Kafilzadeh F (2020) A synergistic association between adhesion-related genes and multidrug resistance patterns of *Staphylococcus aureus* isolates from different patients and healthy individuals. J Glob Antimicrob Resist 22:379–385. https://doi.org/10.1016/j.jgar.2020.02.025.

Parvin M, Ali M, Talukder S, Nahar A, Chowdhury EH, Rahman M, Islam M (2021) Prevalence and multidrug resistance pattern of methicillin resistant *S. aureus* isolated from frozen chicken meat in Bangladesh. Microorganisms 9:636. https://doi.org/10.3390/microorganisms9030636

Peacock SJ, Moore CE, Justice A, Kantzanou M, Story L, Mackie K, O'Neill G, Day NP (2002) Virulent combinations of adhesin and toxin genes in natural populations of
*Staphylococcus aureus*. Infect Immun 70:4987–96. https://doi.org/10.1128/iai.70.9.4987-4996.2002.

Vu BG, Stach CS, Salgado-Pabón W, Diekema DJ, Gardner SE, Schlievert PM (2014) Superantigens of *Staphylococcus aureus* from patients with diabetic foot ulcers. J Infect Dis 210:1920–1927. https://doi.org/10.1093/infdis/jiu350.

Zdzalik M, Karim AY, Wolski K, Buda P, Wojcik K, Brueggemann S, Wojciechowski P, Eick S, Calander A-M, Jonsson I-M, Kubica M, Polakowska K, Miedzobrodzki J, Wladyka B, Potempa J, Dubin G (2012) Prevalence of genes encoding extracellular proteases in *Staphylococcus aureus* — important targets triggering immune response *in vivo*. FEMS Immunol Med Microbiol 66:220–229. https://doi.org/10.1111/j.1574-695X.2012.01005.x.
