# Supplementary figures and images for: Genomic comparison of Staphylococcus aureus isolates from patients with bacteraemia and infective endocarditis at public hospitals in Gauteng, South Africa
Source: World J Microbiol Biotechnol. 2026 Jul 27;42(8):430. doi: 10.1007/s11274-026-05145-z (PMC13407745; doi:10.1007/s11274-026-05145-z)

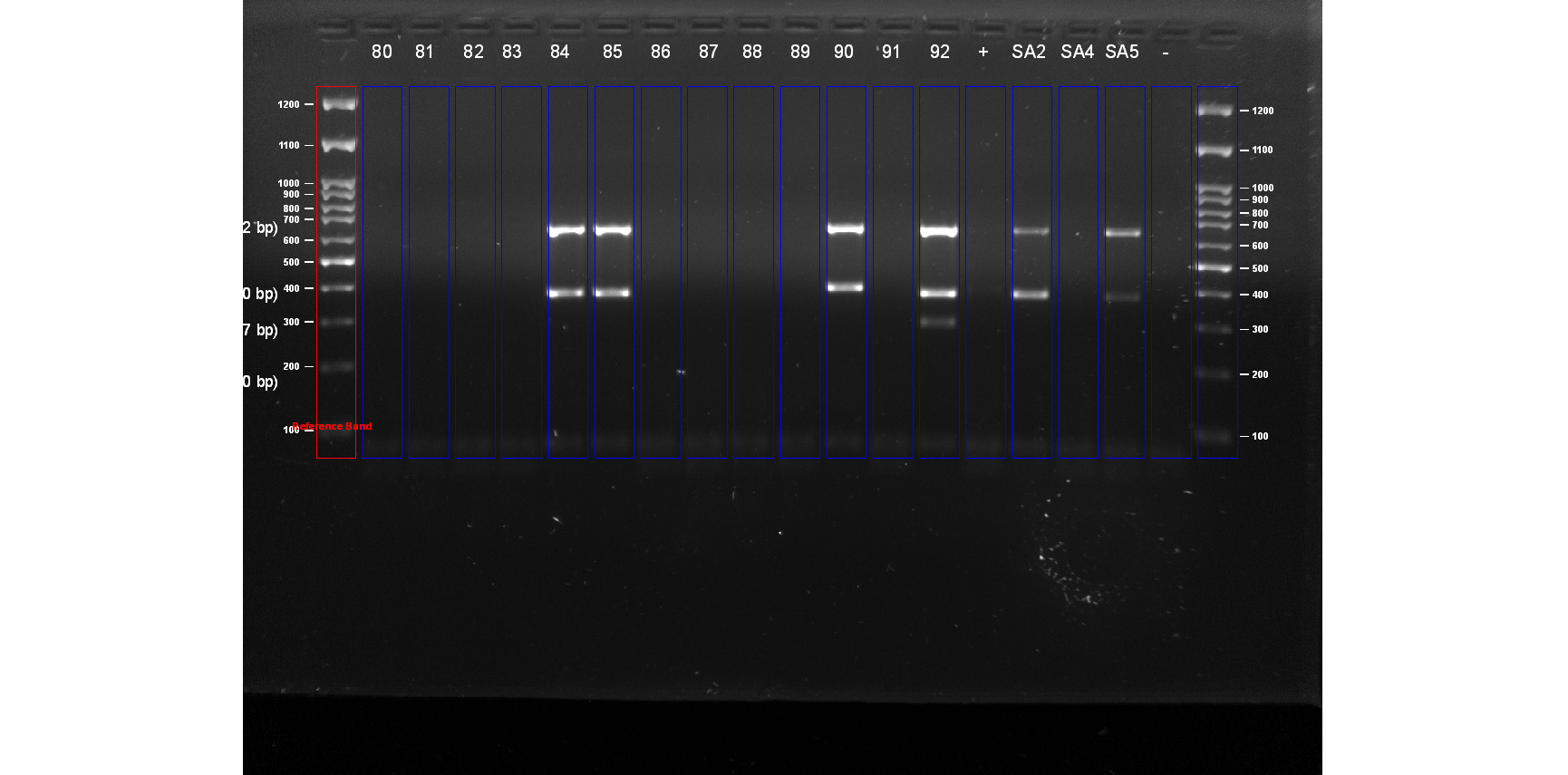

Supplement: Supplementary file 1 — Supplementary file1 (ZIP 25298 KB) [file 11274_2026_5145_MOESM1_ESM.zip › Virulence_tst, seg, sec, sel-u_(#80-92).tif]

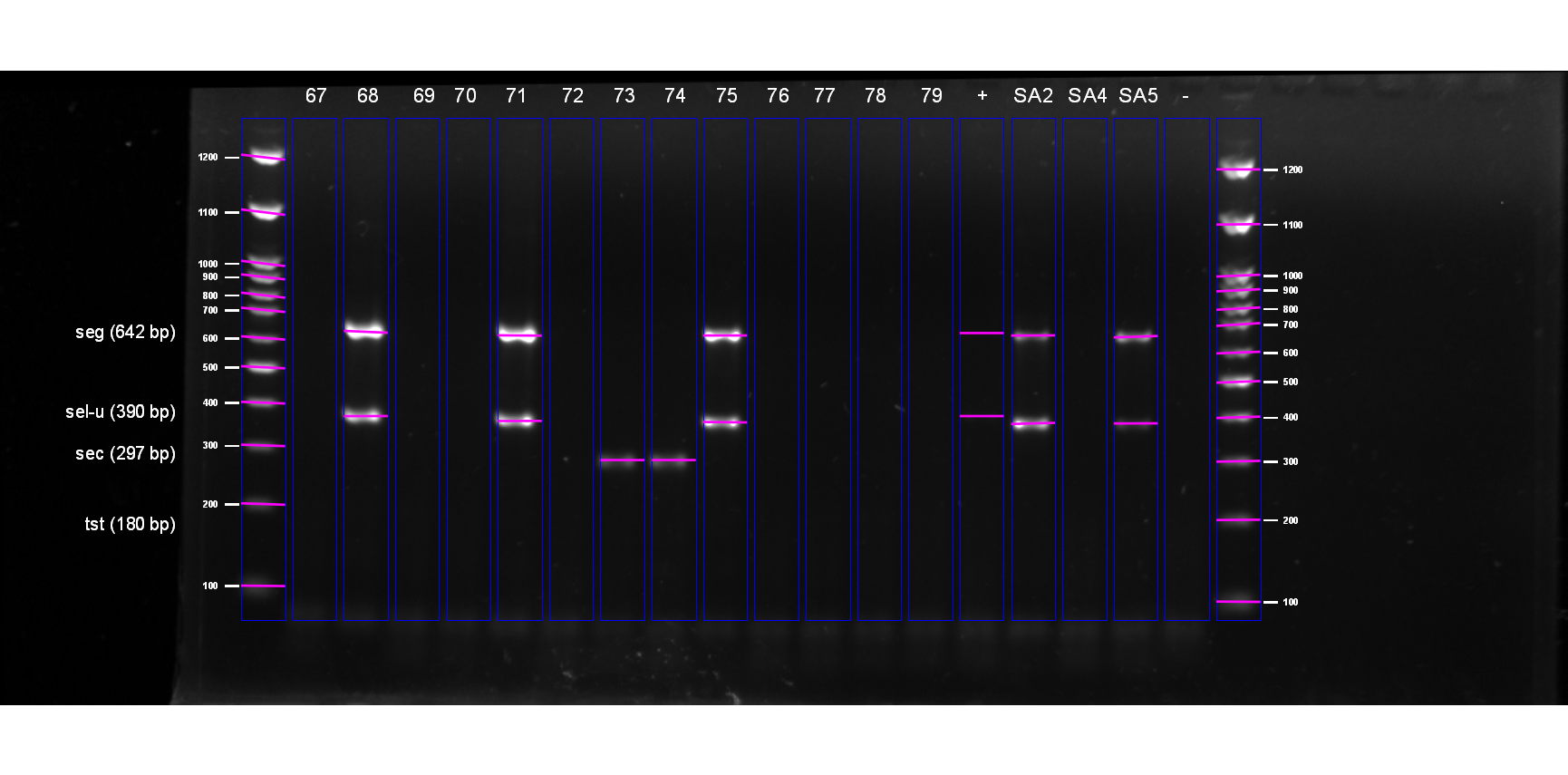

Supplement: Supplementary file 1 — Supplementary file1 (ZIP 25298 KB) [file 11274_2026_5145_MOESM1_ESM.zip › Virulence_tst, seg, sec, sel-u_(#67-79).tif]

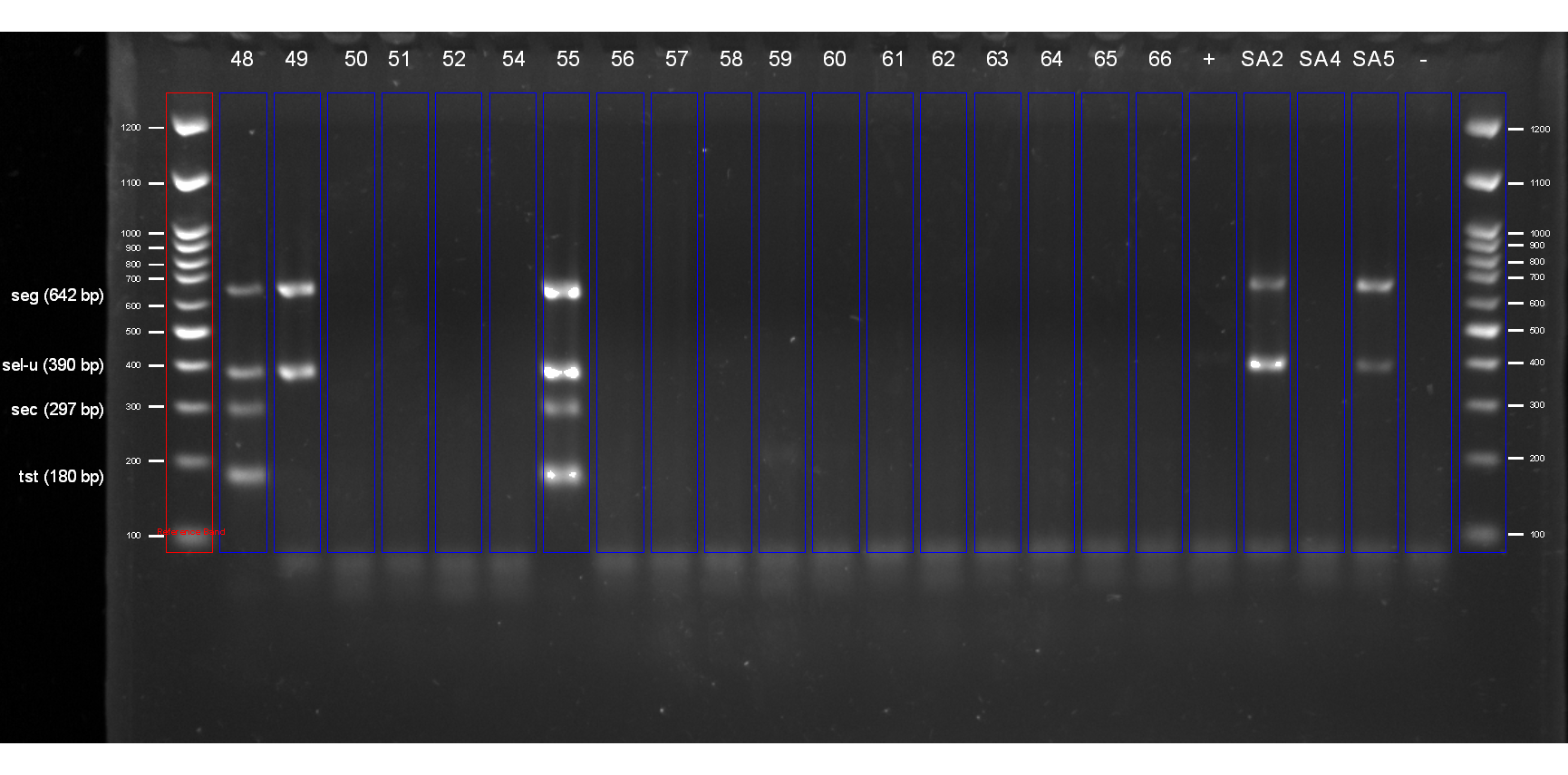

Supplement: Supplementary file 1 — Supplementary file1 (ZIP 25298 KB) [file 11274_2026_5145_MOESM1_ESM.zip › Virulence_tst, seg, sec, sel-u_(#48-52, 54-66).tif]

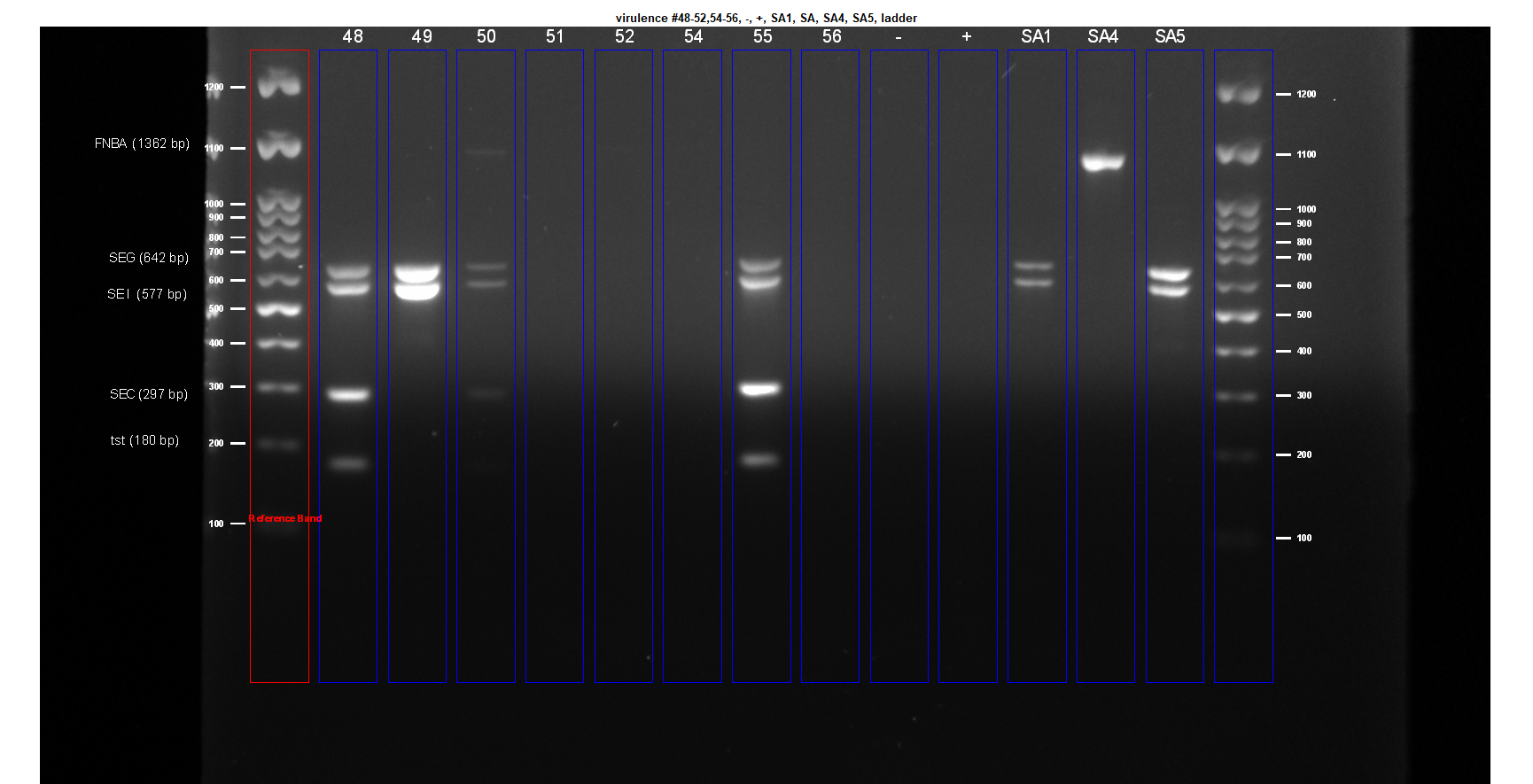

Supplement: Supplementary file 1 — Supplementary file1 (ZIP 25298 KB) [file 11274_2026_5145_MOESM1_ESM.zip › Virulence_tst, sec, sei, seg, fnba_(#48-52, 54-56).tif]

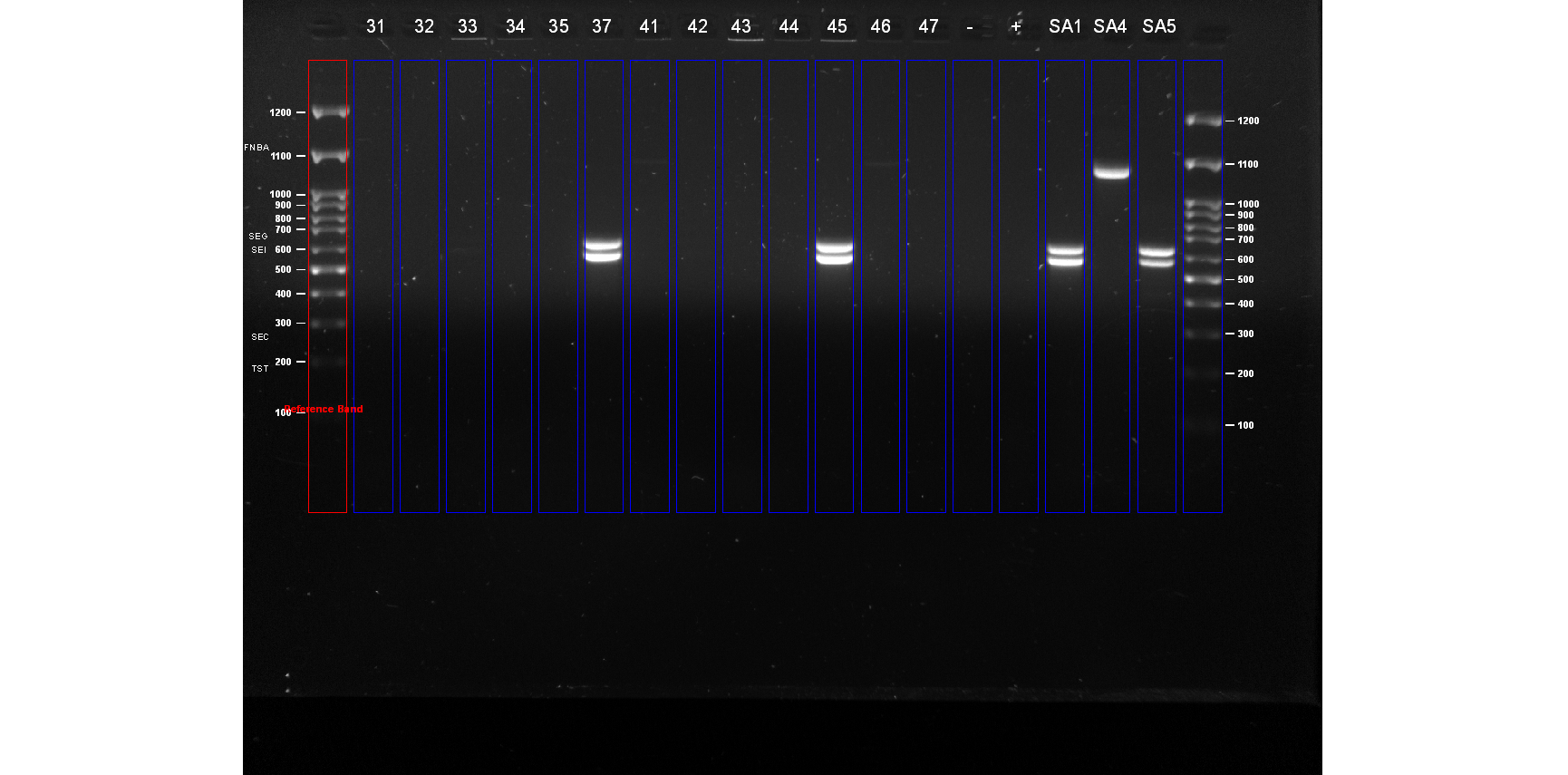

Supplement: Supplementary file 1 — Supplementary file1 (ZIP 25298 KB) [file 11274_2026_5145_MOESM1_ESM.zip › Virulence_tst, sec, sei, seg, fnba_(#31-35, 37, 41-47).tif]

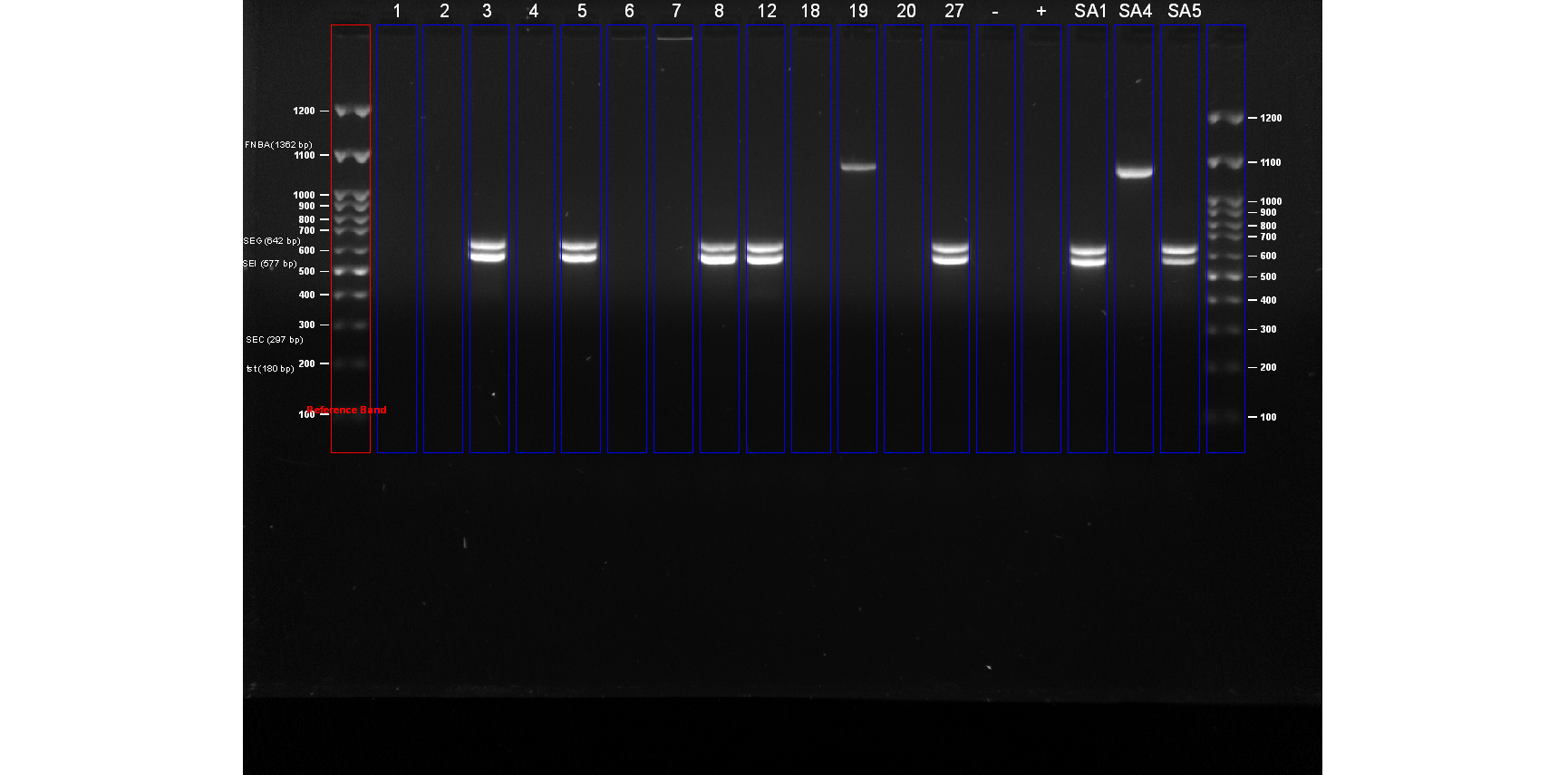

Supplement: Supplementary file 1 — Supplementary file1 (ZIP 25298 KB) [file 11274_2026_5145_MOESM1_ESM.zip › Virulence_tst, sec, sei, seg, fnba_(#1-8, 12, 18-20,27).tif]

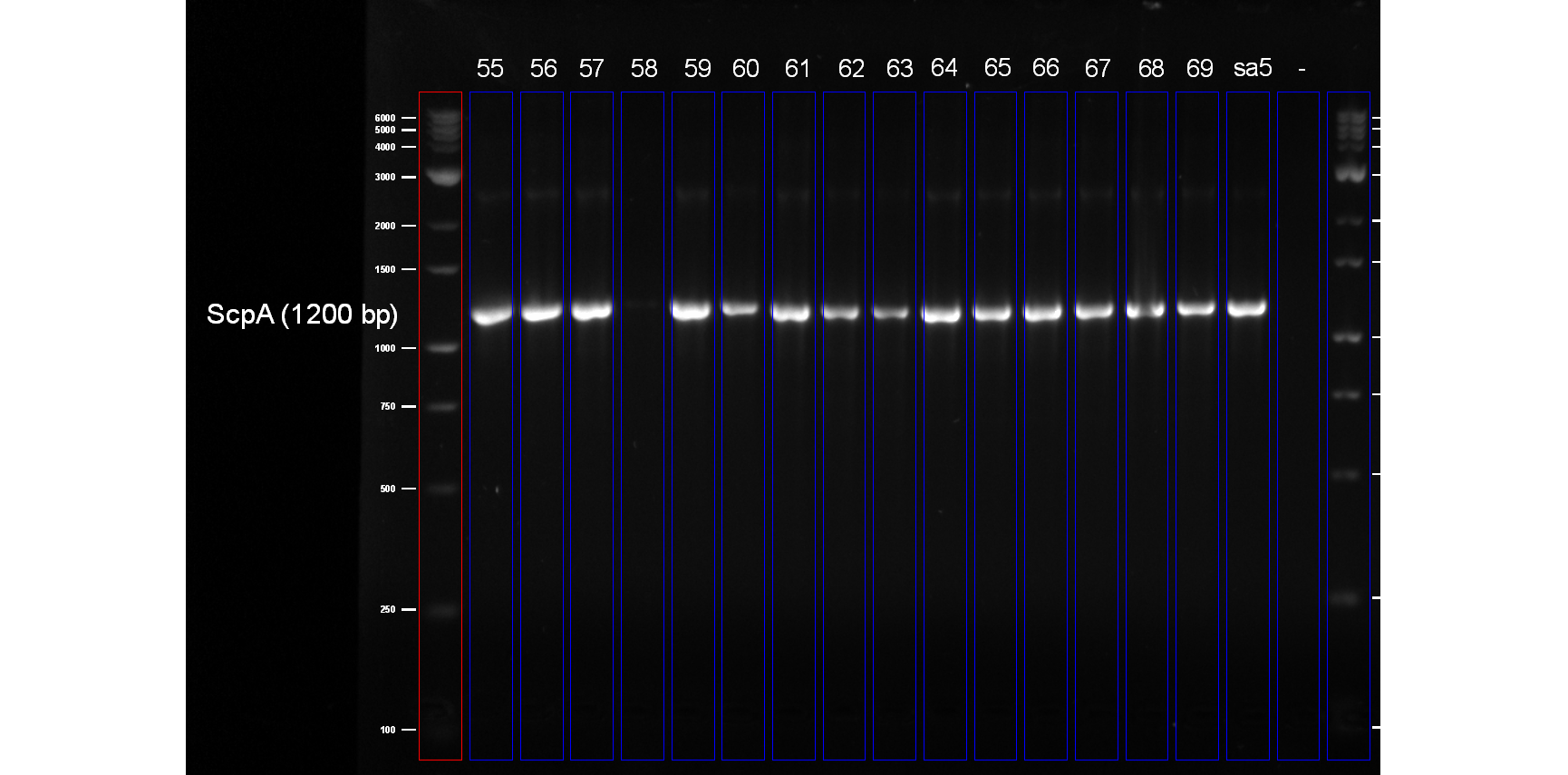

Supplement: Supplementary file 1 — Supplementary file1 (ZIP 25298 KB) [file 11274_2026_5145_MOESM1_ESM.zip › Singleplex PCR_Virulence_ScpA (#55-69).tif]

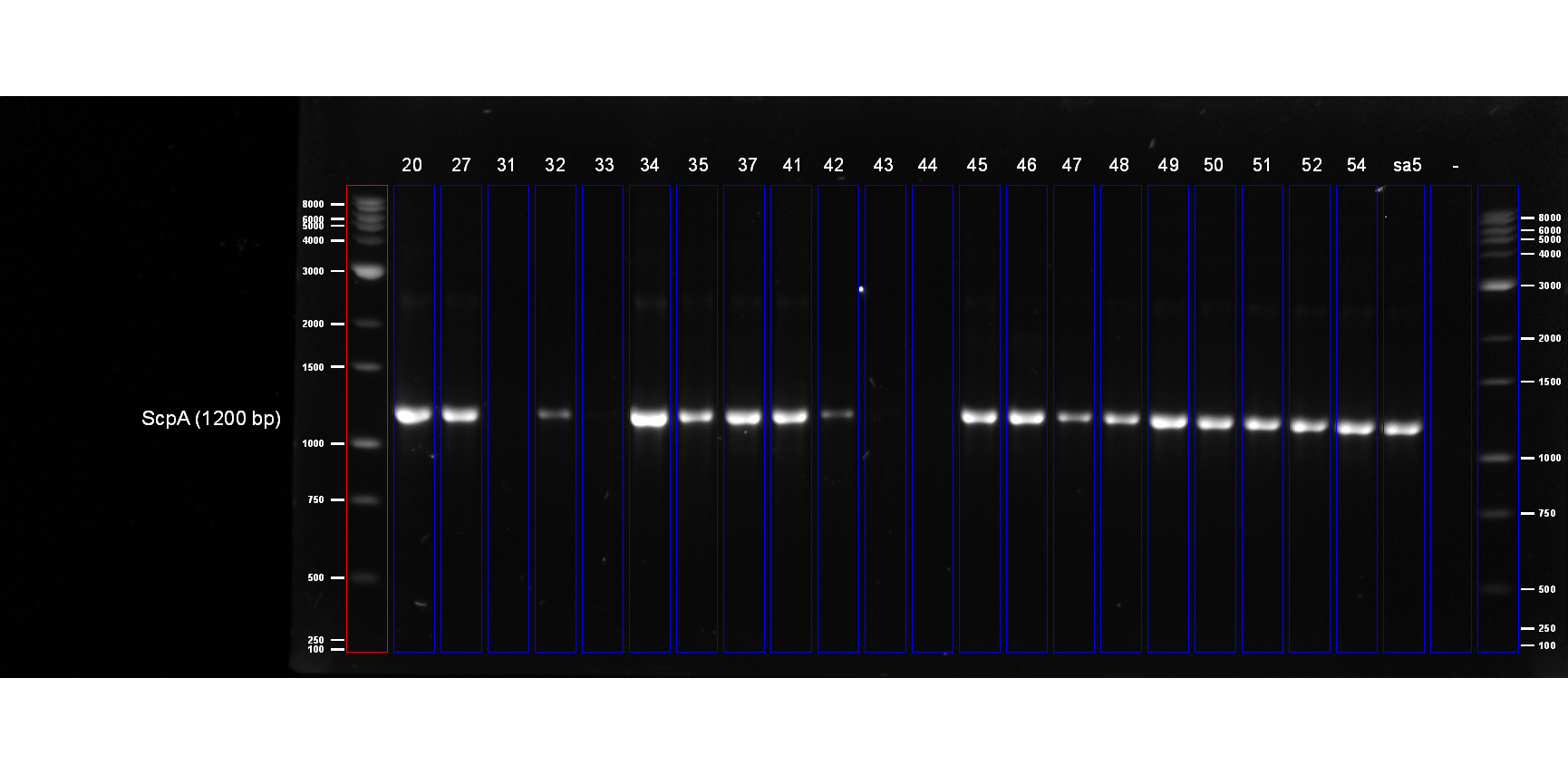

Supplement: Supplementary file 1 — Supplementary file1 (ZIP 25298 KB) [file 11274_2026_5145_MOESM1_ESM.zip › Singleplex PCR_Virulence_ScpA (#20, 27, 31-35, 37, 41-52, 54).tif]

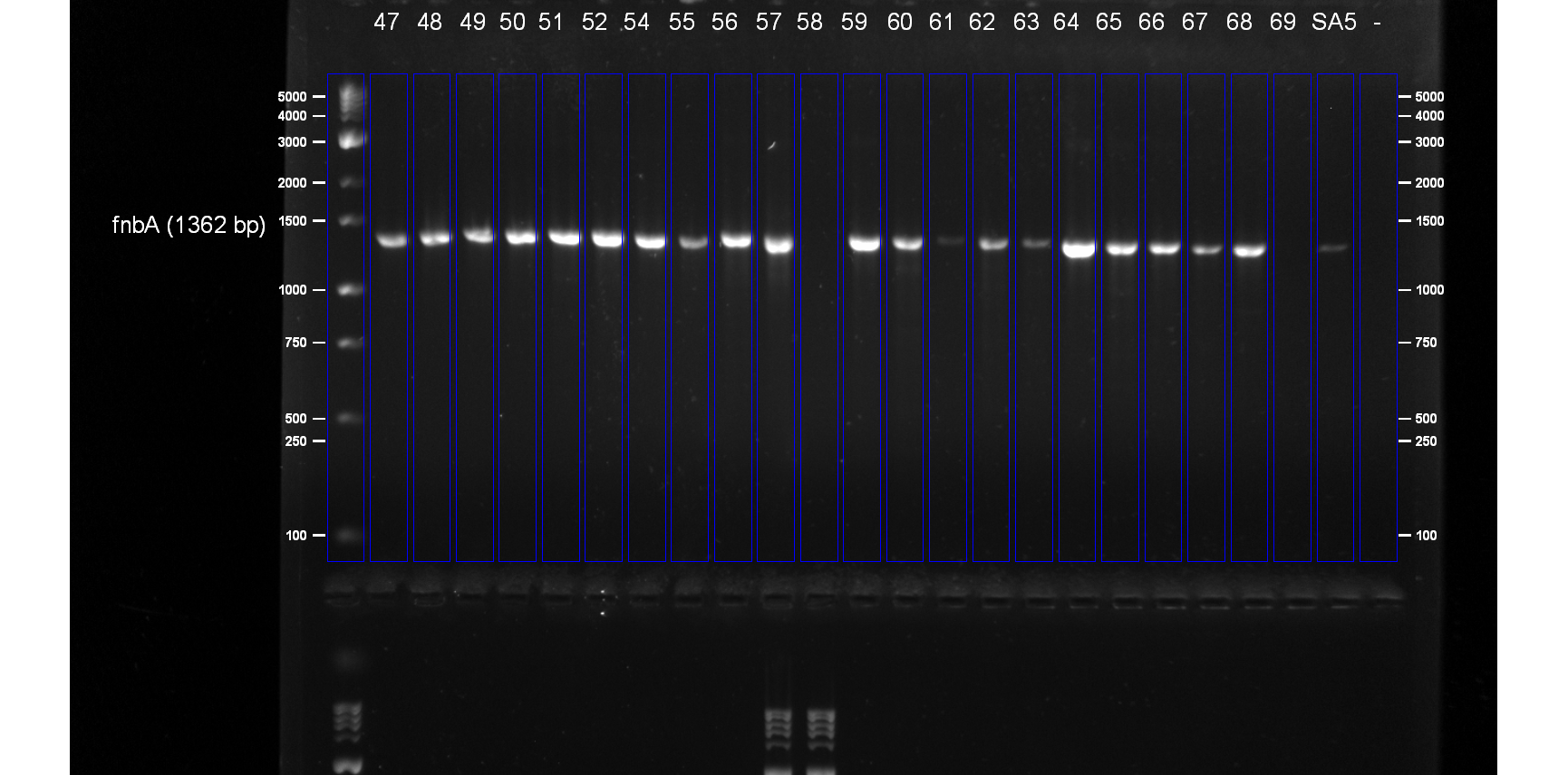

Supplement: Supplementary file 1 — Supplementary file1 (ZIP 25298 KB) [file 11274_2026_5145_MOESM1_ESM.zip › Singleplex PCR_Virulence_FNBP (#47-52, 54-69).tif]

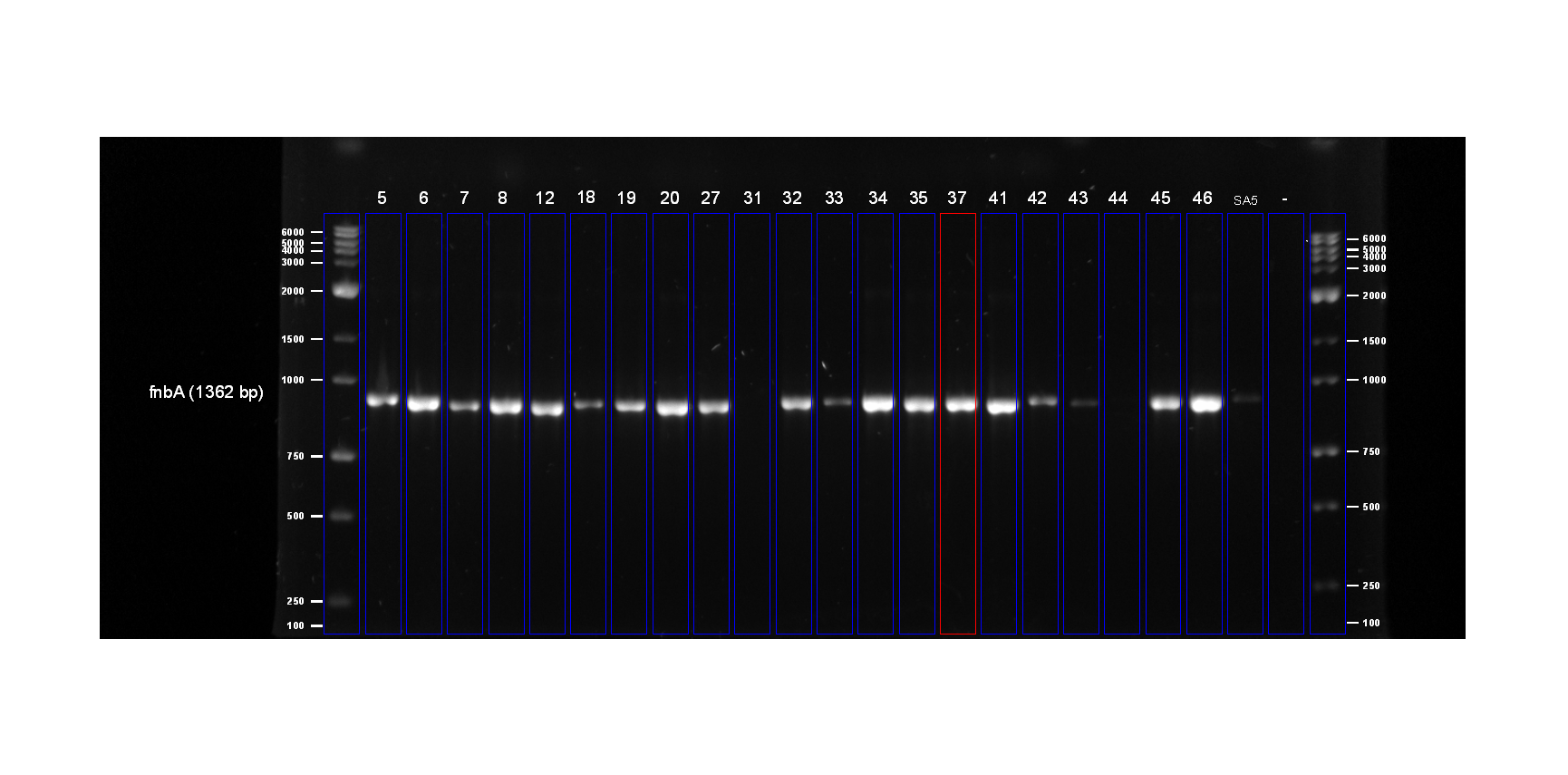

Supplement: Supplementary file 1 — Supplementary file1 (ZIP 25298 KB) [file 11274_2026_5145_MOESM1_ESM.zip › Singleplex PCR_Virulence_FNBP (#5-8, 12,18-20, 27, 31-35, 37, 41-46).tif]

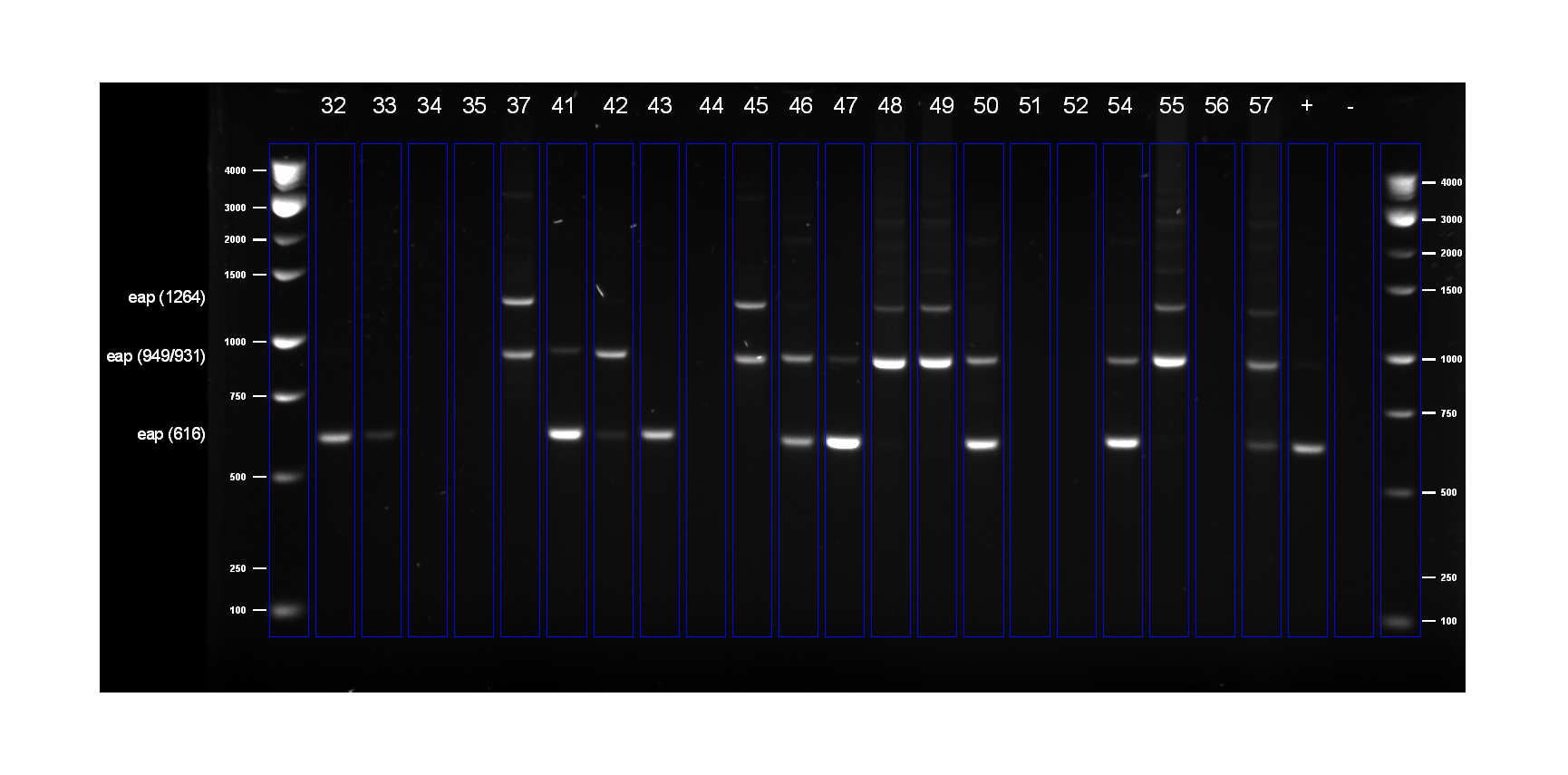

Supplement: Supplementary file 1 — Supplementary file1 (ZIP 25298 KB) [file 11274_2026_5145_MOESM1_ESM.zip › Singleplex PCR_Virulence_EAP (#32-35, 37, 41-52, 54 - 57).tif]

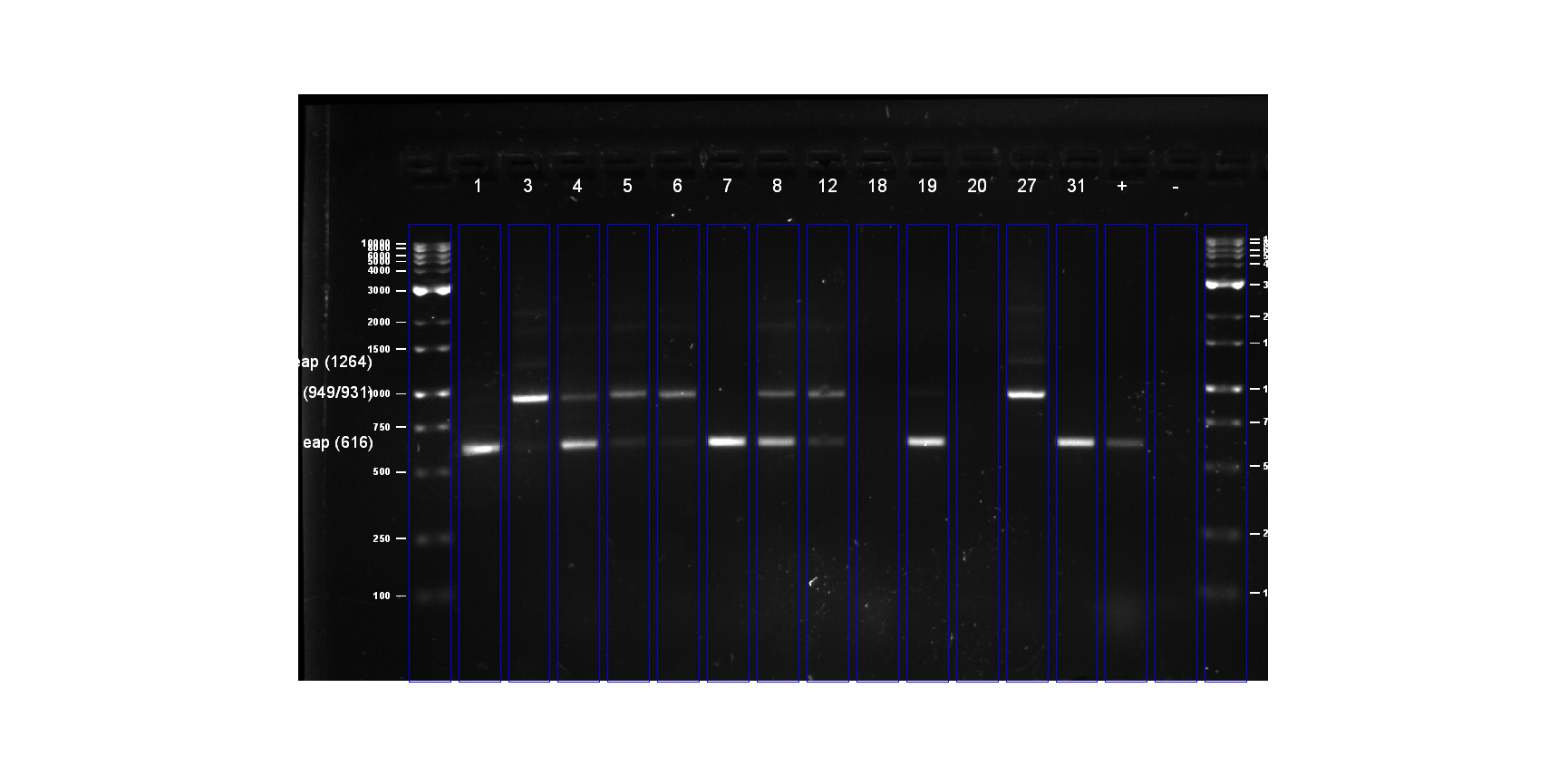

Supplement: Supplementary file 1 — Supplementary file1 (ZIP 25298 KB) [file 11274_2026_5145_MOESM1_ESM.zip › Singleplex PCR_Virulence_EAP (#1, 3-8, 12, 18-20, 27, 31).tif]

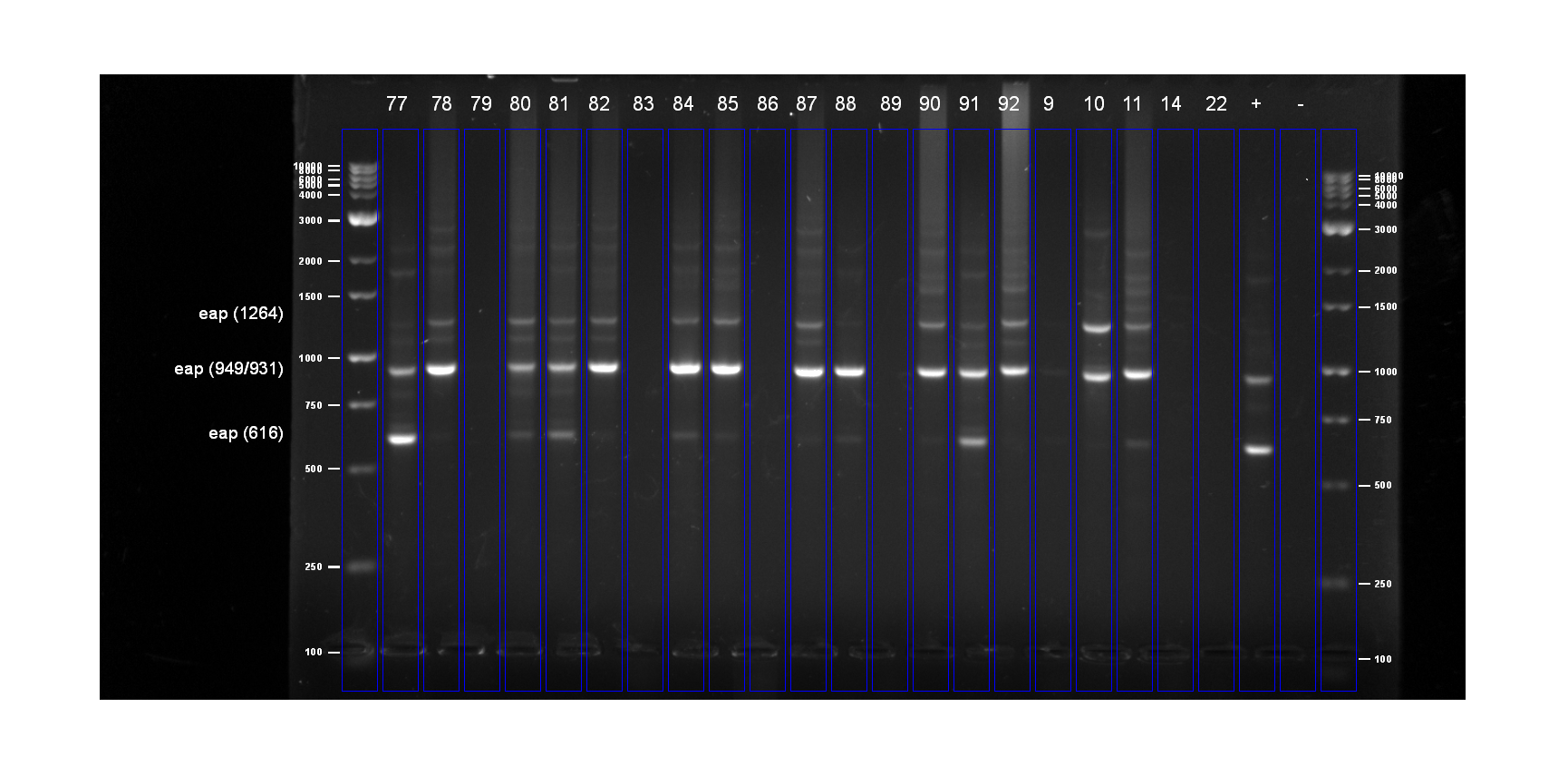

Supplement: Supplementary file 1 — Supplementary file1 (ZIP 25298 KB) [file 11274_2026_5145_MOESM1_ESM.zip › Singleplex PCR_Virulence_EAP (77-92, 9-11, 14, 22).tif]

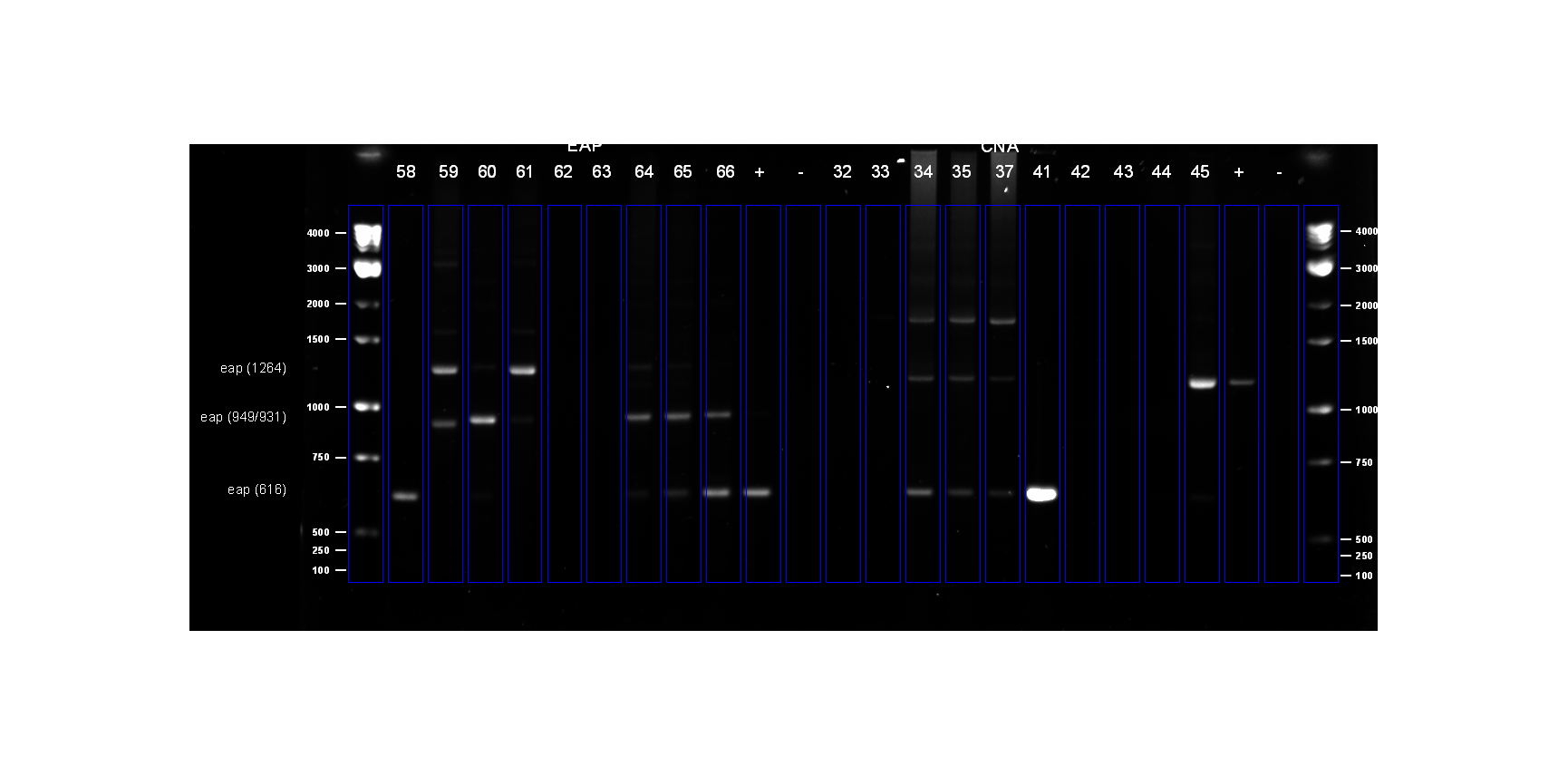

Supplement: Supplementary file 1 — Supplementary file1 (ZIP 25298 KB) [file 11274_2026_5145_MOESM1_ESM.zip › Singleplex PCR_Virulence_EAP (58 - 66)_CNA (#32-35, 41-45).tif]

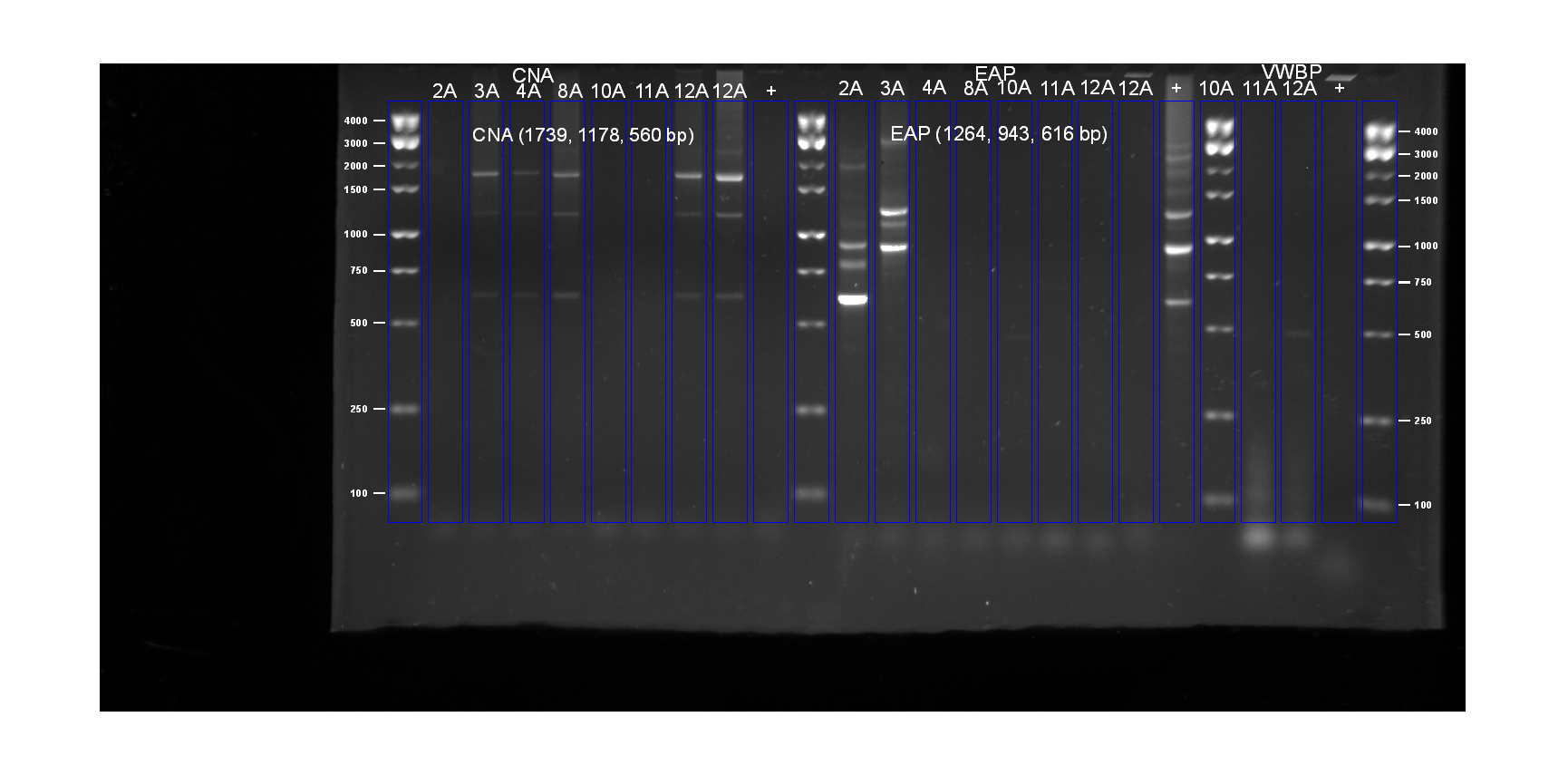

Supplement: Supplementary file 1 — Supplementary file1 (ZIP 25298 KB) [file 11274_2026_5145_MOESM1_ESM.zip › Singleplex PCR_Virulence_CNA, EAP, VWBP ( #2A-12A).tif]

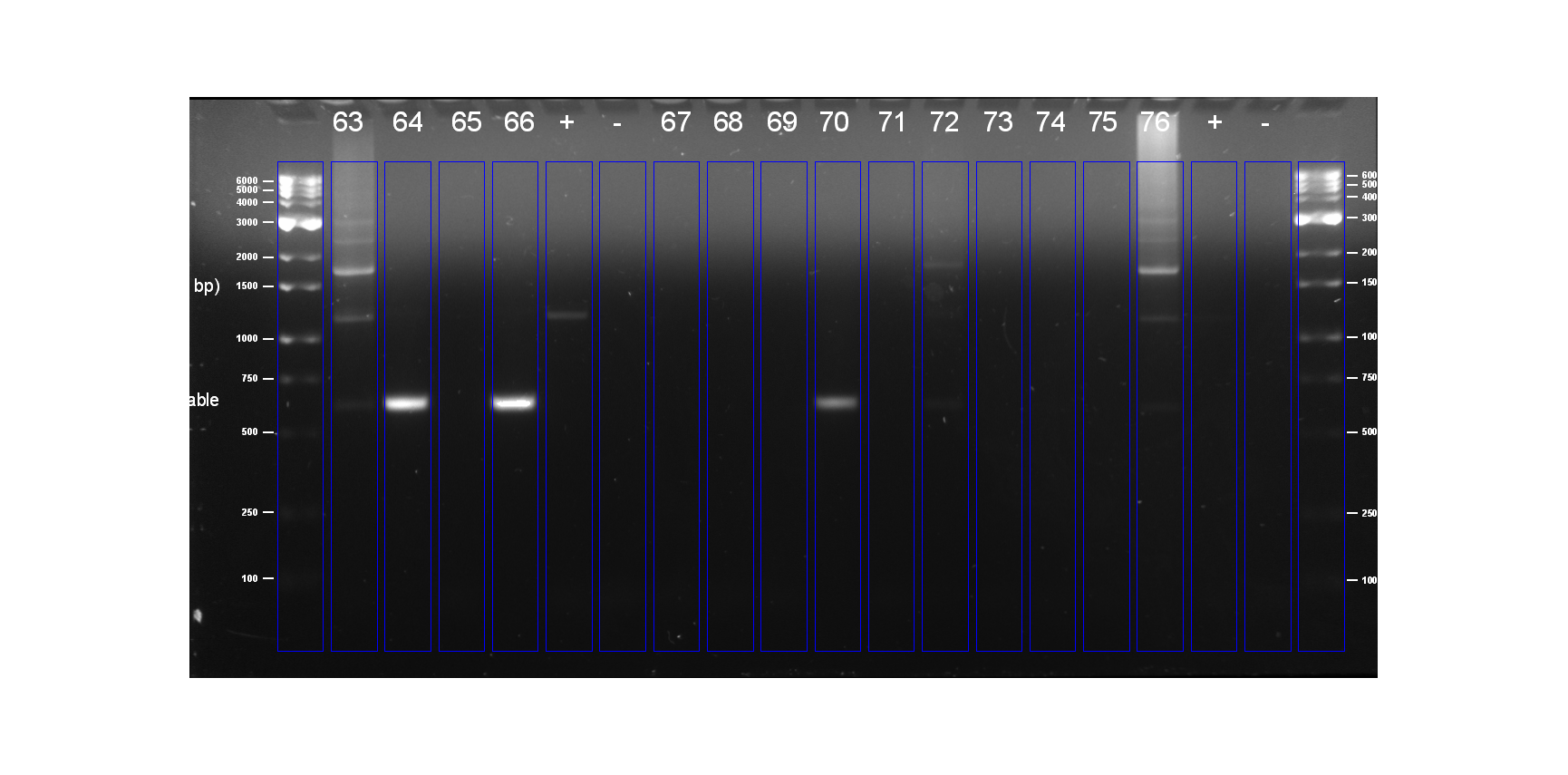

Supplement: Supplementary file 1 — Supplementary file1 (ZIP 25298 KB) [file 11274_2026_5145_MOESM1_ESM.zip › Singleplex PCR_Virulence_CNA (#63-76).tif]

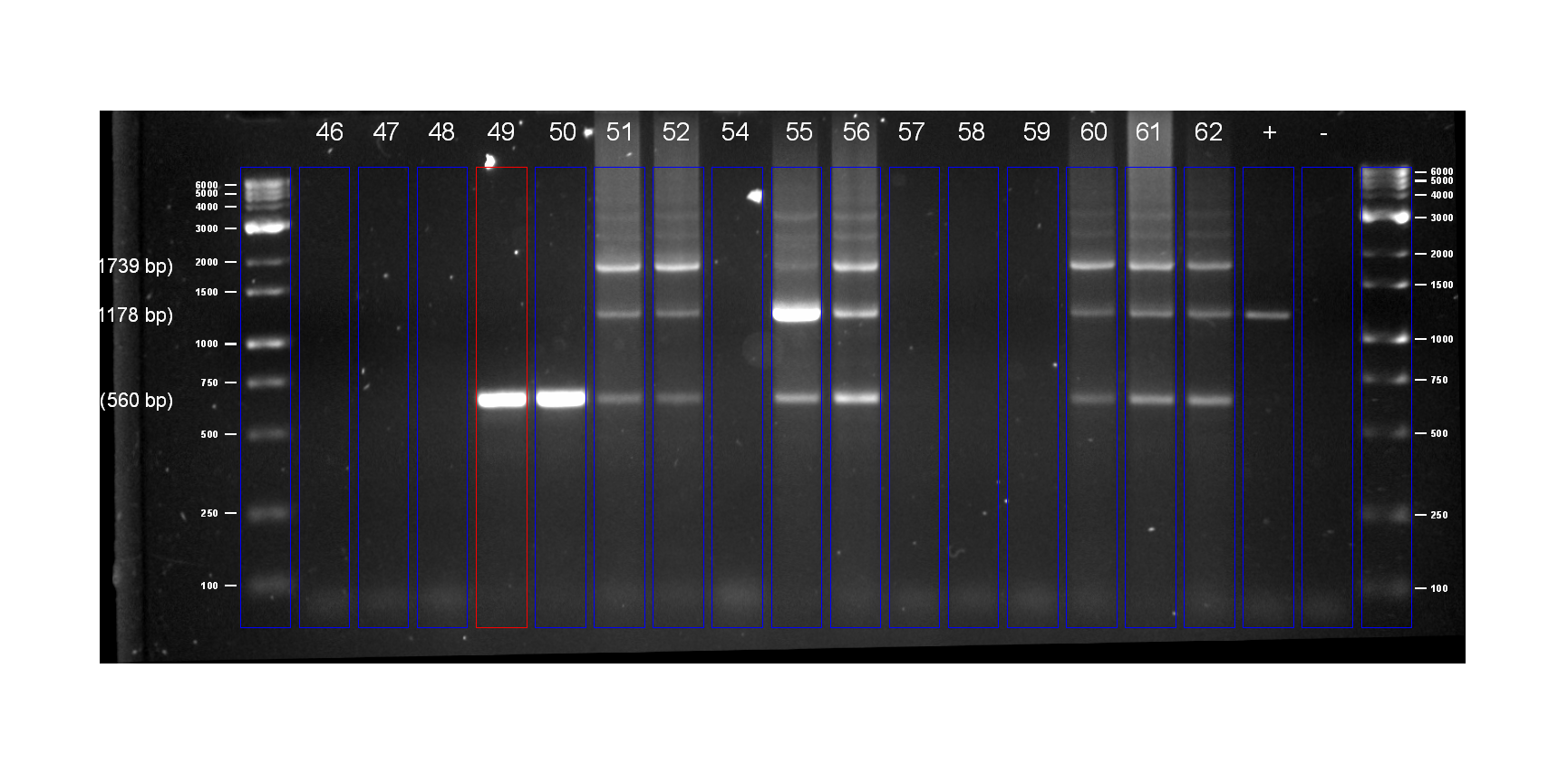

Supplement: Supplementary file 1 — Supplementary file1 (ZIP 25298 KB) [file 11274_2026_5145_MOESM1_ESM.zip › Singleplex PCR_Virulence_CNA (#46-52, 54-62).tif]

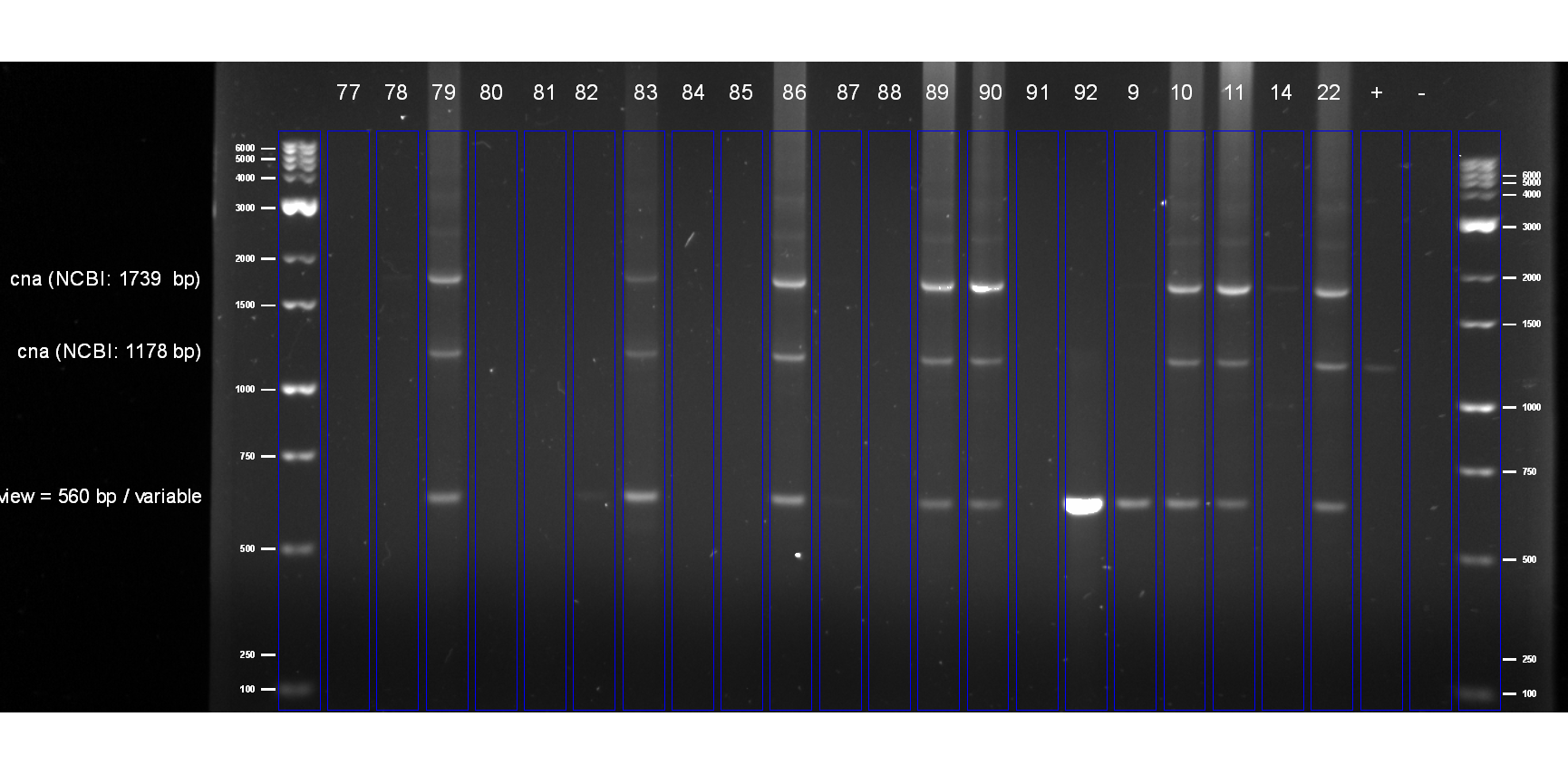

Supplement: Supplementary file 1 — Supplementary file1 (ZIP 25298 KB) [file 11274_2026_5145_MOESM1_ESM.zip › Singleplex PCR_Virulence_CNA ( #77-92, 9-11, 14, 22).tif]

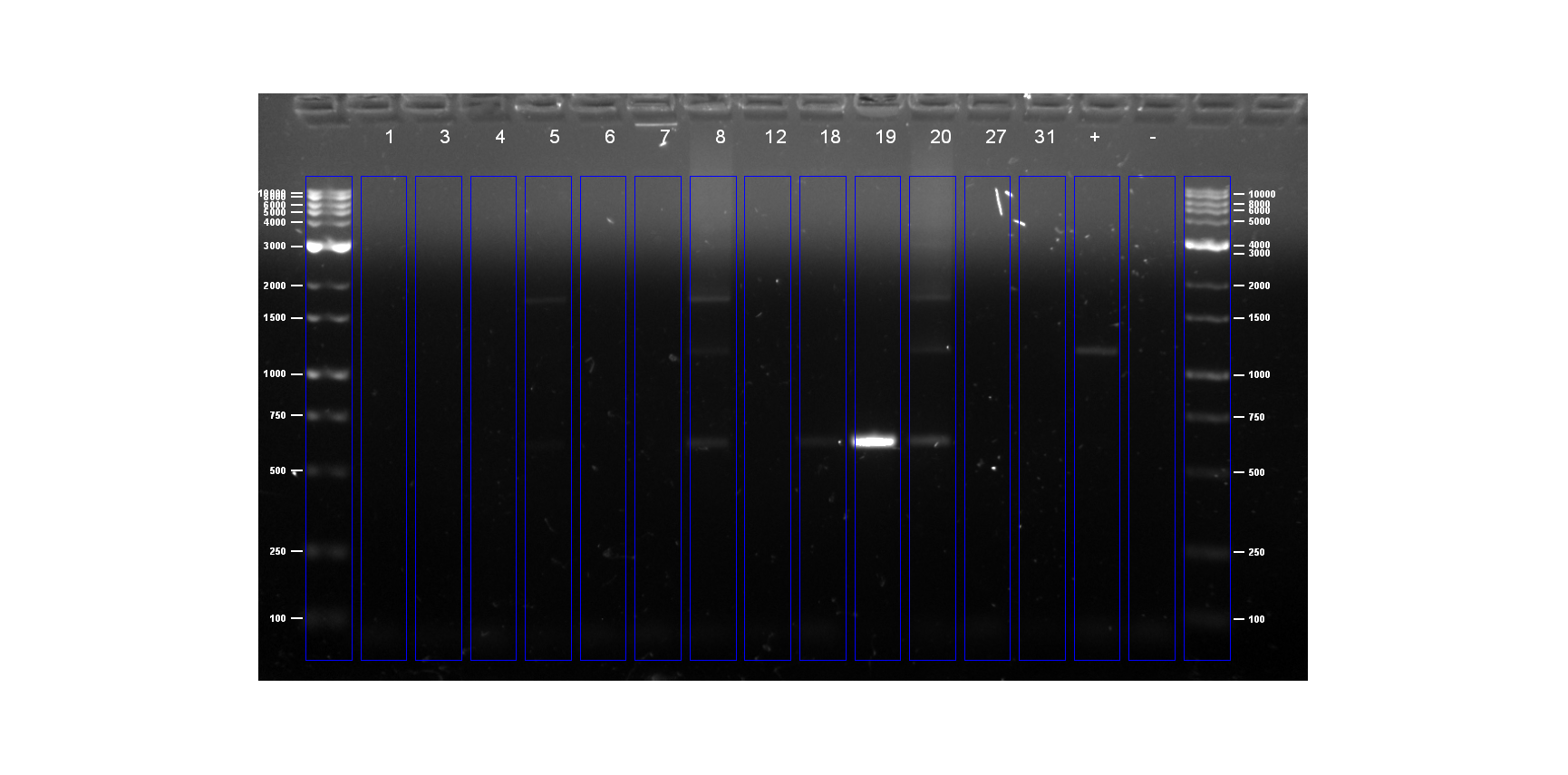

Supplement: Supplementary file 1 — Supplementary file1 (ZIP 25298 KB) [file 11274_2026_5145_MOESM1_ESM.zip › Singleplex PCR_Virulence_CNA ( #1, 3-8, 12, 18-20, 27, 31).tif]

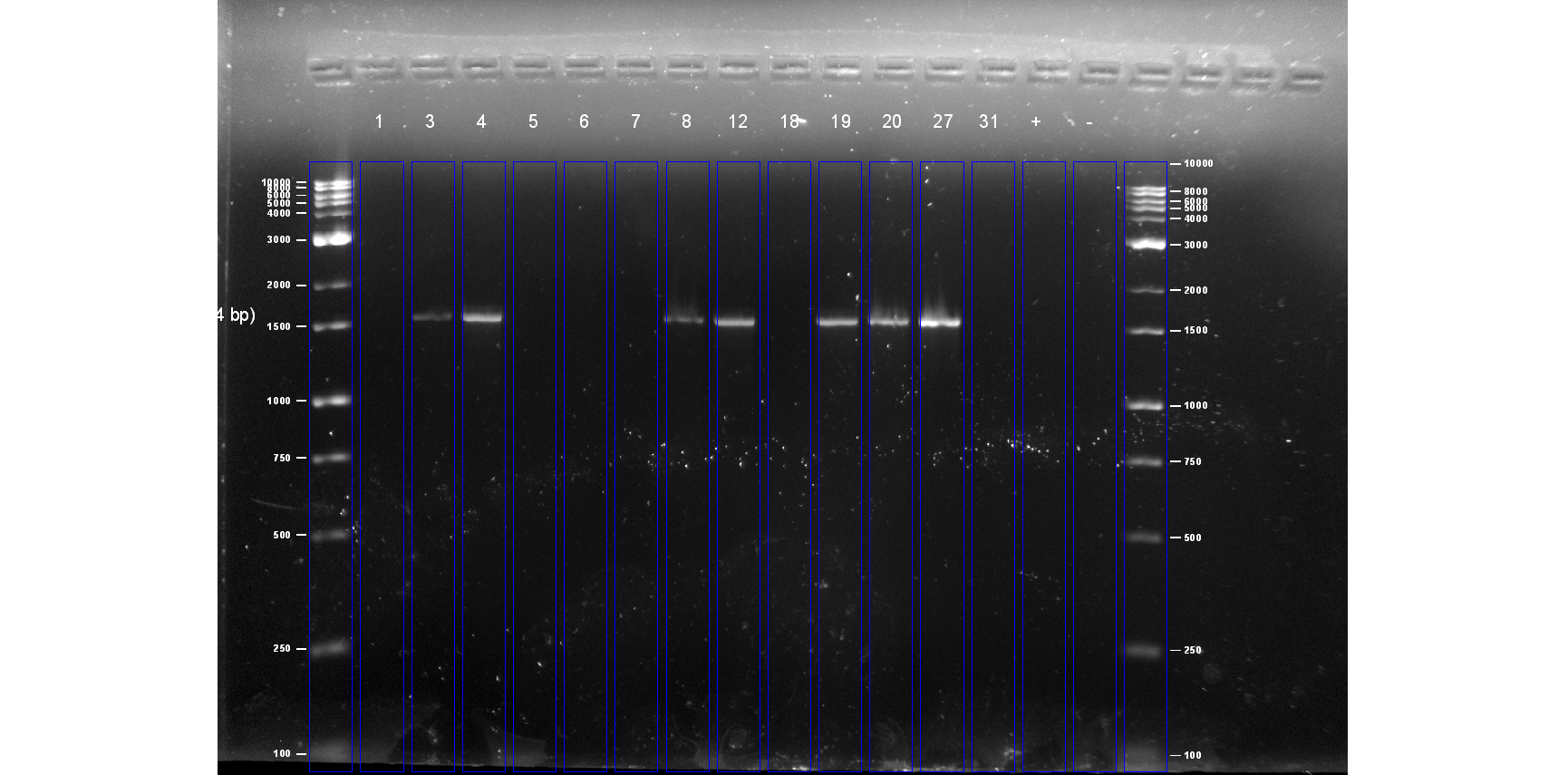

Supplement: Supplementary file 1 — Supplementary file1 (ZIP 25298 KB) [file 11274_2026_5145_MOESM1_ESM.zip › Singleplex PCR_Virulence_clfA (#1, 3-8, 12, 18-20, 27, 31).tif]

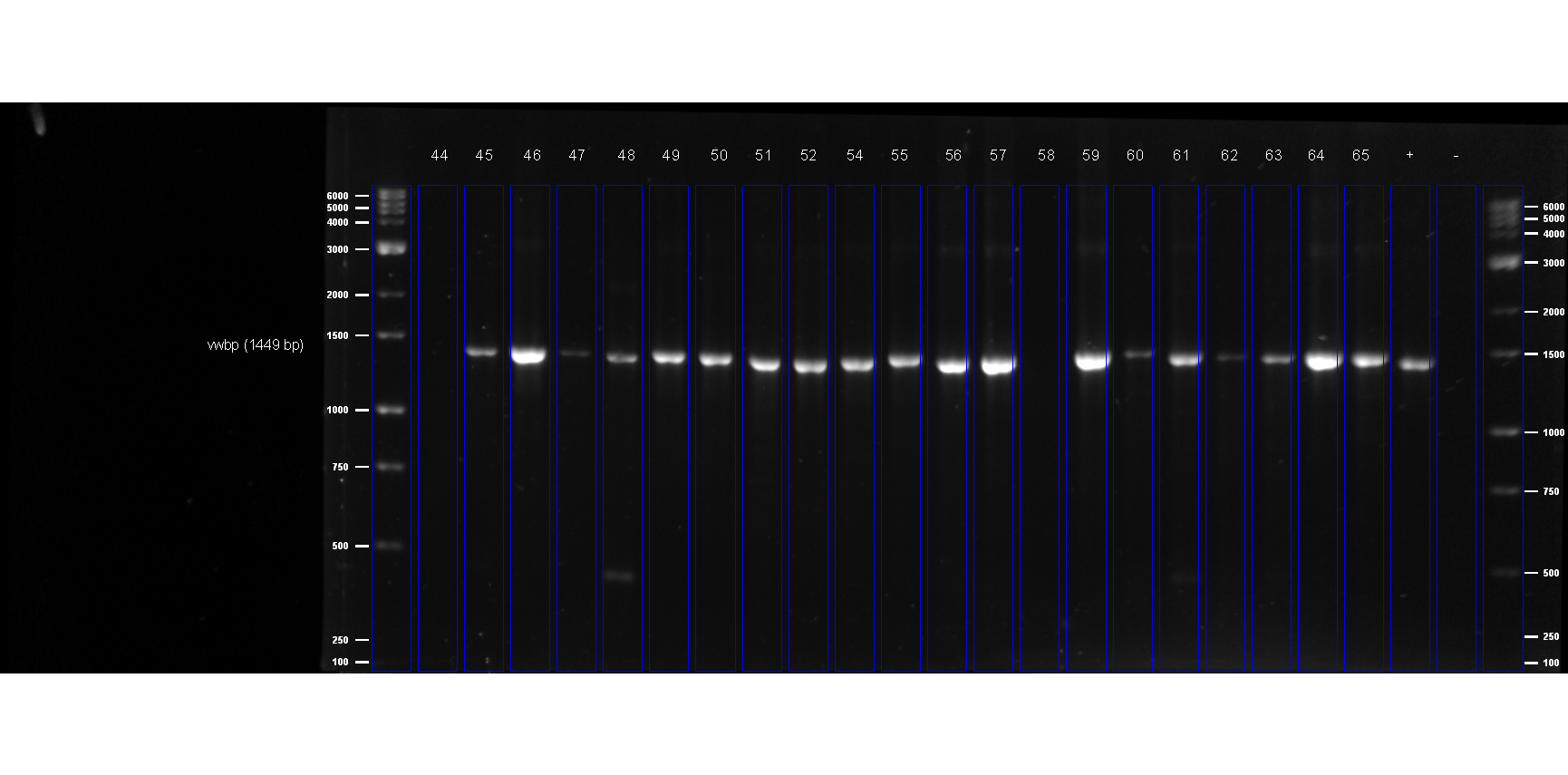

Supplement: Supplementary file 1 — Supplementary file1 (ZIP 25298 KB) [file 11274_2026_5145_MOESM1_ESM.zip › Singleplex PCR_Virulence_ vwbp (#44-52, 54-65).tif]

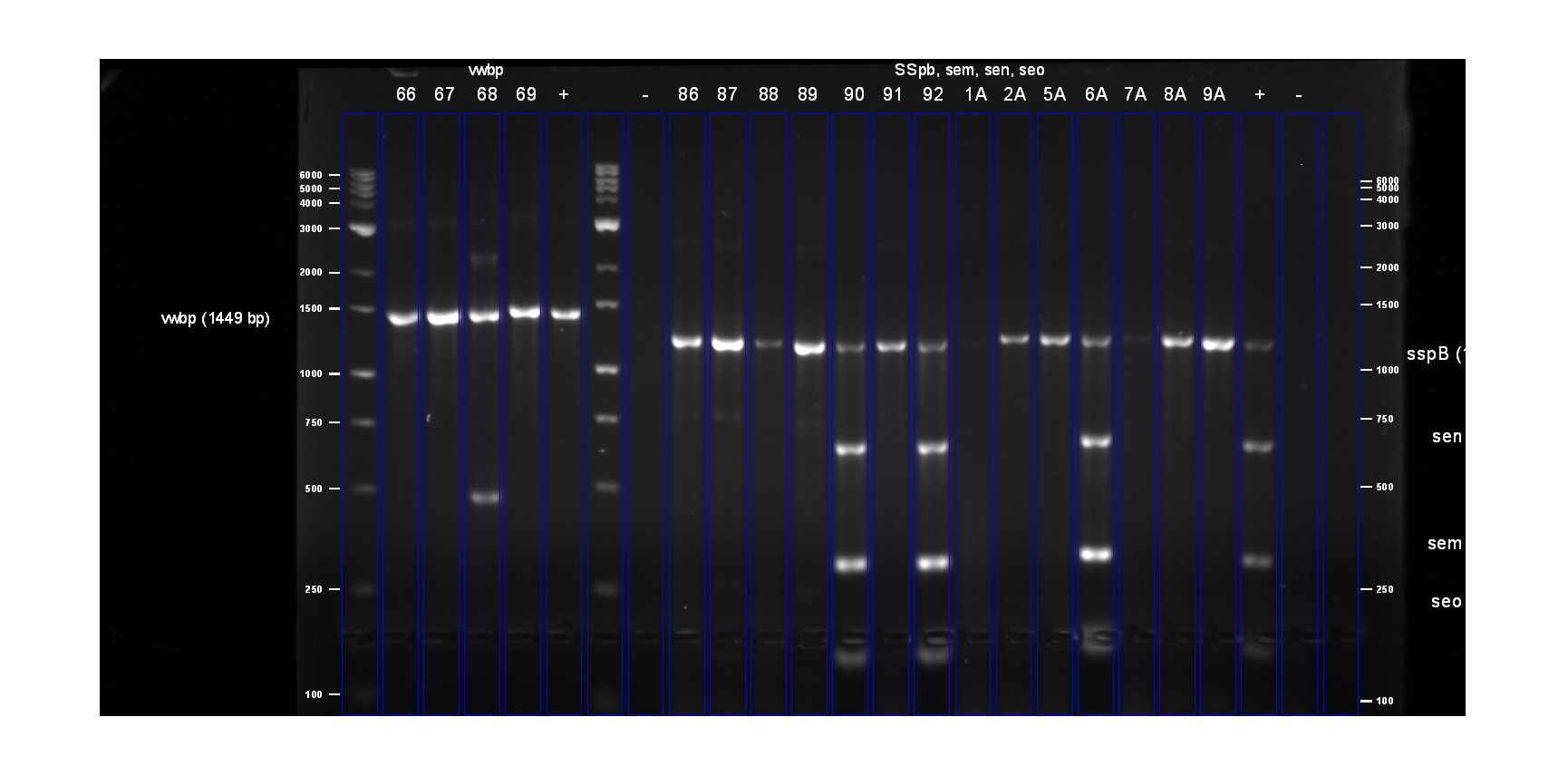

Supplement: Supplementary file 1 — Supplementary file1 (ZIP 25298 KB) [file 11274_2026_5145_MOESM1_ESM.zip › Multiplex PCR_Virulence_vwbp (#66-69)_sem, sen, seo, ssbp (#86-92, 1A-2A, 5A-9A).tif]

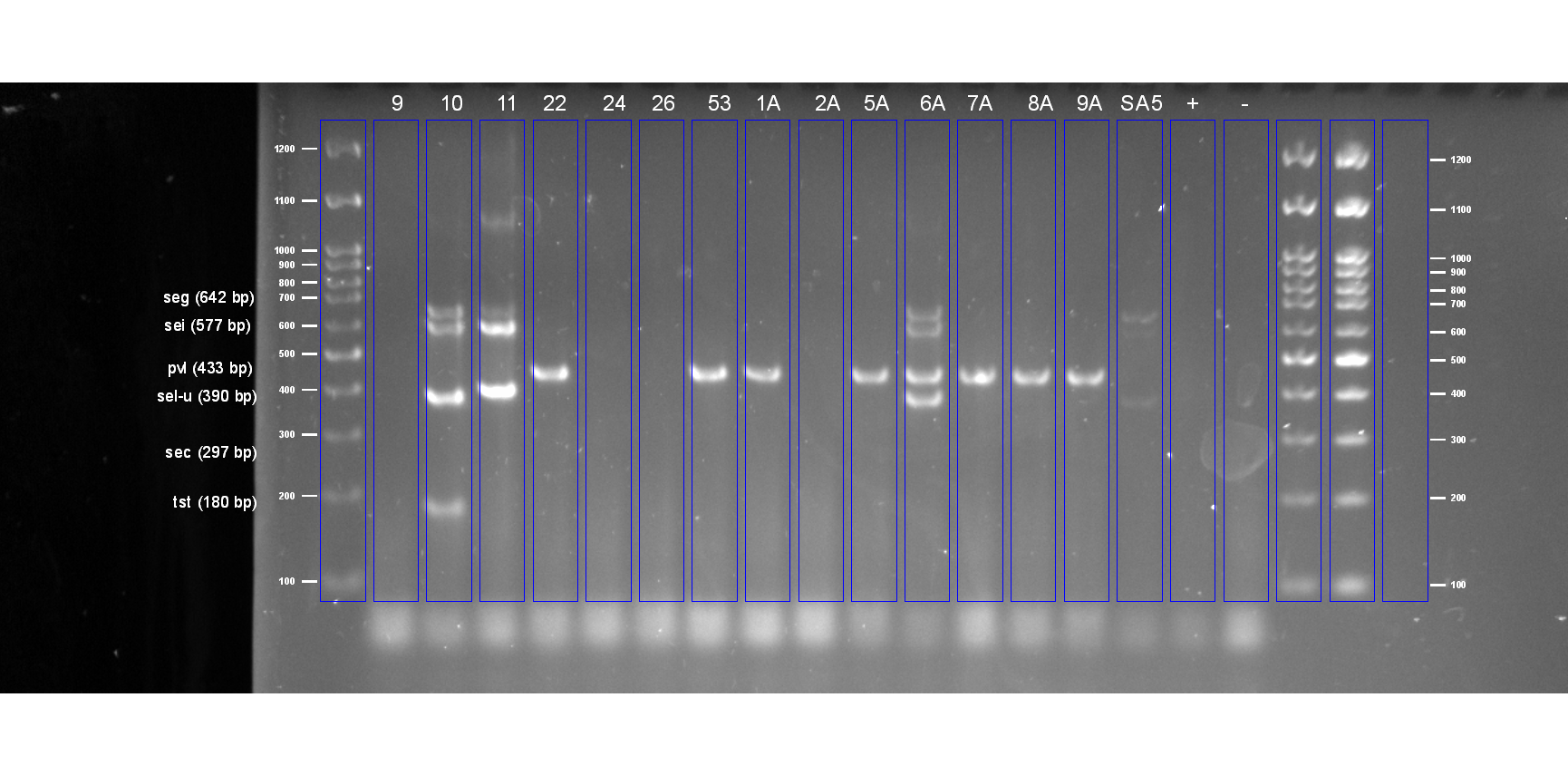

Supplement: Supplementary file 1 — Supplementary file1 (ZIP 25298 KB) [file 11274_2026_5145_MOESM1_ESM.zip › Multiplex PCR_Virulence_tst, pvl, sec, sel-u, seg, sei (#9-11, 22, 24, 26, 53, 1A-2A, 5A-9A).tif]

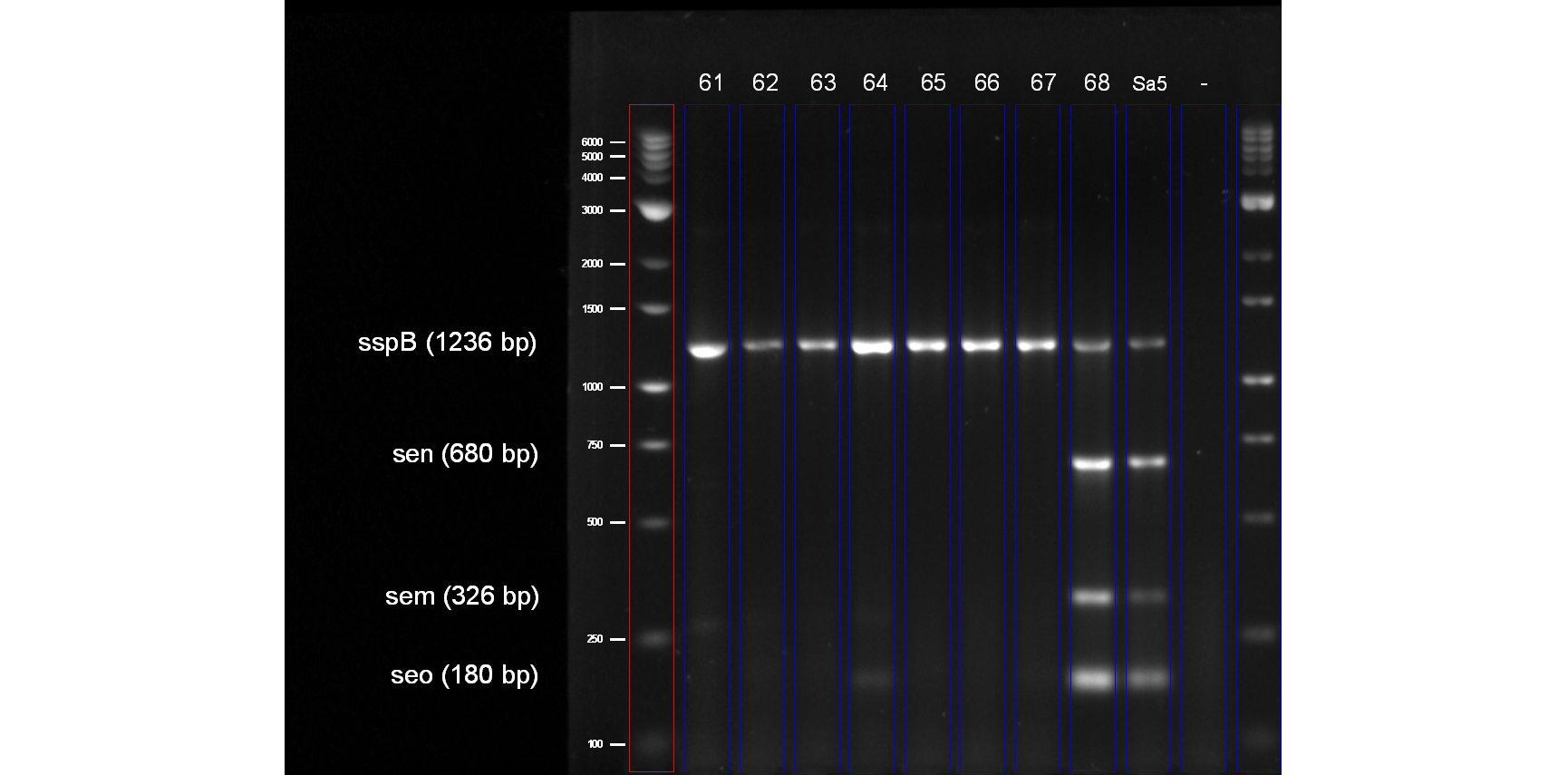

Supplement: Supplementary file 1 — Supplementary file1 (ZIP 25298 KB) [file 11274_2026_5145_MOESM1_ESM.zip › Multiplex PCR_Virulence_SSPB, SEM, SEO, SEN (#61-68).tif]

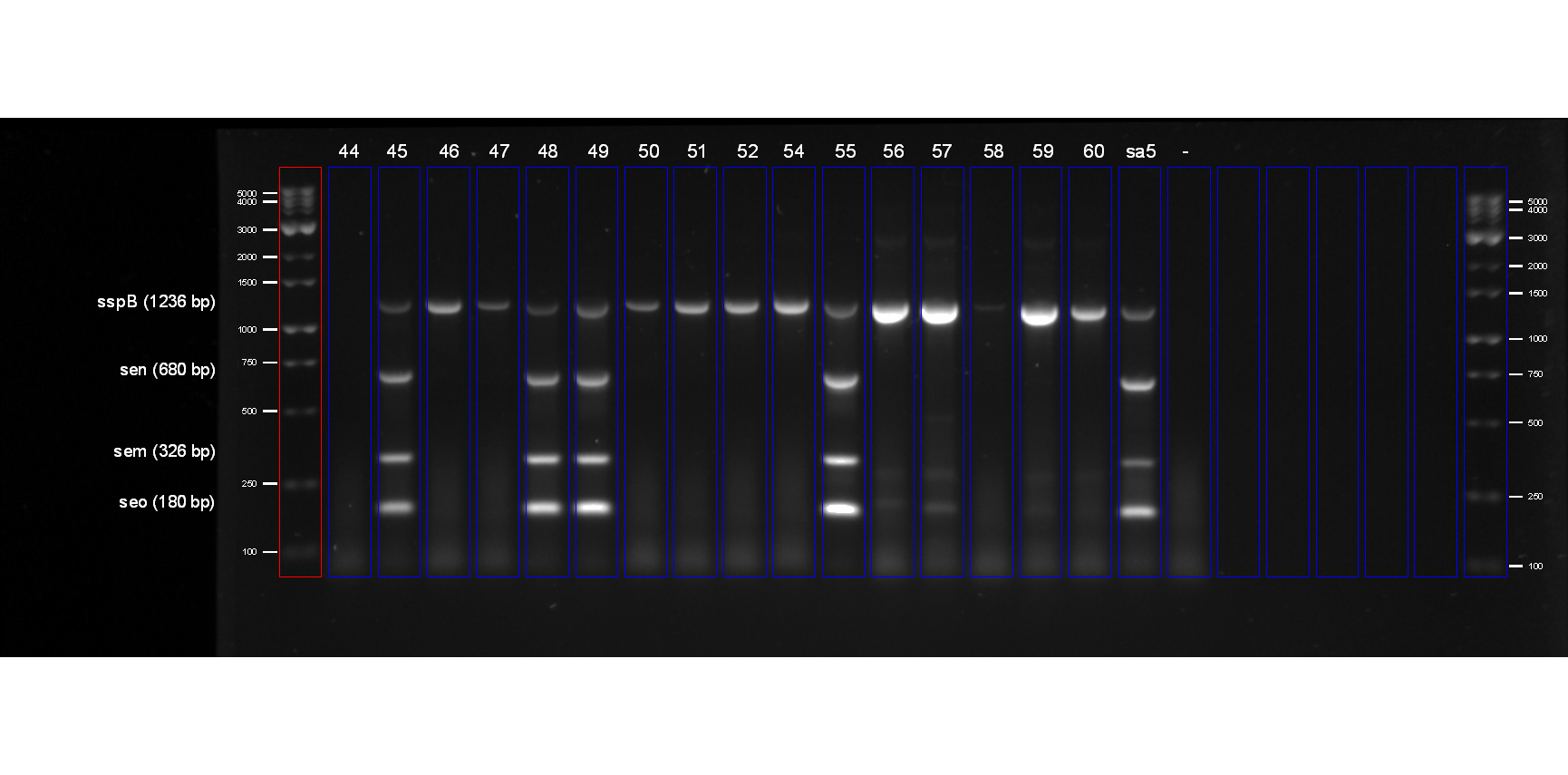

Supplement: Supplementary file 1 — Supplementary file1 (ZIP 25298 KB) [file 11274_2026_5145_MOESM1_ESM.zip › Multiplex PCR_Virulence_SSPB, SEM, SEO, SEN (#44-52, 54-60).tif]

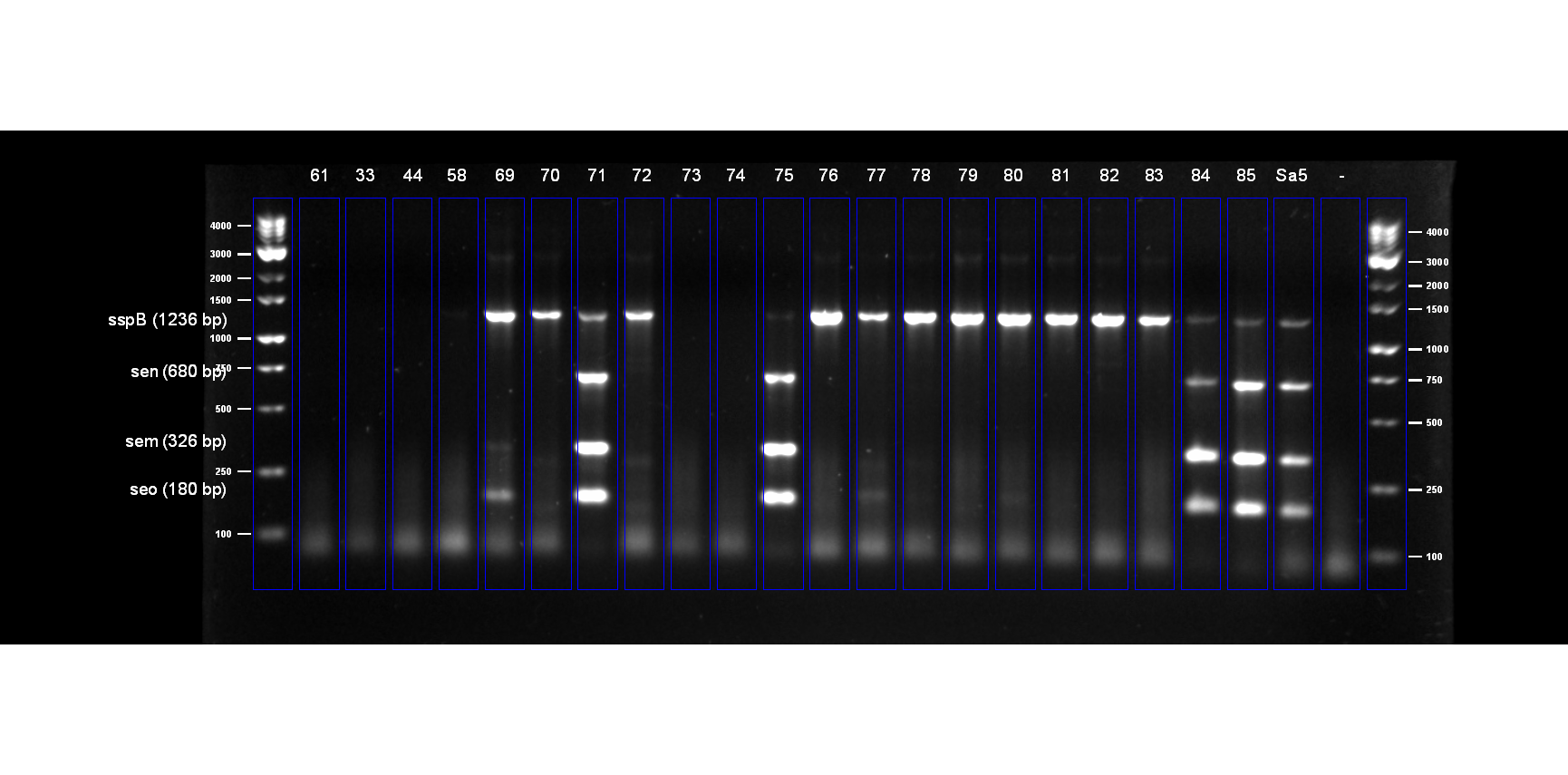

Supplement: Supplementary file 1 — Supplementary file1 (ZIP 25298 KB) [file 11274_2026_5145_MOESM1_ESM.zip › Multiplex PCR_Virulence_SSPB, SEM, SEO, SEN (#31, 33, 44, 58, 69-85).tif]

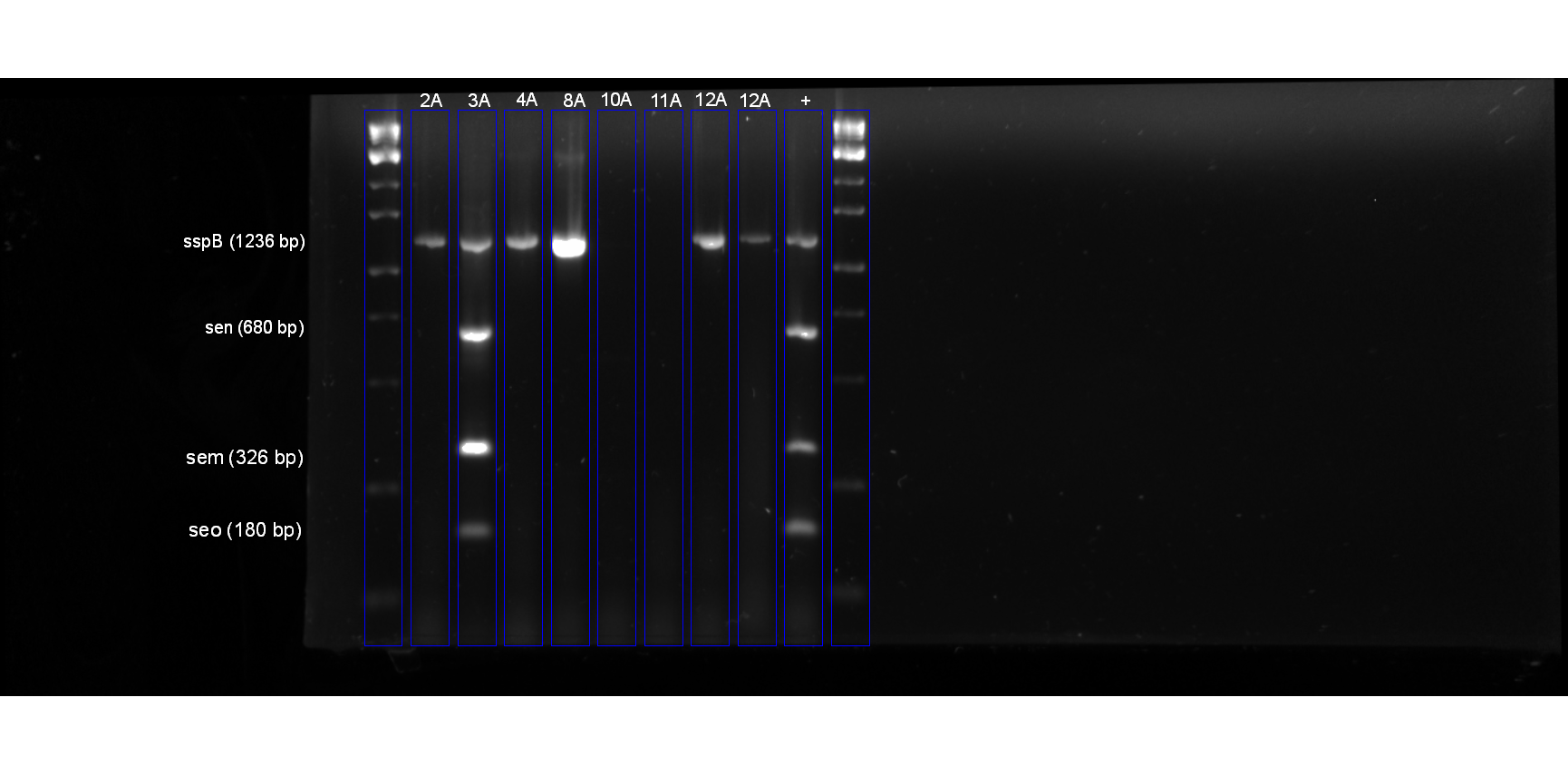

Supplement: Supplementary file 1 — Supplementary file1 (ZIP 25298 KB) [file 11274_2026_5145_MOESM1_ESM.zip › Multiplex PCR_Virulence_SSPB, SEM, SEO, SEN (#2A-12A).tif]

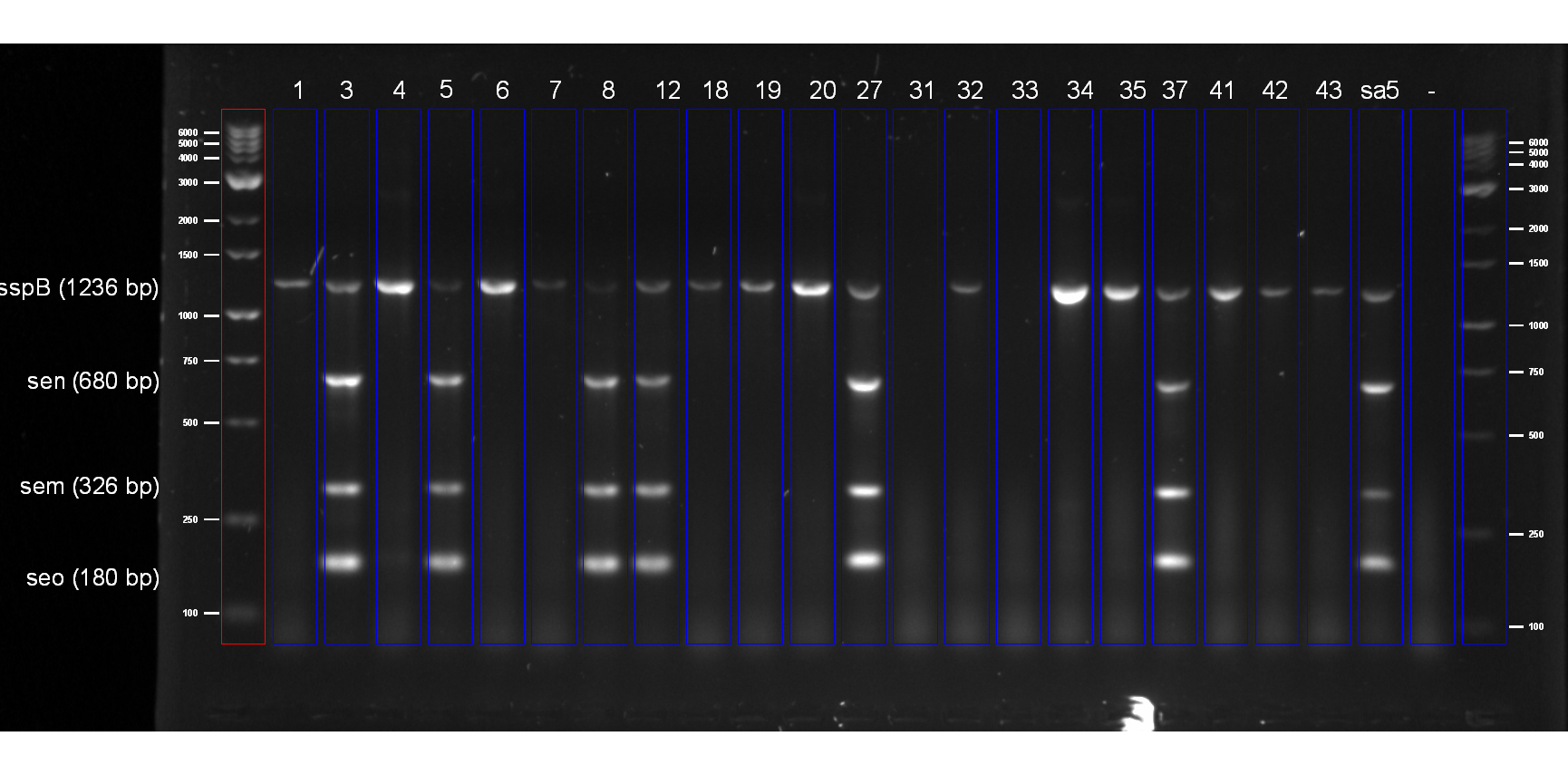

Supplement: Supplementary file 1 — Supplementary file1 (ZIP 25298 KB) [file 11274_2026_5145_MOESM1_ESM.zip › Multiplex PCR_Virulence_SSPB, SEM, SEO, SEN (#1, 3-8, 12, 18-20,27, 31-35, 37, 41-43).tif]

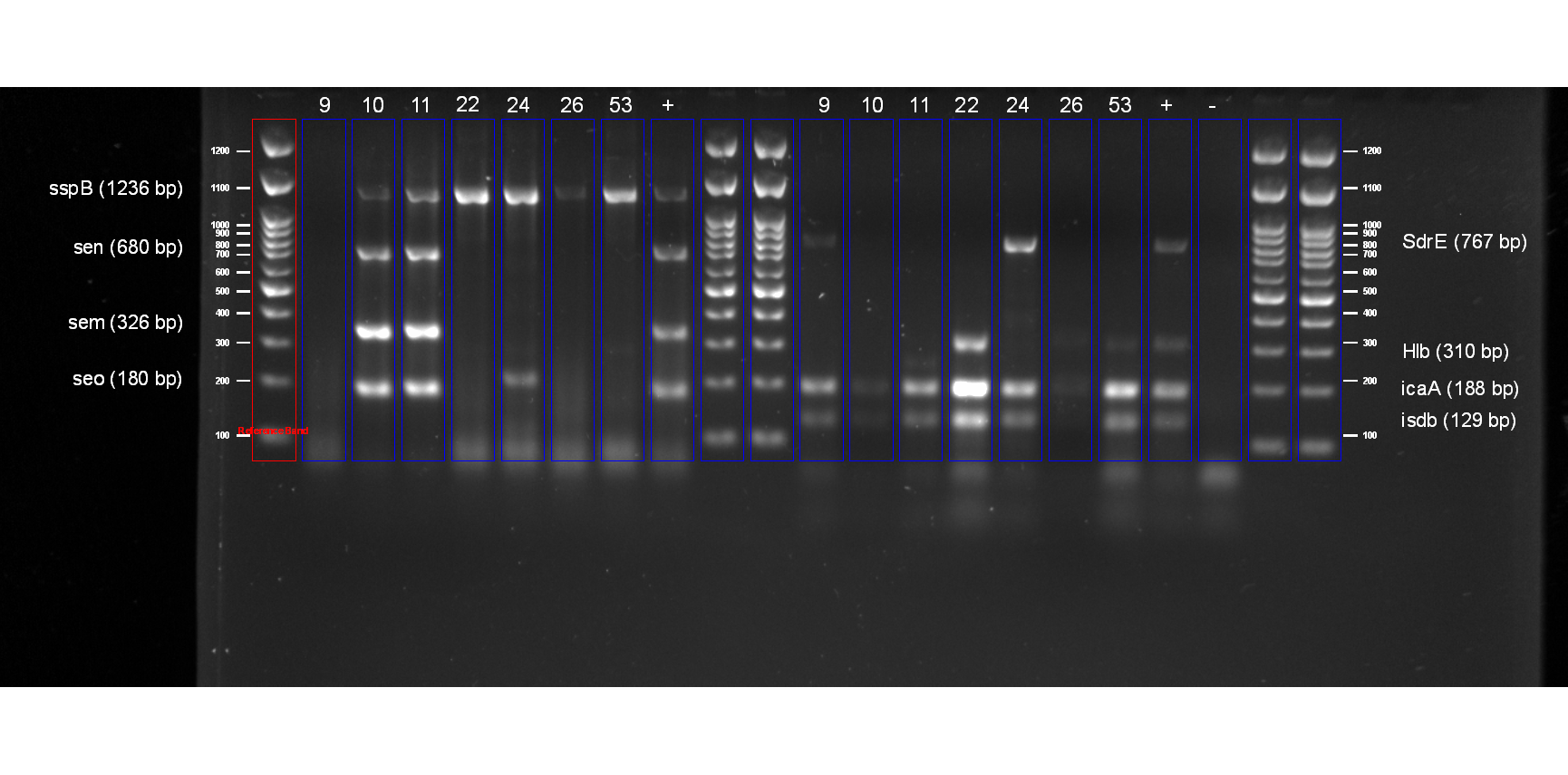

Supplement: Supplementary file 1 — Supplementary file1 (ZIP 25298 KB) [file 11274_2026_5145_MOESM1_ESM.zip › Multiplex PCR_Virulence_sem, sen, seo, sspb (#9-11, 22,24,26,53)_IsdB SdrE, icaA, Hlb (#9-11,22,24,26,53).tif]

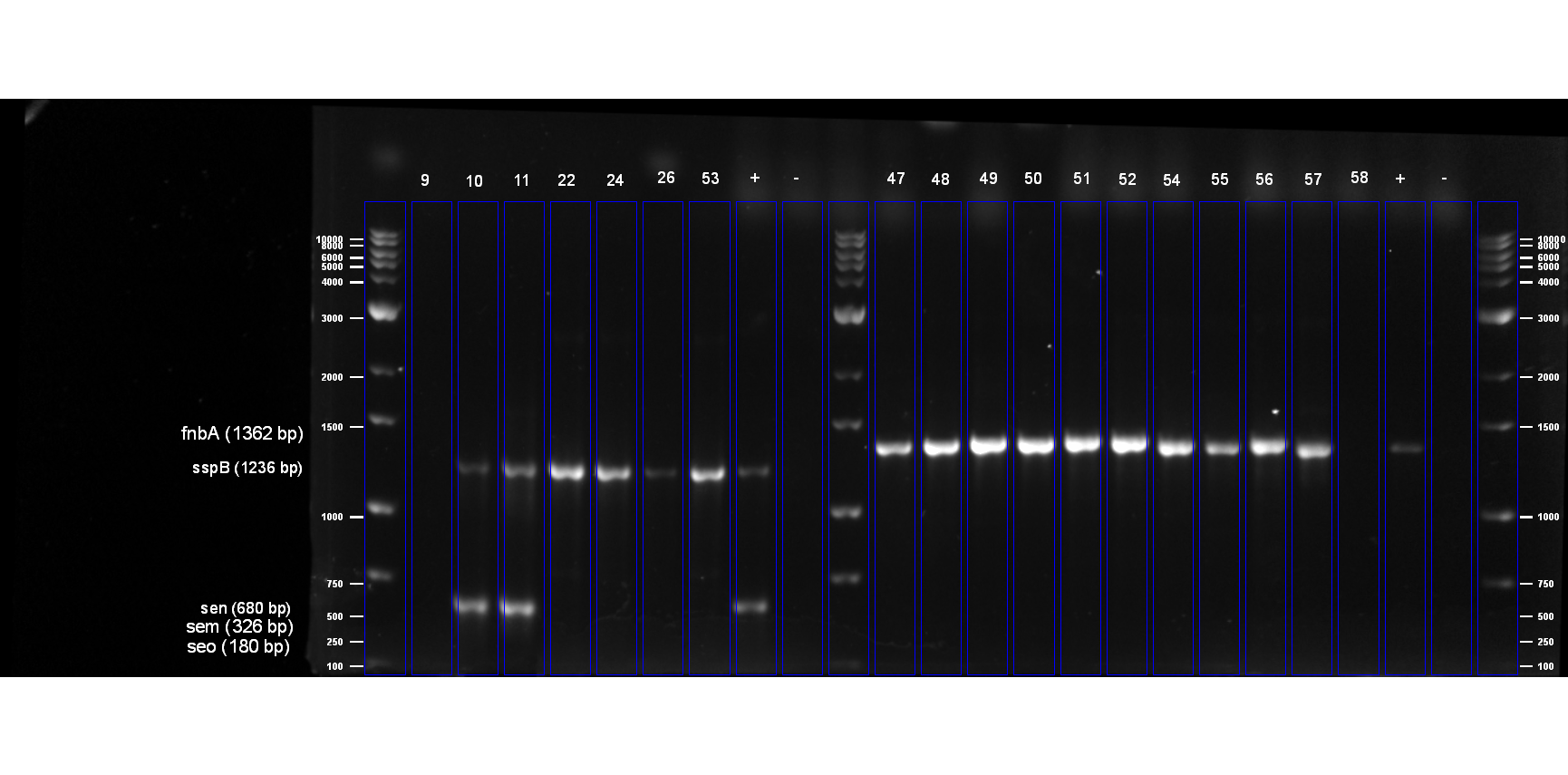

Supplement: Supplementary file 1 — Supplementary file1 (ZIP 25298 KB) [file 11274_2026_5145_MOESM1_ESM.zip › Multiplex PCR_Virulence_sem, sen, seo, ssbp (#9-11, 22, 24, 26, 53)_ fnbp (#47-52, 54-58).tif]

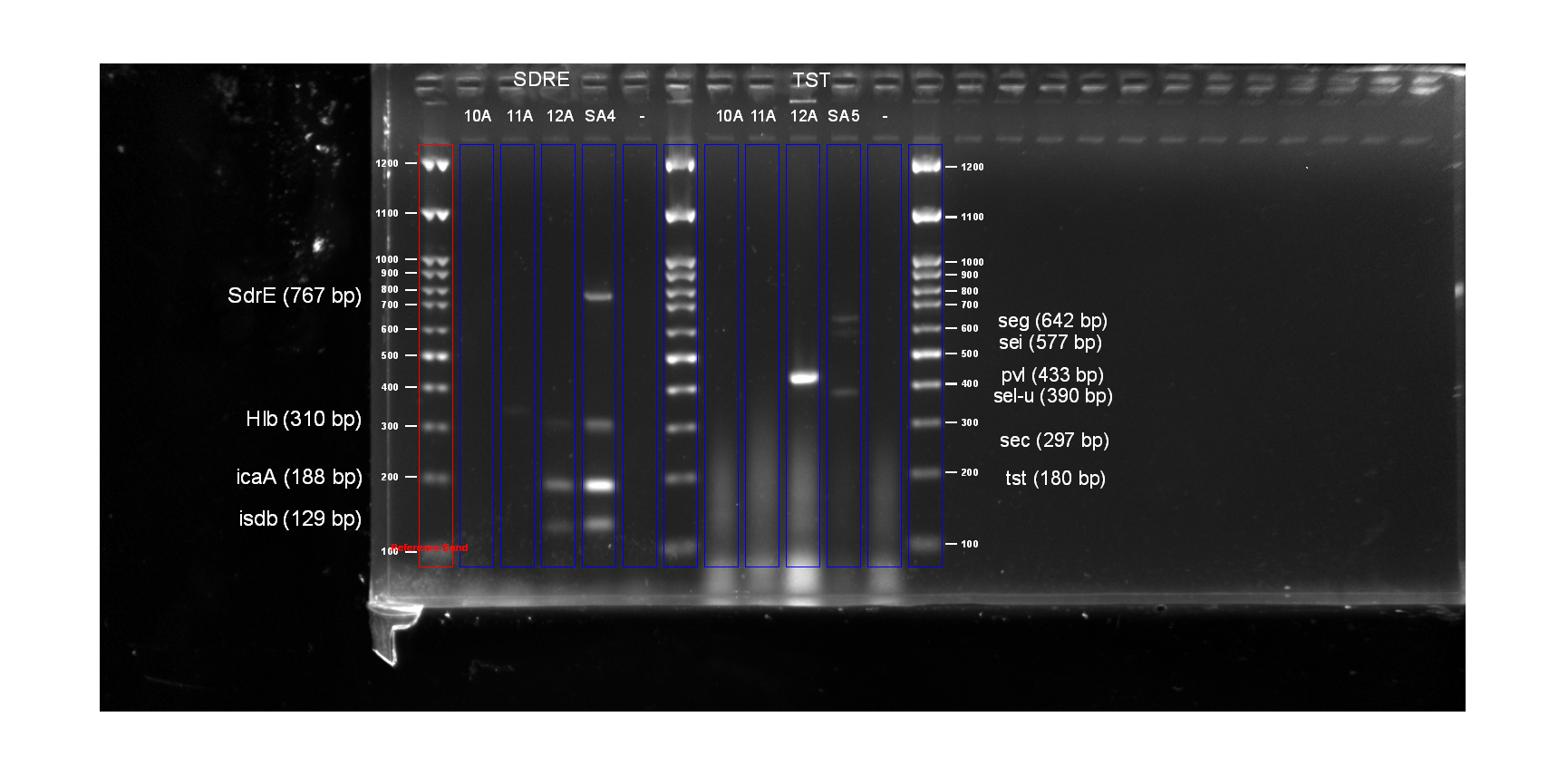

Supplement: Supplementary file 1 — Supplementary file1 (ZIP 25298 KB) [file 11274_2026_5145_MOESM1_ESM.zip › Multiplex PCR_Virulence_SDRE, HLB, ICAA, ISDB, SEG, SEI, PVL, SEL-U, SEC, TST (#10A-12A).tif]

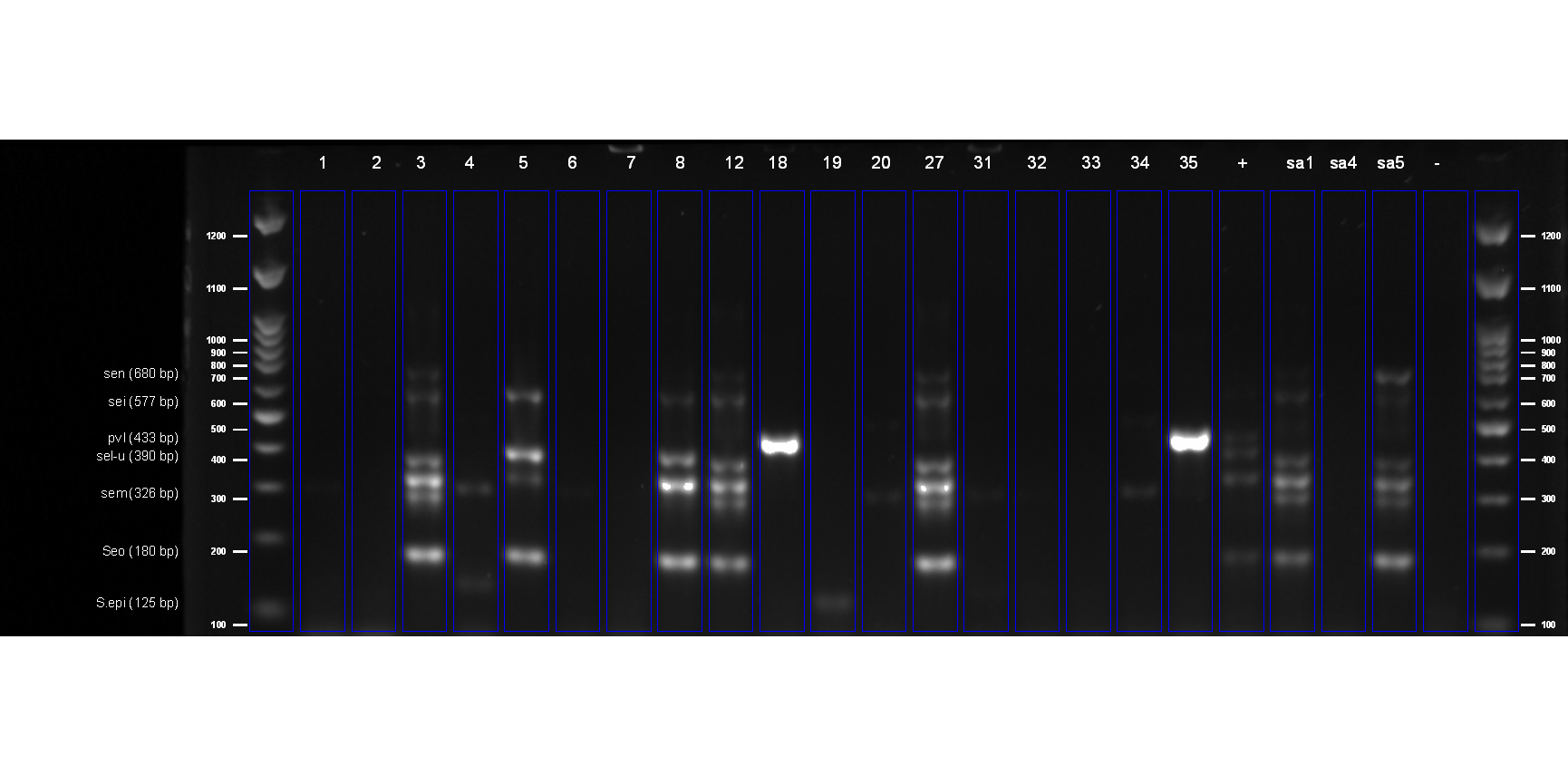

Supplement: Supplementary file 1 — Supplementary file1 (ZIP 25298 KB) [file 11274_2026_5145_MOESM1_ESM.zip › Multiplex PCR_Virulence_S. EPI, SEO, SEM, SEL-U, PVL, SEI, SEN (#1-8, 12, 18-20, 27, 31-35).tif]

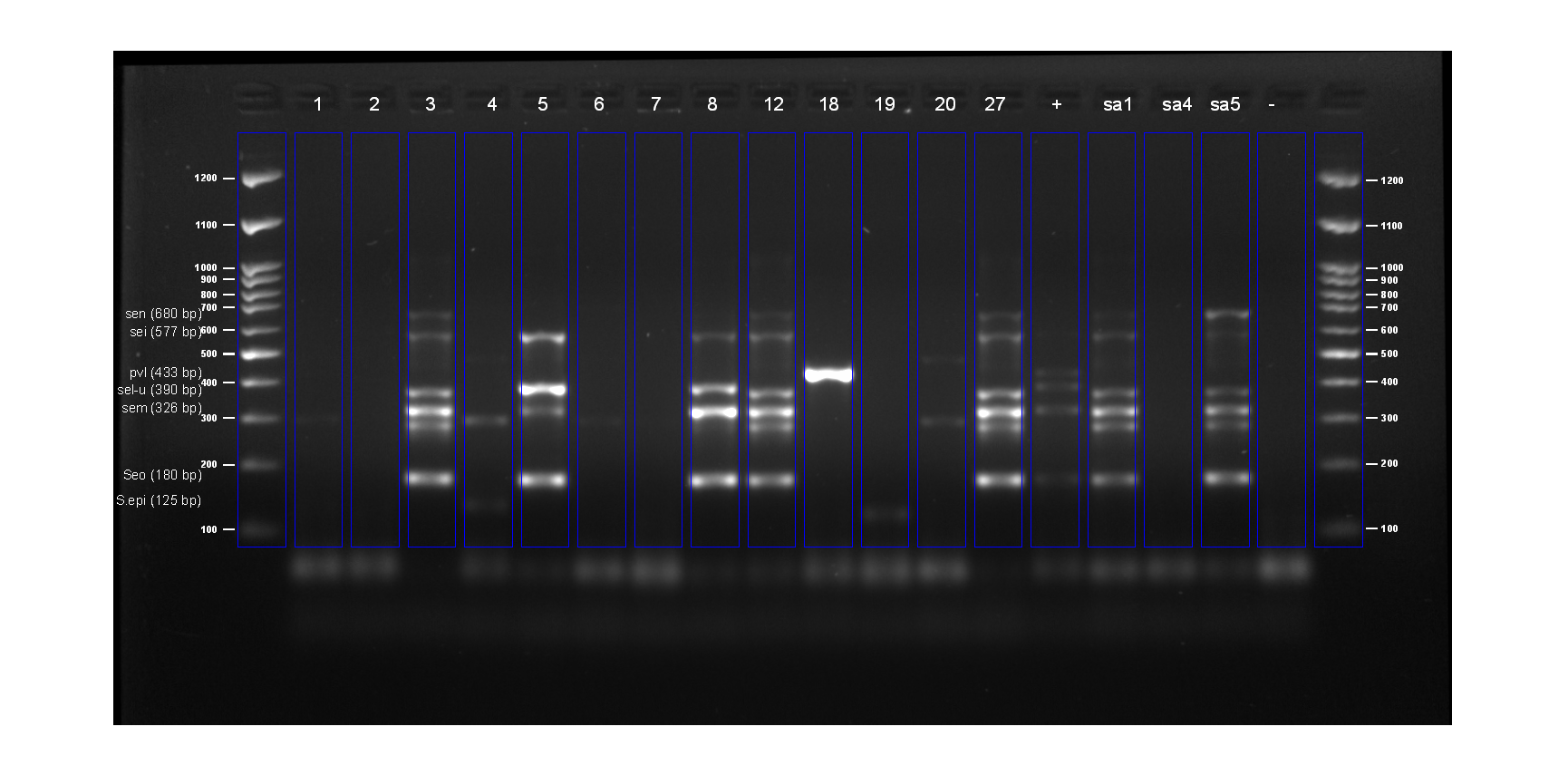

Supplement: Supplementary file 1 — Supplementary file1 (ZIP 25298 KB) [file 11274_2026_5145_MOESM1_ESM.zip › Multiplex PCR_Virulence_S. EPI, SEO, SEM, SEL-U, PVL, SEI, SEN (#1-8, 12, 18-20, 27).tif]

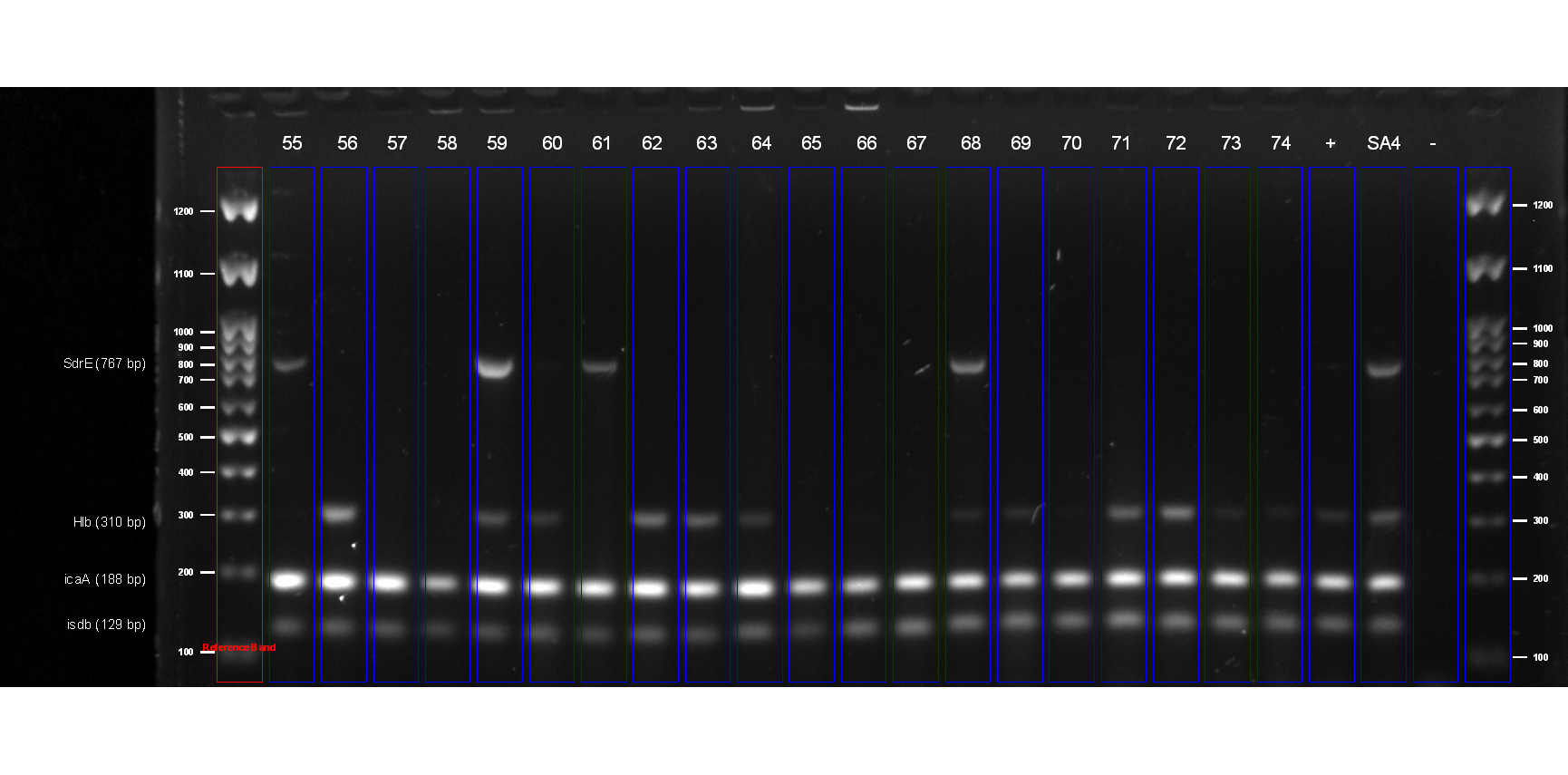

Supplement: Supplementary file 1 — Supplementary file1 (ZIP 25298 KB) [file 11274_2026_5145_MOESM1_ESM.zip › Multiplex PCR_Virulence_IsdB, SdrE, icaA, Hlb (#55-74).tif]

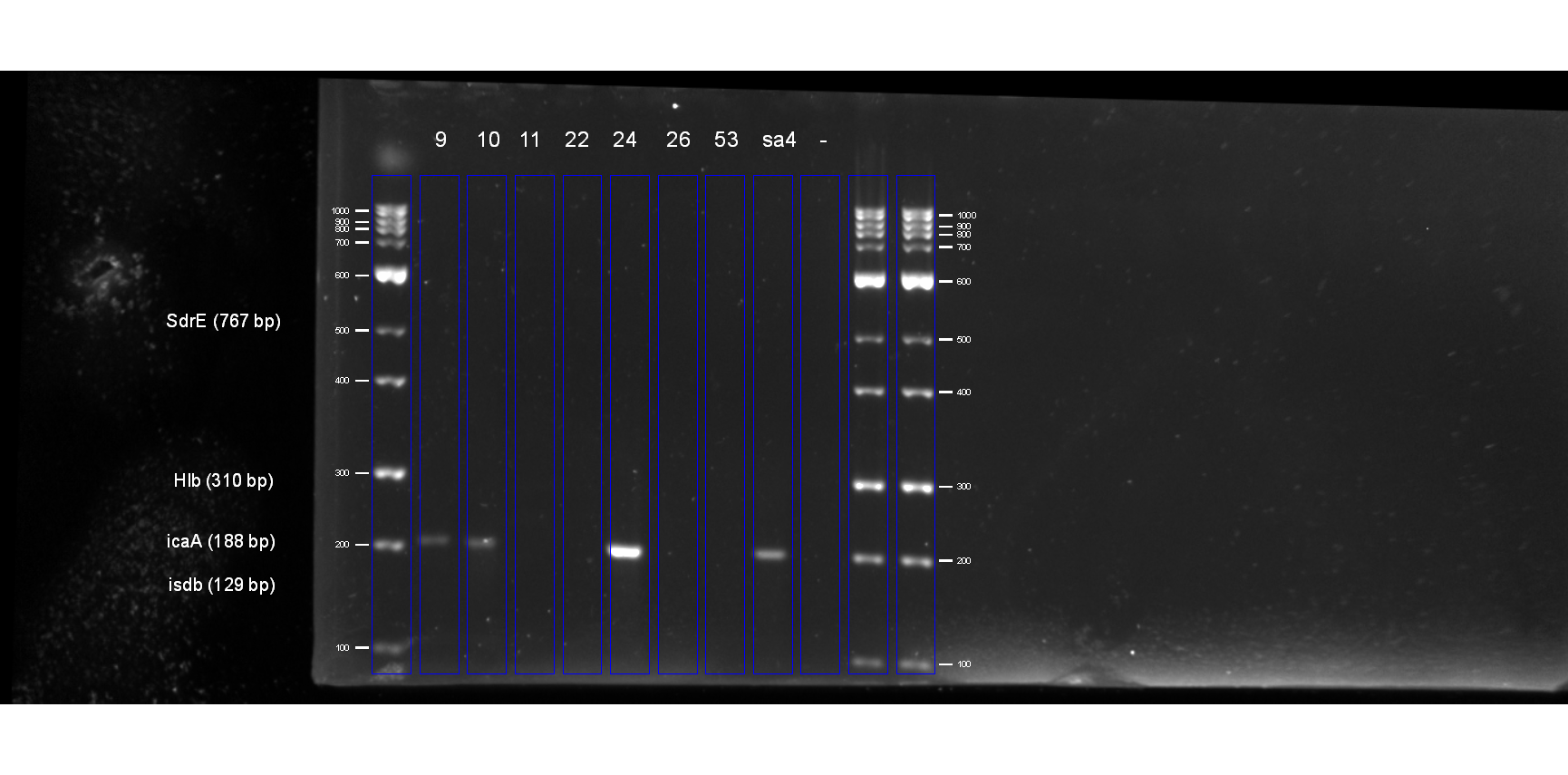

Supplement: Supplementary file 1 — Supplementary file1 (ZIP 25298 KB) [file 11274_2026_5145_MOESM1_ESM.zip › Multiplex PCR_Virulence_IsdB, SdrE, icaA, Hlb (#9-11, 22,24, 26, 53).tif]

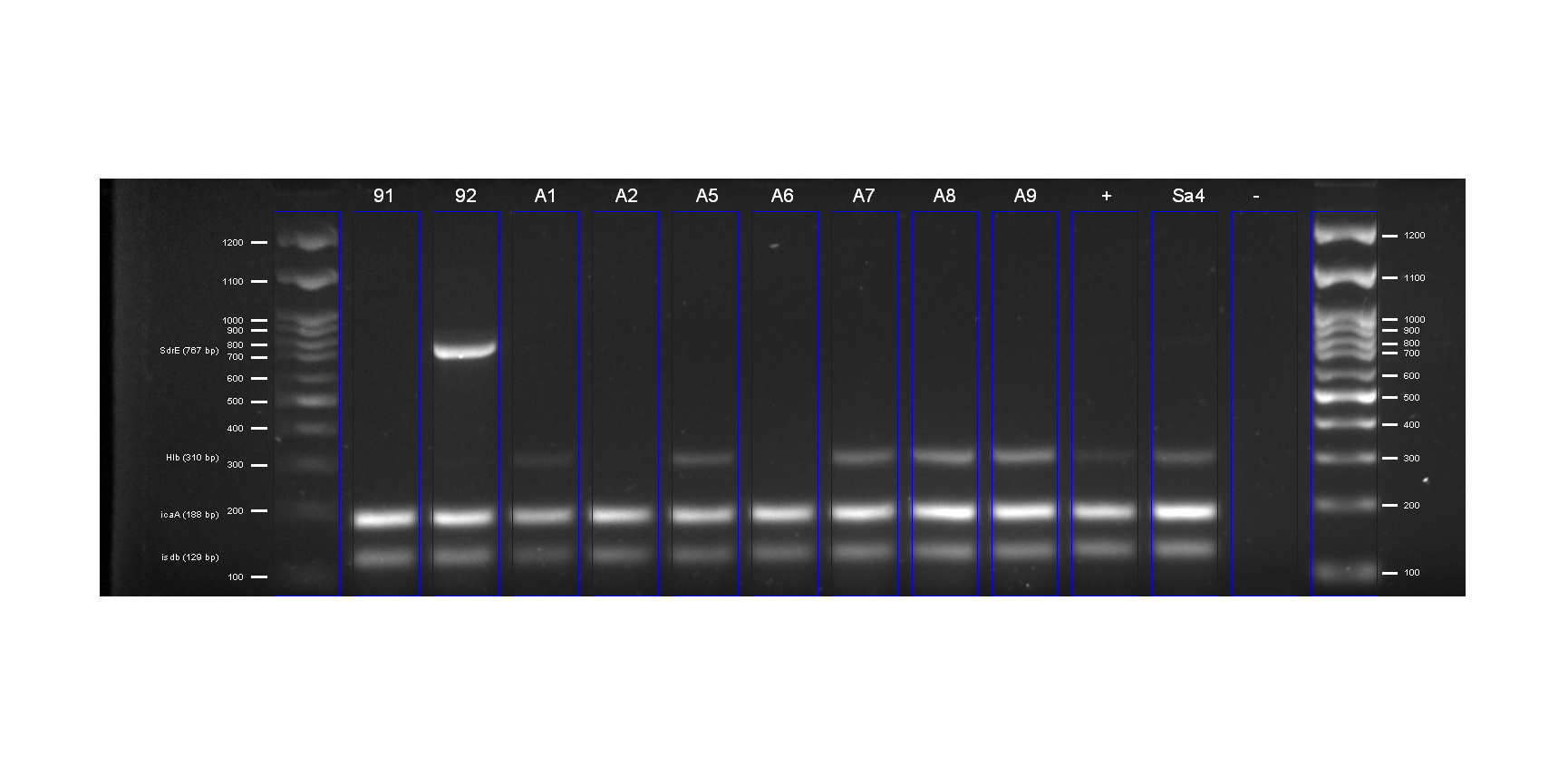

Supplement: Supplementary file 1 — Supplementary file1 (ZIP 25298 KB) [file 11274_2026_5145_MOESM1_ESM.zip › Multiplex PCR_Virulence_IsdB, icaA, Hlb, SdrE (#91-92, A1, a2, A5, 6A-9A).tif]

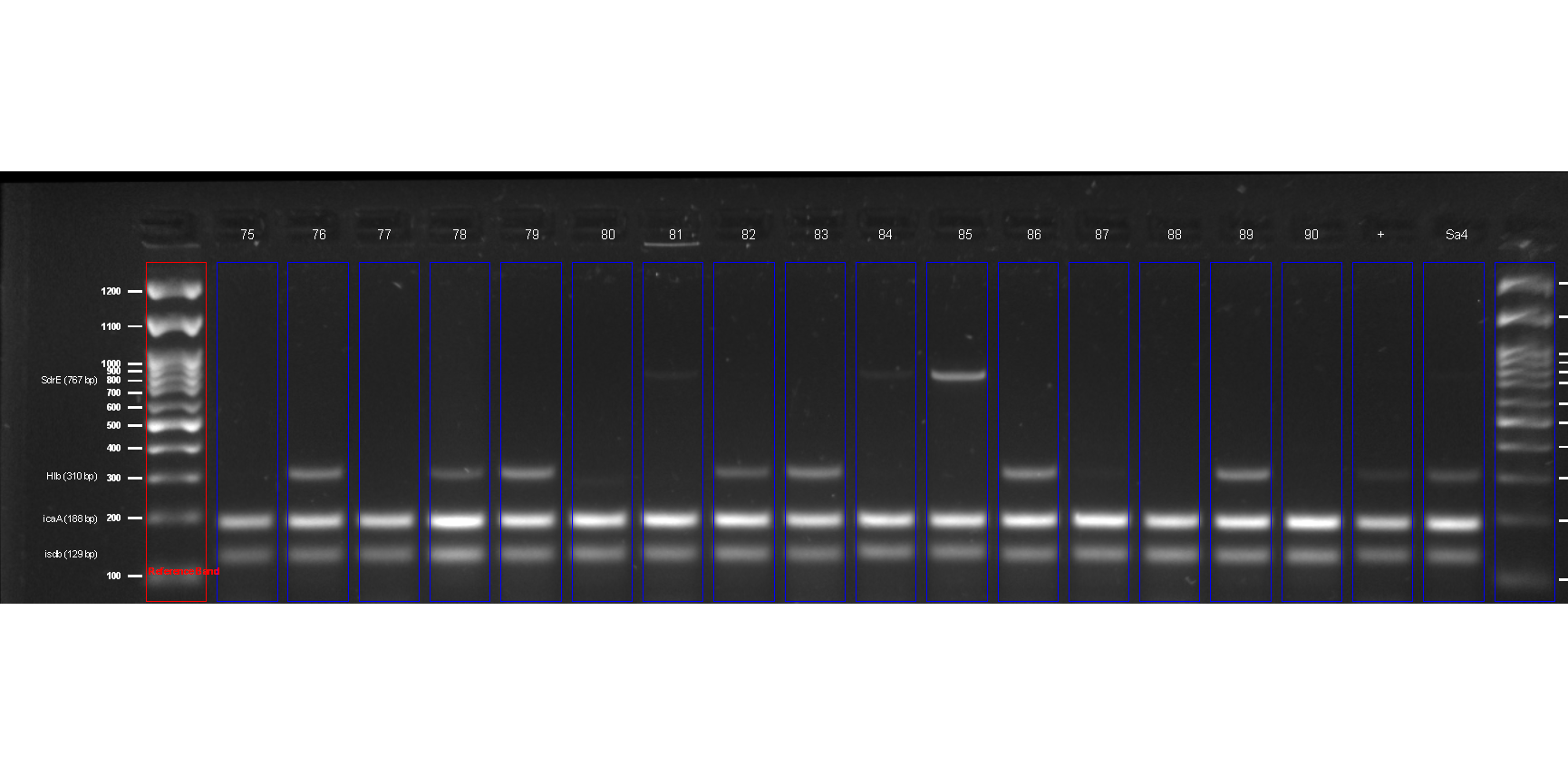

Supplement: Supplementary file 1 — Supplementary file1 (ZIP 25298 KB) [file 11274_2026_5145_MOESM1_ESM.zip › Multiplex PCR_Virulence_IsdB, icaA, Hlb, SdrE (#75-90).tif]

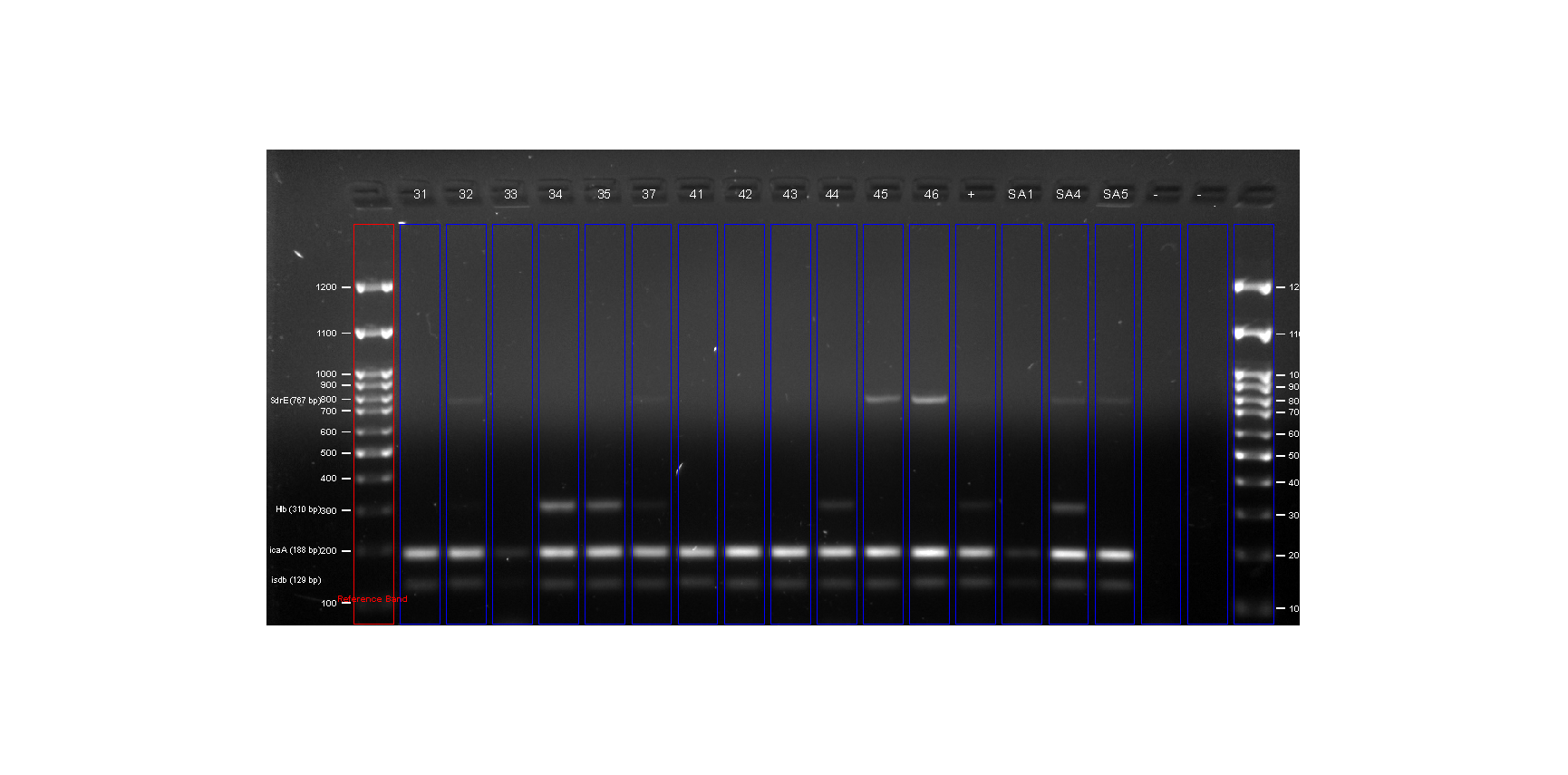

Supplement: Supplementary file 1 — Supplementary file1 (ZIP 25298 KB) [file 11274_2026_5145_MOESM1_ESM.zip › Multiplex PCR_Virulence_IsdB, icaA, Hlb, SdrE (#31-35, 37, 41-46).tif]

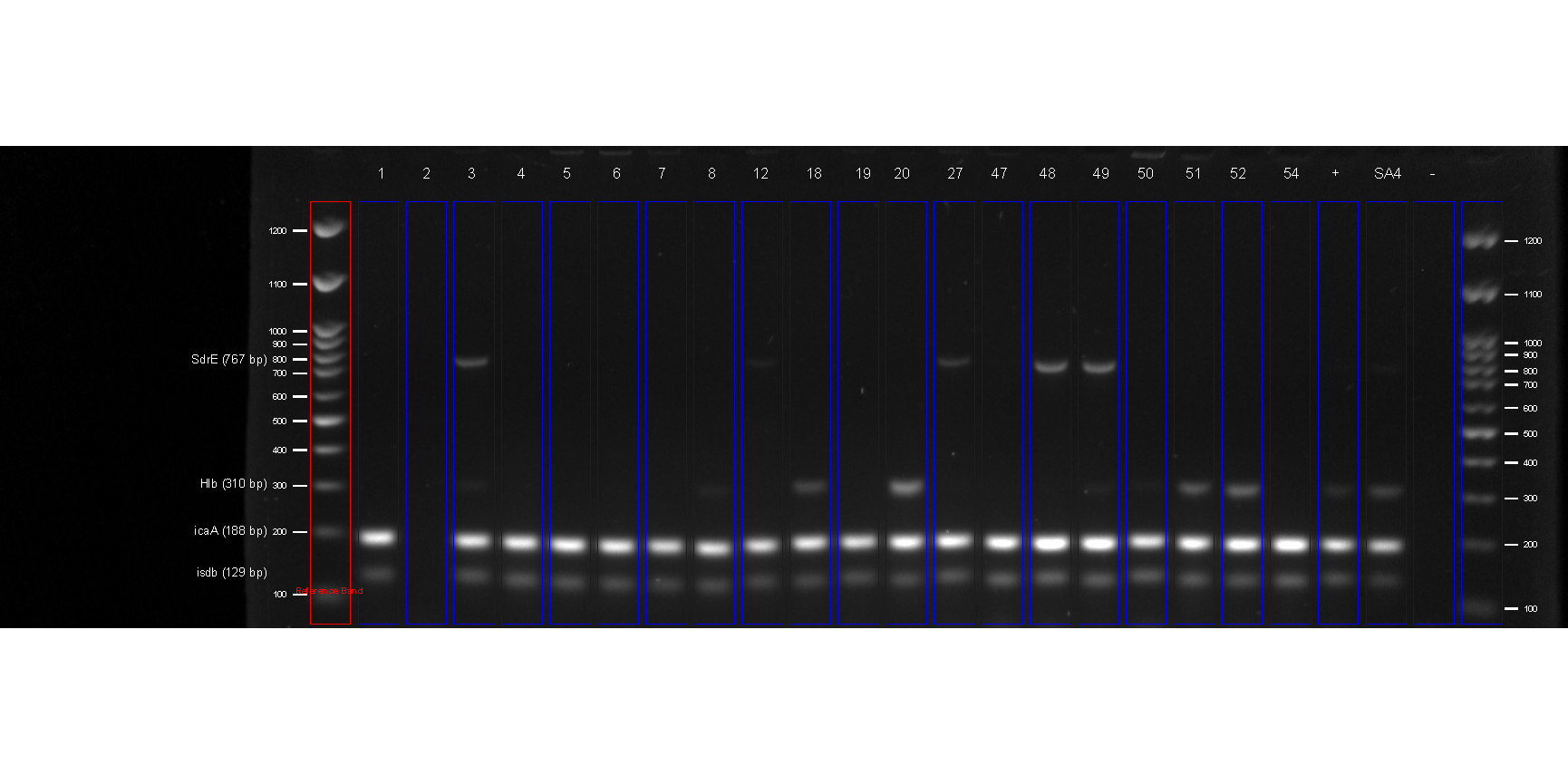

Supplement: Supplementary file 1 — Supplementary file1 (ZIP 25298 KB) [file 11274_2026_5145_MOESM1_ESM.zip › Multiplex PCR_Virulence_IsdB, icaA, Hlb, SdrE (#1-8, 12, 18-20, 27, 47-54).tif]

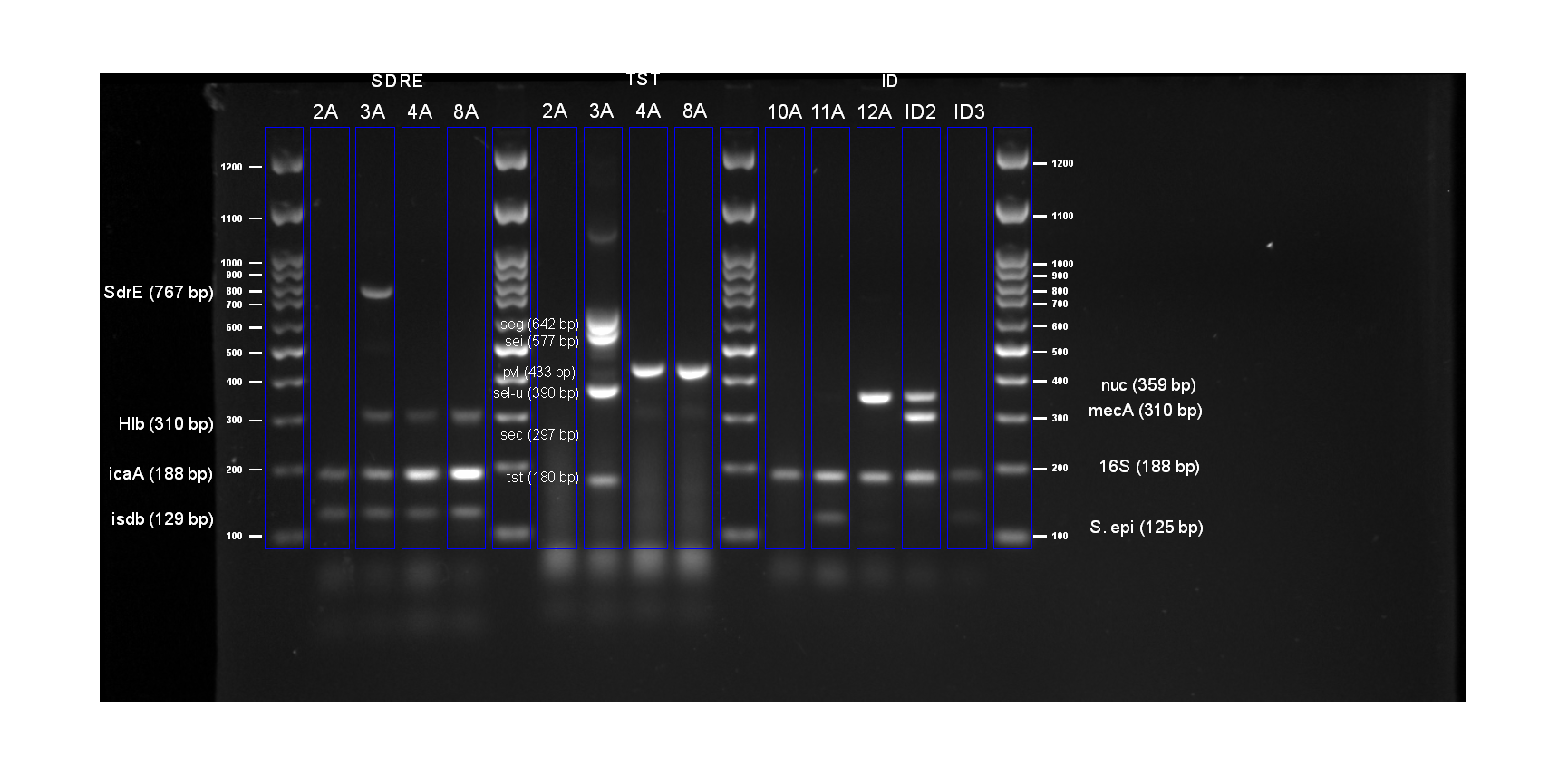

Supplement: Supplementary file 1 — Supplementary file1 (ZIP 25298 KB) [file 11274_2026_5145_MOESM1_ESM.zip › Multiplex PCR_ID & Virulence_.tif]

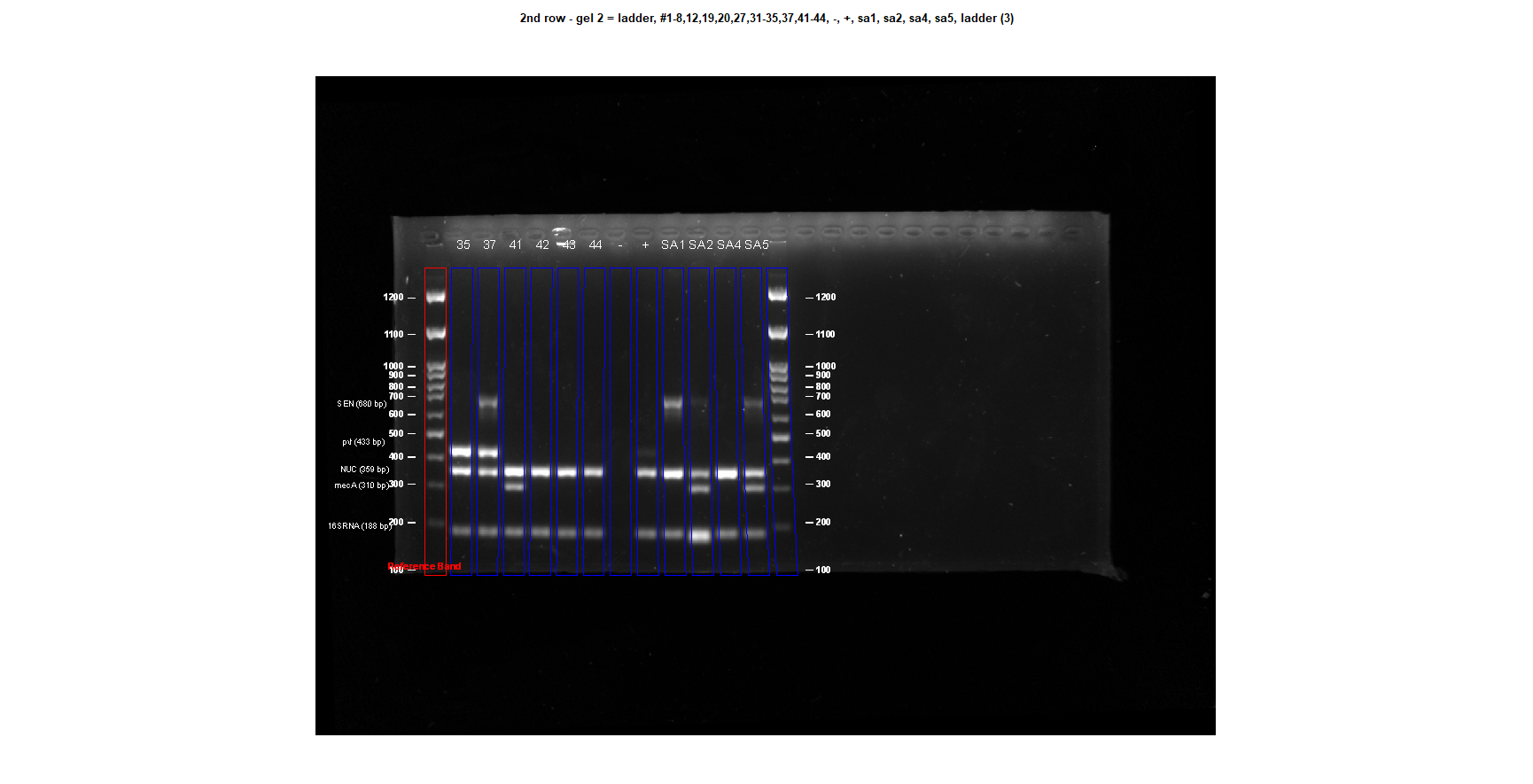

Supplement: Supplementary file 1 — Supplementary file1 (ZIP 25298 KB) [file 11274_2026_5145_MOESM1_ESM.zip › ID + virulence (#1-8,12,19,20,27,31-35,37,41-44,).tif]

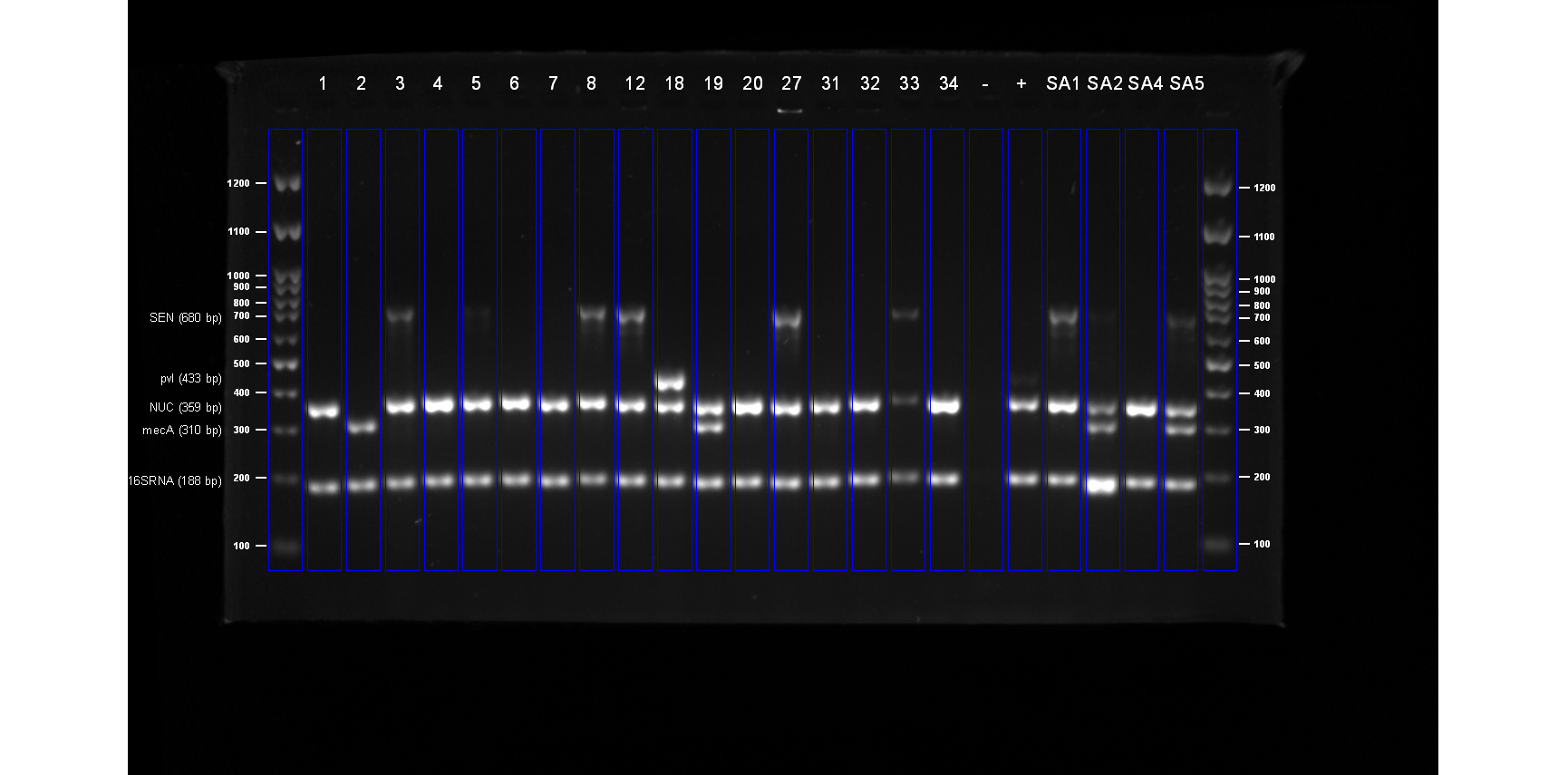

Supplement: Supplementary file 1 — Supplementary file1 (ZIP 25298 KB) [file 11274_2026_5145_MOESM1_ESM.zip › ID + virulence (#1-8,12,19,20,27,31-34).tif]

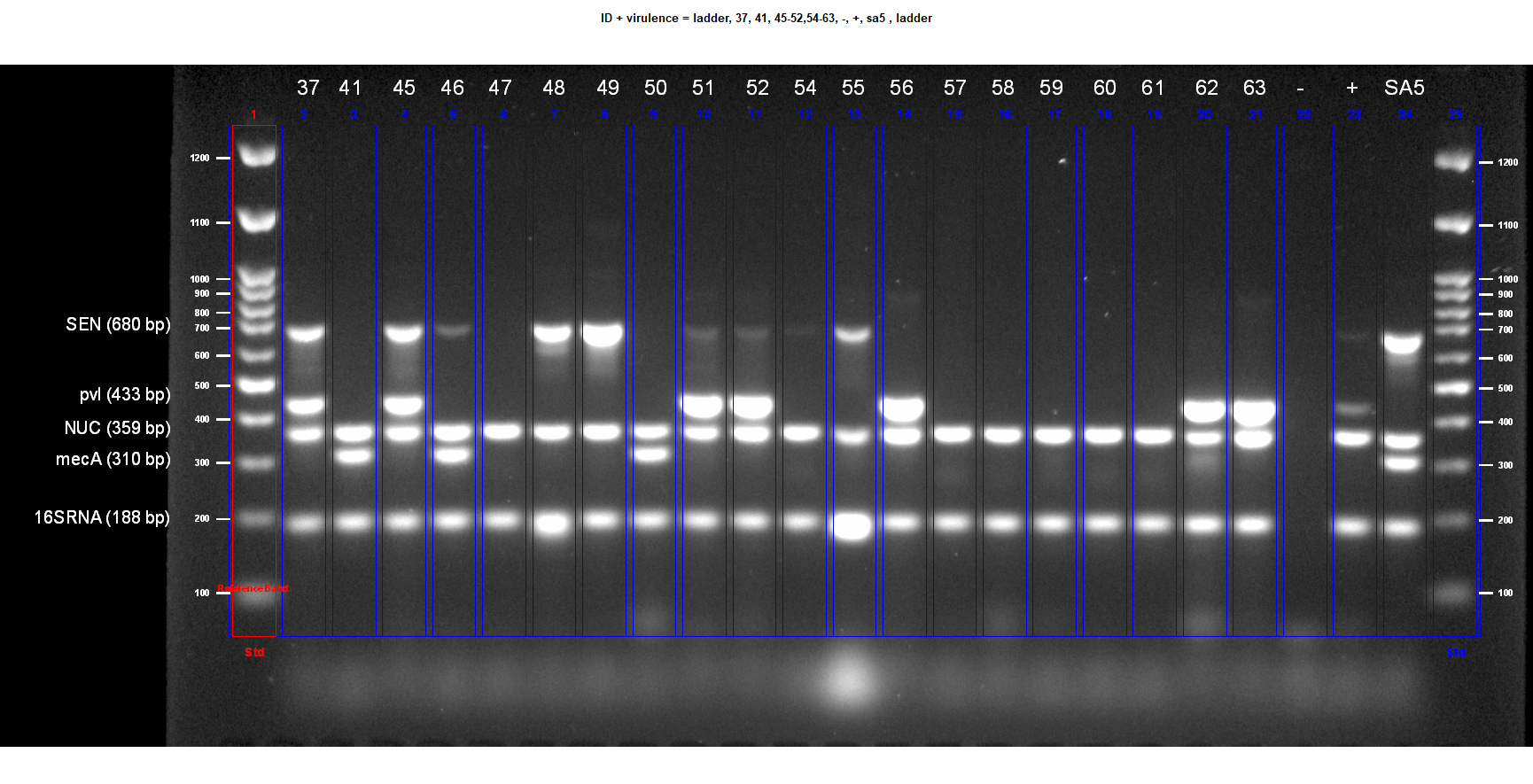

Supplement: Supplementary file 1 — Supplementary file1 (ZIP 25298 KB) [file 11274_2026_5145_MOESM1_ESM.zip › ID + virulence (37, 41, 45-52,54-63).tif]

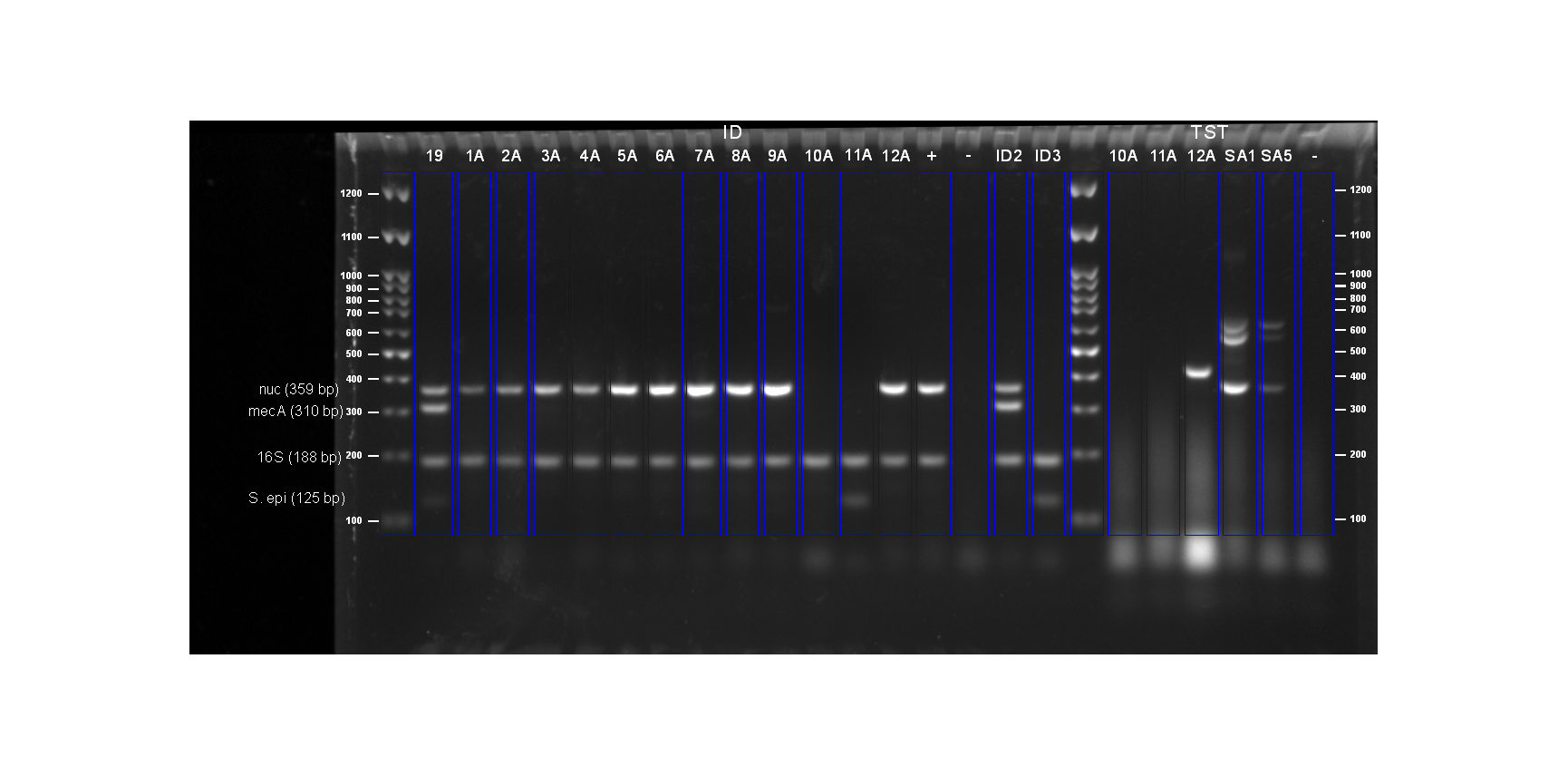

Supplement: Supplementary file 1 — Supplementary file1 (ZIP 25298 KB) [file 11274_2026_5145_MOESM1_ESM.zip › ID + virulence (19, 1A-12A.tif]
